# NoPv1: a synthetic antimicrobial peptide aptamer targeting the causal agents of grapevine downy mildew and potato late blight

**DOI:** 10.1038/s41598-020-73027-x

**Published:** 2020-10-16

**Authors:** Monica Colombo, Simona Masiero, Stefano Rosa, Elisabetta Caporali, Silvia Laura Toffolatti, Chiara Mizzotti, Luca Tadini, Fabio Rossi, Sara Pellegrino, Rita Musetti, Riccardo Velasco, Michele Perazzolli, Silvia Vezzulli, Paolo Pesaresi

**Affiliations:** 1 Research and Innovation Centre, Fondazione Edmund Mach, San Michele all’Adige, Italy; 2 Department of Biosciences, University of Milan, Milan, Italy; 3 Department of Agricultural and Environmental Sciences (DISAA), University of Milan, Milan, Italy; 4 Center for Study and Research on Obesity, Department of Medical Biotechnology and Translational Medicine, University of Milan, Milan, Italy; 5 DISFARM-Department of Pharmaceutical Sciences, University of Milan, Milan, Italy; 6 Department of Agricultural, Food, Environmental and Animal Sciences, University of Udine, Udine, Italy; 7 CREA Research Centre for Viticulture and Enology, Conegliano, TV Italy; 8 Centre Agriculture Food Environment (C3A), University of Trento, San Michele all’Adige, Italy

**Keywords:** Biotechnology, Peptide delivery, Plant biotechnology

## Abstract

Grapevine (*Vitis vinifera* L.) is a crop of major economic importance. However, grapevine yield is guaranteed by the massive use of pesticides to counteract pathogen infections. Under temperate-humid climate conditions, downy mildew is a primary threat for viticulture. Downy mildew is caused by the biotrophic oomycete *Plasmopara viticola* Berl. & de Toni, which can attack grapevine green tissues. In lack of treatments and with favourable weather conditions, downy mildew can devastate up to 75% of grape cultivation in one season and weaken newly born shoots, causing serious economic losses. Nevertheless, the repeated and massive use of some fungicides can lead to environmental pollution, negative impact on non-targeted organisms, development of resistance, residual toxicity and can foster human health concerns. In this manuscript, we provide an innovative approach to obtain specific pathogen protection for plants. By using the yeast two-hybrid approach and the *P. viticola* cellulose synthase 2 (*Pv*CesA2), as target enzyme, we screened a combinatorial 8 amino acid peptide library with the aim to identify interacting peptides, potentially able to inhibit *Pv*Cesa2. Here, we demonstrate that the NoPv1 peptide aptamer prevents *P. viticola* germ tube formation and grapevine leaf infection without affecting the growth of non-target organisms and without being toxic for human cells. Furthermore, NoPv1 is also able to counteract *Phytophthora infestans* growth, the causal agent of late blight in potato and tomato, possibly as a consequence of the high amino acid sequence similarity between *P. viticola* and *P. infestans* cellulose synthase enzymes.

## Introduction

Pesticide use is at the basis of intensive agriculture, as it guarantees protection from pathogens, pests and weeds, which otherwise would count for up to 40% of production losses^1–3^. In the European Union (EU), almost 500 active substances, most of them able to inhibit key metabolic pathways of plant pathogens^4^, are approved as pesticides (European commission, EU pesticides database, 2018; ec.europa.eu/food/plant/pesticides/eu-pesticides-database), with annual sales of 374,000 tons (Eurostat. Pesticide sales, 2018; ec.europa.eu/eurostat/web/products-datasets/-/aei_fm_salpest09), whereas the global use of pesticides accounted for 4,113,591 tonnes in 2017 (FAOSTAT, fao.org/faostat/en/#data/RP/visualize). However, despite the beneficial effects on agricultural production, some pesticides can have a harmful impact on the environment and on the health of humans and animals depending on their concentration, bioavailability and rate of transport through the soil^5–11^. Dermatological, gastrointestinal, carcinogenic, respiratory and endocrine pathologies are among the negative health effects that have been associated with the massive use of chemical pesticides^12^. Residues of pesticides can be found in foods and beverages^13,14^, although in most of the cases the concentrations do not exceed the safe levels^15^. However, the simultaneous exposure to different compounds may have negative synergistic effects^16^. Consequently, stricter regulations have been enacted both in the EU [Sustainable Use Directive 2009/128/EC and Plant Protection Products Regulation (EC) 1107/2009] and in the USA (Insecticide, Fungicide, and Rodenticide Act). Several compounds have been banned or included in a list of candidates for substitution (ec.europa.eu/food/plant/pesticides/approval_active_substances_en) because of problems of environmental toxicity, effects on human health or development of resistance in the target pathogen, similarly to the case of antibiotics^17^.

Taken these considerations together, it appears clear that new biotechnological sustainable solutions need to be explored to find safe and reliable alternatives to conventional pesticides that specifically inhibit the activity of pathogen key enzymes. In recent years, peptide aptamers—i.e. short synthetic peptides able to specifically bind and inhibit a protein target—have emerged as novel molecular tools that have attracted the attention of different research groups interested in developing antimicrobial compounds^18,19^, presumably with a better environmental fate, lower off-target effects and possibly a low-risk alternative to conventional pesticides, although this technology requires experimental validation.

Among crops, grapevine represents a great agricultural and economic value worldwide, with 7.534,00 Mha invested in viticulture^20^. However, the grapevine industry relies predominantly on *Vitis vinifera*, which is susceptible to a large spectrum of pathogens and requires a frequent application of chemical antimicrobials to avoid yield and quality loss^21^. In particular, under temperate-humid climate conditions downy mildew, a worldwide destructive disease caused by the biotrophic oomycete *Plasmopara viticola* Berl. & De Toni^22^, is considered of primary importance for viticulture^20^. In lack of treatments and with favourable weather conditions, downy mildew can devastate up to 75% of the crop in one season and weaken newly emerging shoots, causing serious economic losses^23,24^. *P. viticola* attacks grapevine green organs, such as leaves and young fruits, by releasing flagellate zoospores at high humidity and warm temperatures^25^. When the zoospores encounter a stoma, they shed the flagella and encyst. Subsequently, a germ tube emerges from each spore and reaches the substomatal cavity, where it dilates into a vesicle that initiates the infection^26^.

Data available for the oomycete *Phytophthora infestans*, the causal agent of late blight in potato and tomato^23^, provide useful information on the molecular mechanisms at the basis of oomycete plant infection. The main component of *P. infestans* cell wall is cellulose, accounting for the 33.6% over the total glucan composition (85.6%)^27^. Cellulose appears to be essential for appressorium formation and effective potato infection by *P. infestans*, since inhibition of its biosynthesis leads to a dramatic reduction in the number of normal germ tubes with appressoria, severe disruption of the cell wall in the pre-infection structures, and a complete loss of pathogenicity^28^. Furthermore, cellulose synthase gene expression is up-regulated during pre- and early-infection stages of *P. infestans* in potato^28^. Additionally, *P. infestans* and *P. viticola* cellulose synthase enzymes have been identified as the target of the fungicide mandipropamid, highlighting the relevance of cell wall biosynthesis in oomycete disease development^29,30^.

In this manuscript, we describe the identification of a novel peptide aptamer of 8 amino acid residues, named NoPv1 (No *Plasmopora* *viticola* 1), able to counteract *P. viticola* infection ex vivo (leaf disks) and in vivo (potted plants). In particular, the *Pv*CesA2 (*P. viticola* cellulose synthase 2) enzyme was used as a bait in the yeast two-hybrid assay to isolate the NoPv1 peptide, from a combinatorial peptide library^31^. NoPv1 prevents *P. viticola* germ tube formation and grapevine infection without affecting the growth of off-target organisms, and without being toxic for plant and human cells. Furthermore, NoPv1 is also able to arrest the growth of *P. infestans* probably due to the high amino acid sequence similarity of its cellulose synthase enzymes with the ones of *P. viticola*.

## Results

### Identification of peptide aptamers interacting with the *Plasmopara viticola* cellulose synthase 2, *Pv*CesA2

**Figure 1:**
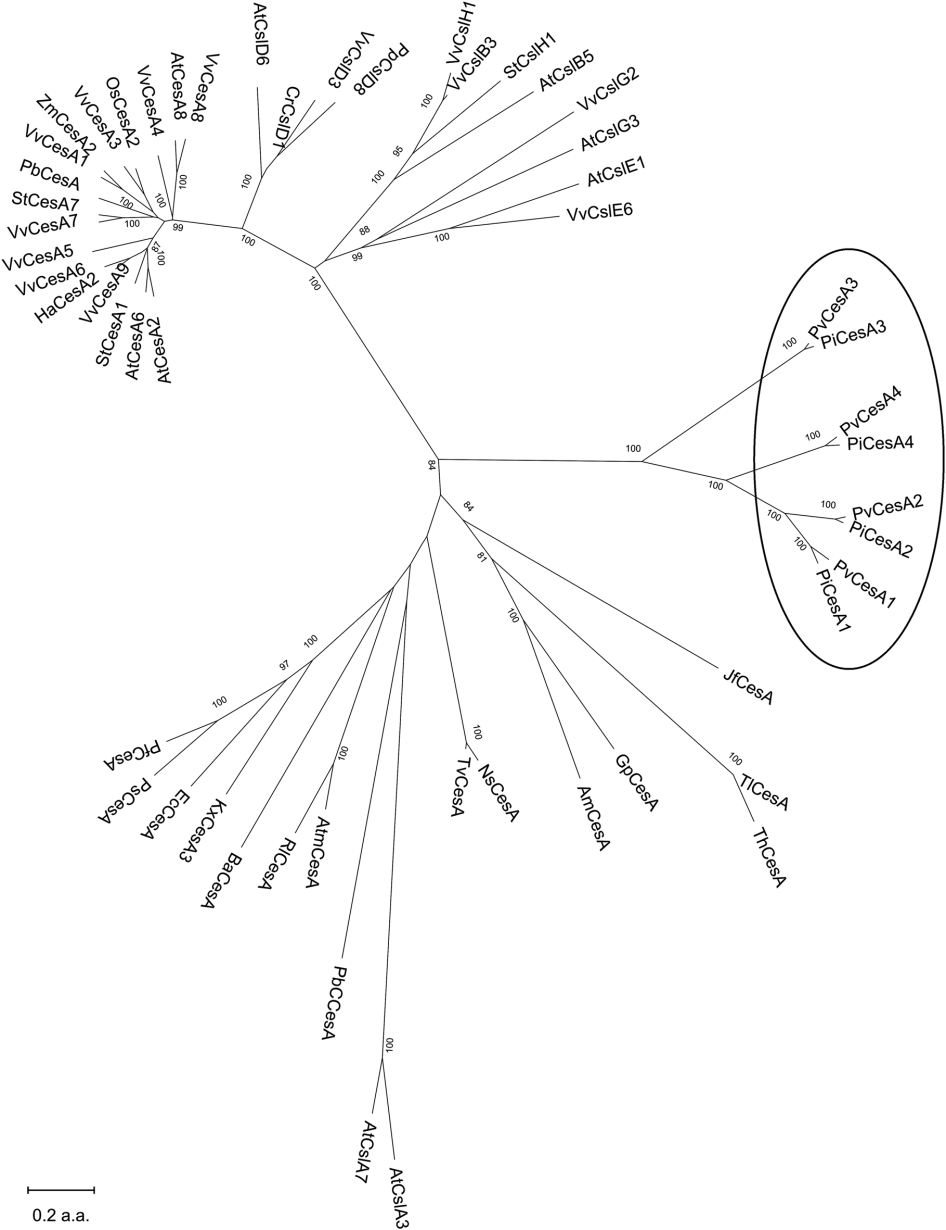
Dendrogram of cellulose synthases derived from Oomycota, Prokarya and Eukarya sequences. Phylogenetic analyses were conducted using MEGA version X^66^ (see Data S1 for Clustal omega multiple sequence alignment). The generated dendrogram demonstrates that Oomycota CesAs group together in a separate clade from all the other considered cellulose synthase and cellulose synthase-like sequences. The analysis is based on complete amino acid sequences reported in Table S1. CesA, cellulose synthase; CslA-H, cellulose synthase-like; Pv, *Plasmopara viticola*; Pi, *Phytophthora infestans*; Ns, *Nostoc* sp.; Tv, *Trichormus variabilis*; At, *Arabidopsis thaliana*; Vv, *Vitis vinifera*; Pb, *Physcomitrella patens*; Pt, *Populus tremula*; Sl, *Solanum tuberosum*; Zm, *Zea mays*; Os, *Oryza sativa*; Atm, *Agrobacterium tumefaciens*; Ec, *Escherichia coli*; Kx, *Komagataeibacter xylinus*; Rl, *Rhizobium leguminosarum*; Ps, *Pseudomonas syringae*; Pf, *Pseudomonas fluorescens*; Ba, *Bacillus amyloliquefaciens*; PbC, *Paramecium bursaria Chlorella* virus 1; Cr, *Ceratopteris richardii*; Pp, *Physcomitrella patens*; Tl, *Trichoderma longibrachiatum*; Th, *Trichoderma harzianum*; Jf, *Jimgerdemannia flammicorona*; Gp, *Gonapodya prolifera*; Am, *Allomyces macrogynus*.

The *Pv*CesA2 cellulose synthase enzyme has been selected as target enzyme for our study since cellulose biosynthesis plays a pivotal role during pathogen infection, as demonstrated by Grenville-Briggs and co-workers^28^ in the closely related oomycete species, *P. infestans*. Furthermore, phylogenetic analysis performed by comparing cellulose synthase amino acid sequences from different organisms (see Fig. 1, Table S1 and Data S1), highlighted that oomycete cellulose synthase proteins group together as a distinct clade, separate from bacterial, cyanobacterial, viral, fungal and plant cellulose synthases^28,30^. In addition, it is also interesting to observe that among the four Oomycota CesAs, CesA3 enzymes cluster in a slightly different clade from CesA1, CesA2 and CesA4, probably due to the lack of a Plekstrin Homology domain (PH domain), involved in regulation, trafficking and/or targeting of polysaccharide synthases to the correct site for cell wall biosynthesis^32^.

These notions, together with the fact that CesA2 is the most abundant among the PH domain-containing CesA enzymes^28^, and that cellulose synthase enzymes are absent in many beneficial organisms, such as *Saccharomyces cerevisiae*, and in animal cells, including human cells, make *Pv*CesA2 the ideal target for developing antimicrobial active compounds.

Peptide aptamers able to specifically interact with *Pv*CesA2 were identified by using the yeast two-hybrid strategy. The cytoplasmic soluble portion of *Pv*CesA2 (from aa 331 to 790; see also Data S1), containing all typical signatures of most processive glycosyltransferases, including three aspartic acid residues and the QXXRW motif^28,33^, was fused to the GAL4 DNA Binding Domain (BD) and used as bait to screen a combinatorial library of linear peptide aptamers (8 a.a. in length; see also “Materials and methods” for further details). Ten small scale transformations were performed (⁓ 10^6^ transformants obtained) and only 30 colonies were able to grow on selective media, lacking either adenine or histidine, and supplemented with 10 mM of 3-amino-1,2,4-triazole (3-AT), a competitive inhibitor for the *HIS*3 reporter gene. To identify the peptide aptamers interacting with *Pv*CesA2, plasmids were purified and sequenced, and priority was given to peptides whose sequences appeared at least three times among the 30 colonies isolated. In total three peptides, named “No *Plasmopora viticola* 1”, − 2 and − 3 (NoPv1, NoPv2 and NoPv3; see also Table 1), were prepared via Microwave-assisted Solid Phase Peptide Synthesis (see “Materials and methods” for further details) and analysed for their capability to counteract *P. viticola* infection of grapevine leaf disks through co-inoculation assays.

**Table 1:** List of peptide aptamers identified by yeast two-hybrid assay using *Pv*CesA2 as bait and mutated derivatives of NoPv1.

Aptamer name	Nucleotide sequence	Peptide sequence
NoPv1	5′-CGTCTGACGGCGCAGTGTCGTCTT-3’	NH_2_-RLTAQCRL-COOH
NoPv2	5′-CTTTTTCCTTTTGTGTCTTCTATG-3’	NH_2_-LFPFVSSM-COOH
NoPv3	5′-ATGTTGCTTCATTCGGAGCTTTGT-3’	NH_2_-MLLHSELC-COOH
NoPv1-R1A	5′- GCGCTGACGGCGCAGTGTCGTCTT-3’	NH_2_-ALTAQCRL-COOH
NoPv1-R7A	5′- CGTCTGACGGCGCAGTGTGCGCTT-3’	NH_2_-RLTAQCAL-COOH
NoPv1-R1A-R7A	5′- GCGCTGACGGCGCAGTGTGCGCTT-3’	NH_2_-ALTAQCAL-COOH

Nucleotide and amino acid sequences (8 a.a.) are provided. Note that sequences of peptides identified at least three times among the 30 colonies isolated are provided.

**Figure 2:**
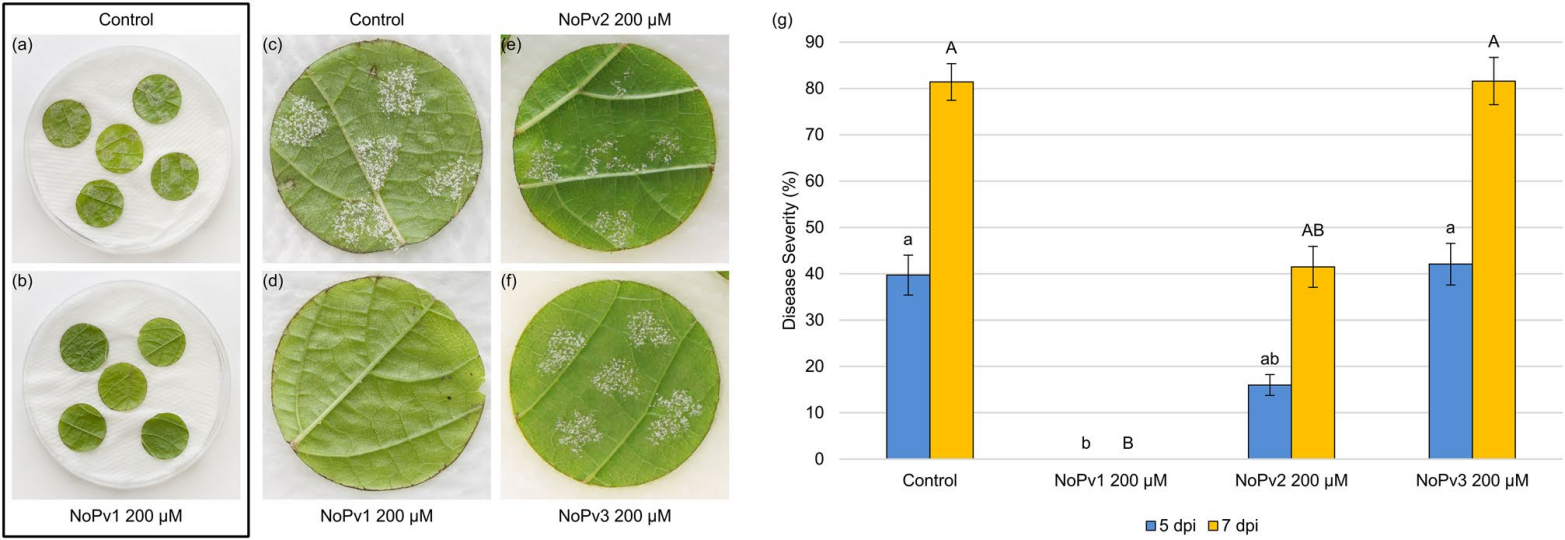
Images of Pinot noir leaf disks co-inoculated ex vivo with *Plasmopara viticola* sporangia in the presence/absence of NoPv peptide aptamers. In the rectangle (a-b) is displayed the experimental setup: 5 leaf disks for each Petri dish and 5 droplets for each leaf disk of *P. viticola* sporangia suspension in the presence/absence of NoPv peptides. (**a**) *P. viticola* sporangia suspension was used to infect grapevine leaf disks, as control. (**b**) *P. viticola* sporangia suspension mixed with NoPv1 peptide (200 μM) was used to inoculate grapevine leaf disks as in (**a**). (**c**) Detail of a single leaf disk shown in (**a**) infected with *P. viticola* sporangia suspension, and used as control. *P. viticola* sporulation can be observed as white spots on leaf surface. (**d**) Detail of a single leaf disk shown in (**b**) infected with *P. viticola* sporangia in the presence of NoPv1 peptide (200 μM). No *P. viticola* sporulation can be observed on leaf disks, i.e. no white spots, pinpointing the anti-oomycete properties of NoPv1. (**e**) Single leaf disk infected with *P. viticola* sporangia in the presence of NoPv2 peptide (200 μM). White spots due to *P. viticola* sporulation are visible, indicating the poor anti-oomycete activity of NoPv2. (**f**) Single leaf disk infected with *P. viticola* sporangia in the presence of NoPv3 peptide (200 μM). White sectors due to *P. viticola* sporulation are visible, indicating the poor anti-oomycete activity of NoPv3. Images were taken at 7 dpi. (**g**) Quantitative evaluation of sporulation of *P. viticola* isolates on leaf disks. Disease severity was assessed at 5 and 7 days post-inoculation (dpi) as percentage of leaf disk area covered by *P. viticola* sporulation, calculated as sum of the disease severity of the five drops for each disk^74^. Each inoculated area was scored as surface with no sporulation (0%), scarce sporulation (10%) or fully covered by sporulation (20%). Each experiment was carried out twice. For each treatment, average and standard error values of ten replicates from the two experiments are shown. Different lowercase and uppercase letters indicate significant differences among treatments at 5 and 7 dpi respectively, according to a Kruskal–Wallis test (P ≤ 0.05).

**Figure 3:**
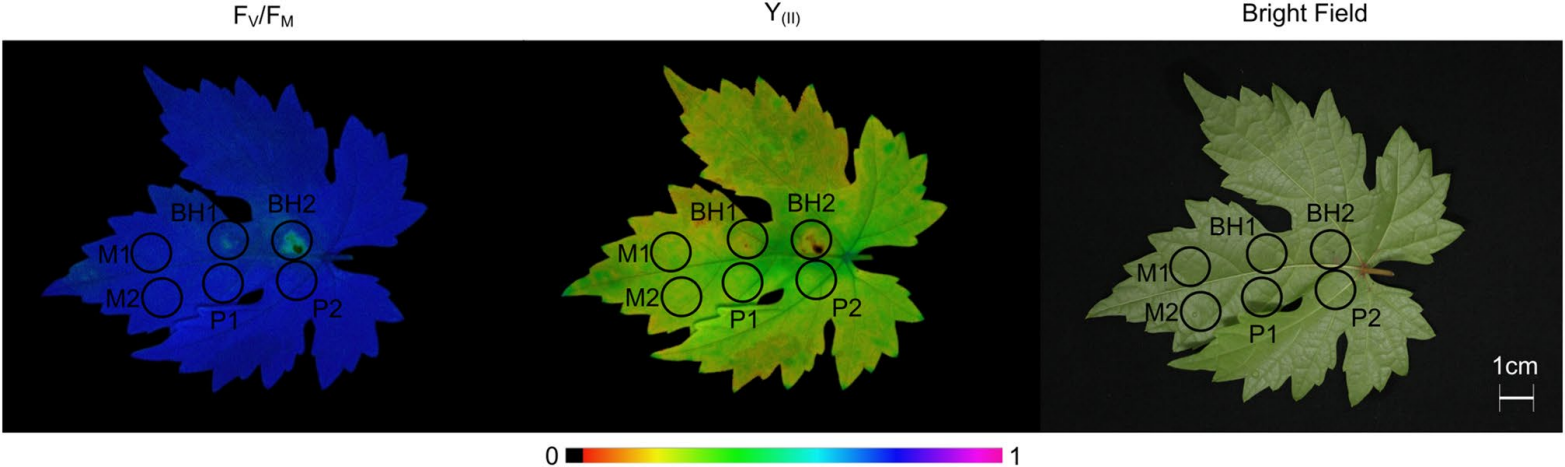
Photosynthetic performance of grapevine leaves exposed ex vivo to 400 µM and 1 mM NoPv1 for 7 days to evaluate NoPv1 cytotoxicity. The maximum quantum yield (F_V_/F_M_) and the effective quantum yield [Y_(II)_] of photosystem II were measured with an imaging Chl fluorometer (Imaging PAM) after 7 days exposure to 400 µM (P1) and 1 mM NoPv1 (P2). As positive control, the same leaves were treated with 0.1% v/v (BH1) and 0.2% v/v (BH2) of BASTA herbicide. Treatment with water (M1 and M2) was used as negative control. Representative images from 10 biological replicates are shown. Herbicide treated spots showed a clear reduction of photosynthetic performance, even though no clear symptoms were visible in bright-field leaf images, highlighting the high sensitivity of the assay. Furthermore, NoPv1- and water-treated spots showed comparable photosynthetic values (see Table S2), indicating that the peptide aptamer is not cytotoxic for plant cells. See Table S2 for average and standard deviation values of photosynthetic parameters. Scale bar = 1 cm.

In details, leaf disks of *V. vinifera* cv. Pinot noir, susceptible to *P. viticola* infection, were treated with sporangia suspension in either absence or presence of 200 μM NoPv1, NoPv2 and NoPv3 (Fig. 2a–f). *P. viticola* sporulation was clearly visible on grapevine leaf disks in the absence of NoPv peptides (Fig. 2a,c,g; Control) at 5 and 7 days post-inoculation (dpi), and NoPv2 and NoPv3 peptide aptamers showed intermediate and no effect on *P. viticola* severity, respectively (Fig. 2e,f,g). On the contrary, 200 μM NoPv1 peptide was able to completely inhibit *P. viticola* severity on leaf disks (Fig. 2b,d,g), without causing any damage to the leaf tissues, as demonstrated by very similar values in the maximum quantum yield of photosystem II (F_V_/F_M_) and effective quantum yield of photosystem II [Y_(II)_] of young developing grapevine leaves, treated or not with 400 μM and 1 mM NoPv1, measured using the IMAGING-PAM (Walz, Germany) to evaluate their photosynthetic performance (Fig. 3 and Table S2).

### Properties of NoPv1

As shown in Table 1, NoPv1 is a peptide of 8 l-amino acid residues with the following sequence: RLTAQCRL. Its molecular weight is of 960.16 Da, the isoelectric point is at pH 10.43, it has a net charge of + 1.9 at pH 7 and a good water solubility (pepcalc.com). Interestingly, the net positive charge has been proposed to be optimal for antimicrobial peptides, since cationic peptides are thought to undertake electrostatic interactions with the negatively charged phospholipid membranes of bacteria, fungi and other microorganisms^34–37^.

**Figure 4:**
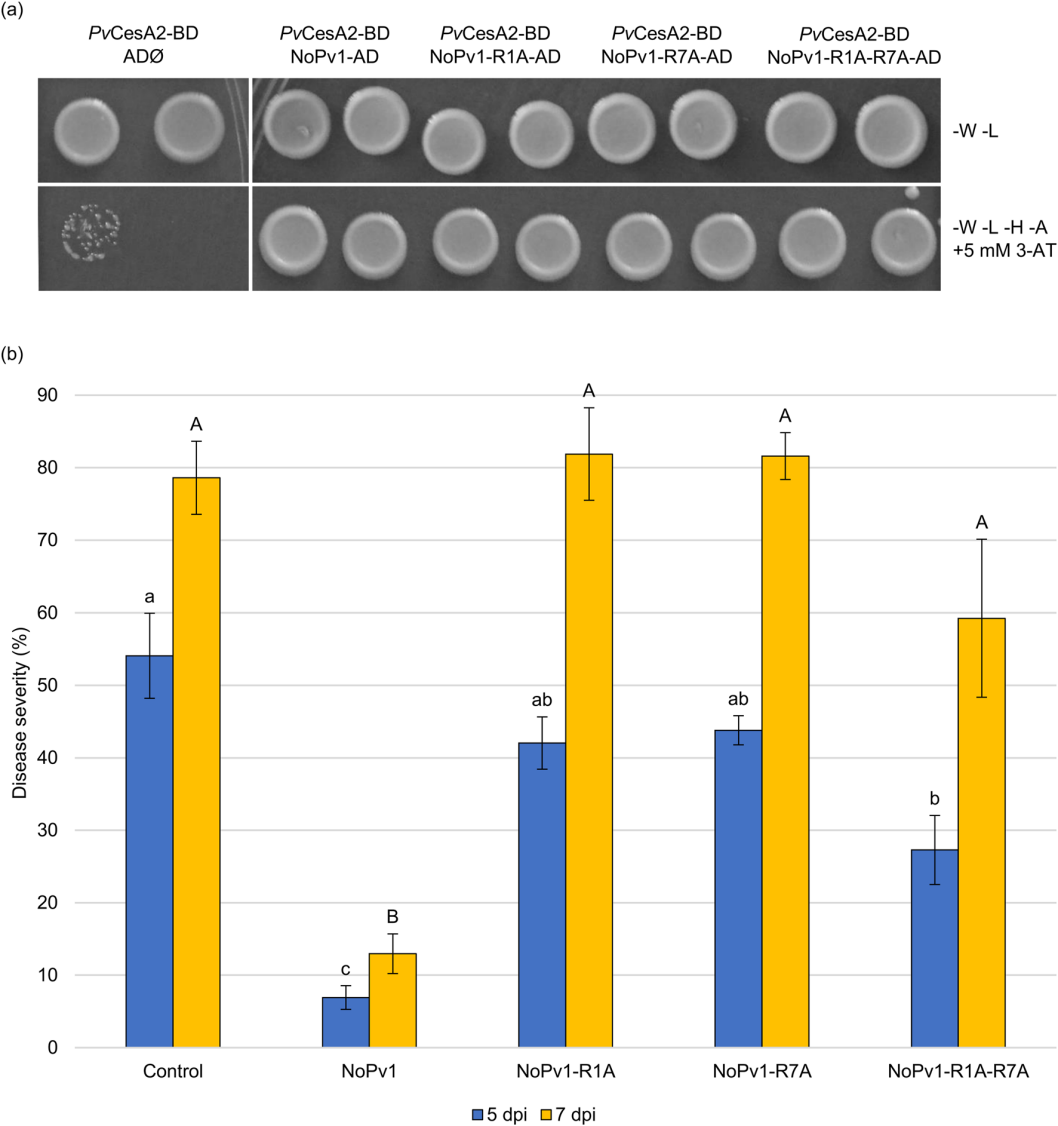
Yeast two-hybrid interaction assay and antimicrobial activity of NoPv1 modified versions. (**a**) NoPv1-R1A, NoPv1-R7A and NoPv1-R1A-R7A were tested for their physical interaction with *Pv*CesA2 enzyme. The unmodified version of NoPv1 was used as positive control in the assay. Interestingly, all the modified versions of NoPv1 interact with the *Pv*CesA2. -W -L, permissive medium devoid of Tryptophan and Leucine; -W -L -H -A, selective medium devoid of Tryptophan, Leucine, Histidine and Alanine supplemented with 5 mM 3-AT (3-Amino-1,2,4-triazole, a histidine biosynthesis inhibitor). (**b**) The anti-oomycete activities of NoPv1-R1A, NoPv1R7A and NoPv1-R1A-R7A (200 μM) were tested by co-inoculating them with *P. viticola* sporangia on grapevine leaf disks and NoPv1 was used as positive control. Disease severity was evaluated at 5 and 7 days post inoculation (dpi). The experiment was carried out twice. For each treatment, average and standard error values of ten replicates from the two experiments are presented. Different lowercase and uppercase letters indicate significant differences among treatments at 5 and 7 dpi respectively, according to a Kruskal–Wallis test (P ≤ 0.05).

To verify the importance of the two Arginine (R) residues for the biological activity of NoPv1, three alanine point mutated derivatives were prepared: NoPv1-R1A (**A**LTAQCRL), NoPv1-R7A (RLTAQC**A**L), and NoPv1-R1A-R7A (**A**LTAQC**A**L) (see also Table 1). Interestingly, all three NoPv1 derivatives were able to interact physically with *Pv*CesA2, as shown by yeast two-hybrid assay (Fig. 4a), but unable to counteract *P. viticola* infection (Fig. 4b), pointing to the fundamental role of the net positive charge of NoPv1 for its biological activity.

### Antimicrobial activity and specificity of NoPv1 peptide aptamer

**Figure 5:**
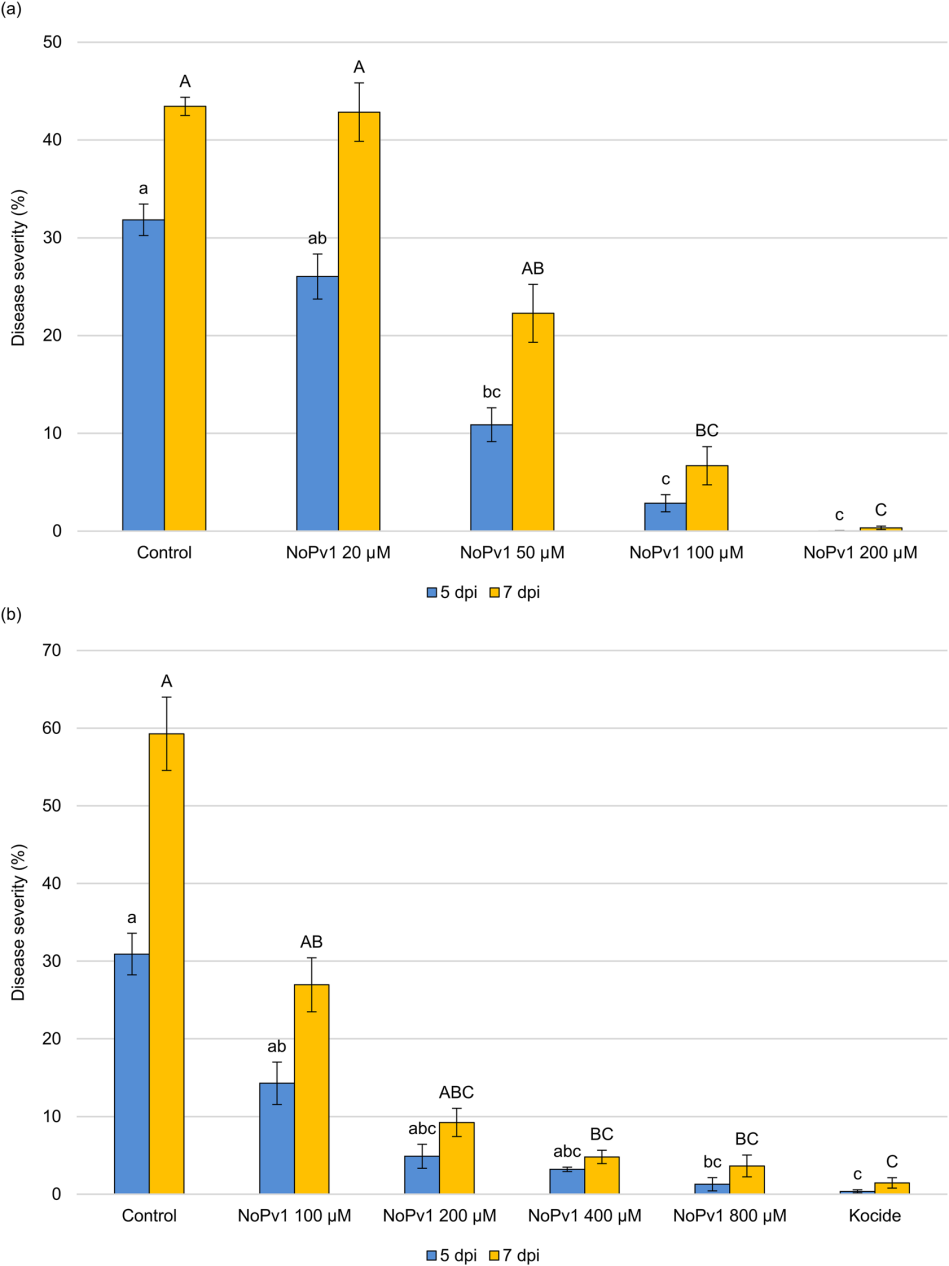
Biological activity of NoPv1 peptide aptamer against *Plasmopara viticola* on leaf disks. (**a**) The activity of NoPv1 peptide solubilised at different concentrations in water (20 μM, 50 μM, 100 μM and 200 μM) and co-inoculated with *P. viticola* sporangia suspension on grapevine leaf disks, as droplets, was monitored at 5 and 7 days post-inoculation (dpi), as percentage of leaf disk area covered by *P. viticola* sporulation, according to the protocol reported in Lazazzara et al.^74^. NoPv1 displayed a good anti-oomycete activity at concentrations equal or higher than 100 μM. (**b**) The anti-oomycete activity of NoPv1 was subsequently evaluated using a Potter Precision Spray Tower, employed to mimic *in field* spray conditions. Leaf disks were treated with NoPv1 at different concentrations, ranging from 100 to 800 μM, and its efficacy, measured as percentage of disease severity, was compared with a copper-based commercial fungicide, (Kocide 2000, copper hydroxide). Each experiment was carried out twice. For each treatment, average and standard error values of ten replicates from the two experiments are presented. Different lowercase and uppercase letters indicate significant differences among treatments at 5 and 7 dpi, respectively, according to a Kruskal–Wallis test (P ≤ 0.05).

**Figure 6:**
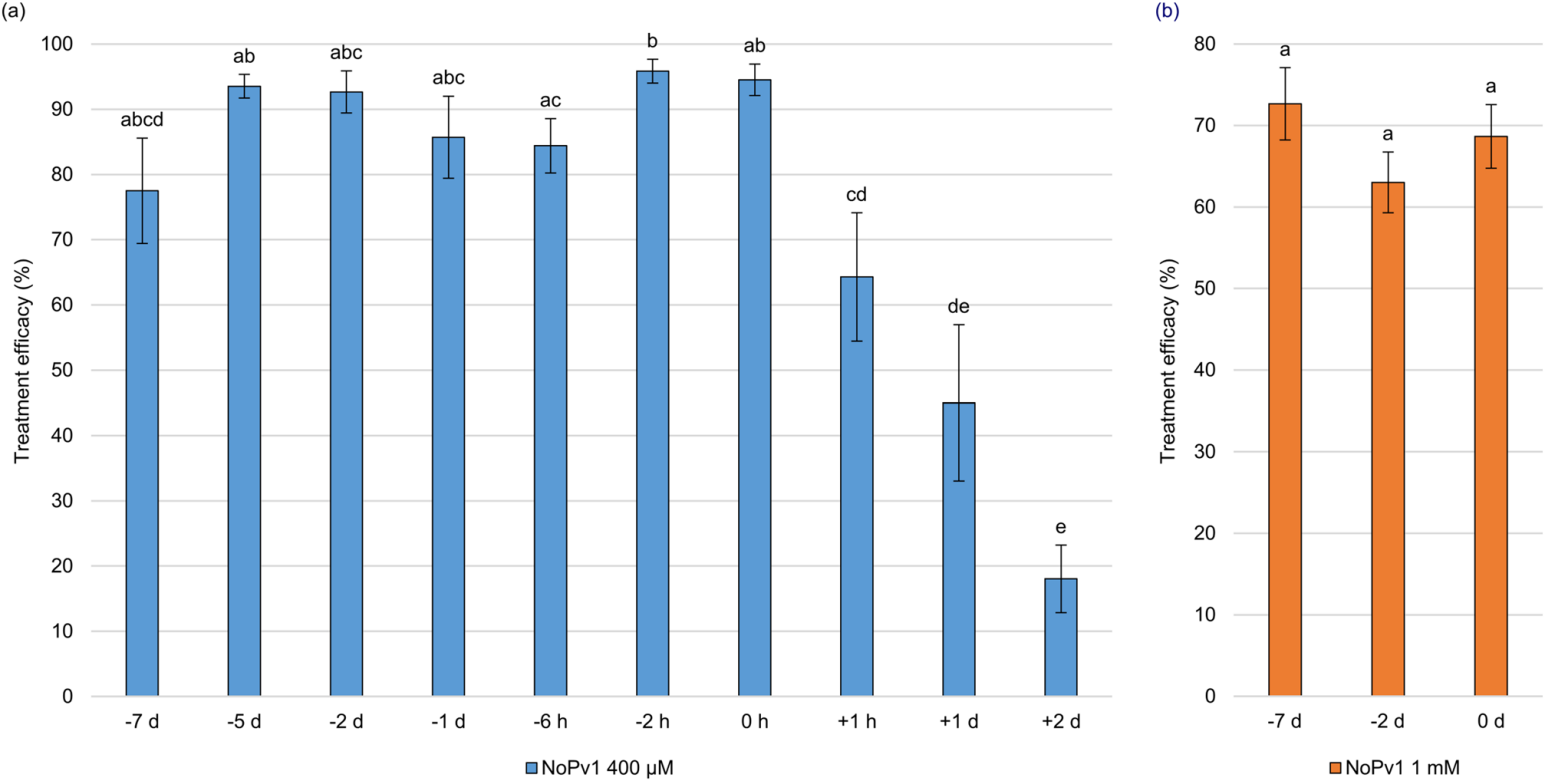
Preventive and curative NoPv1 properties against *Plasmopara viticola* on leaf disks and greenhouse grown plants. (**a**) The anti-oomycete activity of NoPv1 (400 μM) was tested, using the Potter Precision Spray Tower, on leaf disks at different times [days (d)/hours (h)] before (from − 7 d to − 2 h) and after (+ 1 h to + 2 d) *P. viticola* inoculation. The co-inoculation assay (0 h) is also reported in the histogram. Treatment efficacy (see also “Materials and methods”) was evaluated at 7 dpi. Efficacy of NoPv1 was found in preventive treatments, but not in the case of curative treatments. (**b**) Biological activity of NoPv1 peptide on grapevine plants grown under greenhouse conditions. The NoPv1 peptide at 1 mM concentration was sprayed at 7 and 2 days before *P. viticola* inoculation (− 7 d and − 2 d) and co-inoculated (0 h) and the treatment efficacy was assessed at 7 dpi. Preventive treatments with the NoPv1 peptide control *P. viticola* infection under greenhouse conditions. Each experiment was carried out twice. For each treatment, average and standard error values of ten replicates from the two experiments are presented. Different lower case letters indicate significant differences among treatments, according to a Kruskal–Wallis test (P ≤ 0.05).

Properties of NoPv1 against *P. viticola* were further investigated on leaf disks and on potted plants grown in greenhouse. In particular, NoPv1 displayed a good inhibitory activity in the co-inoculation assay on leaf disks at concentrations equal or higher than 100 μM, where the percentage of leaf disk area covered by *P. viticola* sporulation was far below 10% at 5 and 7 dpi (Fig. 5a). On the contrary, lower concentrations were able to contrast only partially *P. viticola* on leaf disks, as disease severity was almost half of the control sample at 5 and 7 dpi in the case of 50 μM NoPv1 and no anti-oomycete activity was observed at 20 μM NoPv1 (Fig. 5a). In addition, nebulization of 400 μM NoPv1 on leaf disks performed using the Potter Precision Spray Tower, recognised as the standard of reference for chemical spraying techniques in the laboratory^38^, indicated a reduction of *P. viticola* severity at 7 dpi (Fig. 5b). Similarly, a solution of 800 μM NoPv1 resulted to be as effective as the copper-based fungicide Kocide 2000 (1.42 g l^−1^ active compound; see also Fig. 5b). Preliminary nebulization tests were also conducted on leaf disks to assess both the preventive and curative action of NoPv1. In particular, 400 μM NoPv1 was the lowest concentration that maximises the efficacy against *P. viticola* (see Fig. 5b) and it was sprayed on leaf disks at different times before [from 7 days (− 7 d) to 2 hours (− 2 h)] and after [from 1 hour (+ 1 h) to 2 days (2 d)] pathogen inoculation (Fig. 6a). The treatment efficacy was evaluated as a measure of the difference of disease severity between control (43.2 ± 4.4%; average ± standard error values) and treated (400 μM NoPv1) samples (see “Materials and methods” for further details) for each time point (Table S3). NoPv1 was effective in suppressing *P. viticola* infection even when applied 7 days before inoculation (-7 d), whereas its efficacy decreased very rapidly when applied after *P. viticola* inoculation, i.e. in post-inoculation treatments (Fig. 6a).

On the basis of the ex vivo results, the activity of NoPv1 against *P. viticola* was tested in vivo on grapevine plants grown under greenhouse conditions. Different NoPv1 concentrations were sprayed on grapevine plants immediately before *P. viticola* inoculation and the disease severity of plants treated with 400 μM (27.3 ± 5.2%), 800 μM (26.2 ± 4.2%), 1 mM (20.8 ± 2.4%) and 2 mM (21.1 ± 4.2) NoPv1 was lower than that of control plants (58.7 ± 6.5%; P < 0.05, Kruskal–Wallis test). In particular, 1 mM NoPv1 was the lowest concentration showing a *P. viticola* severity comparable to the copper treatment (5.4 ± 3.9%; P > 0.05, Kruskal–Wallis test) and it was further used in the following experiments. Therefore, 1 mM NoPv1 was applied at 7 and 2 days before inoculation (− 7 d and − 2 d) and immediately before (0 h) *P. viticola* inoculation to assess the persistence on grapevine plants. As shown in Fig. 6b, the NoPv1 efficacy was always higher than 60% in plants treated with NoPv1 compared to control plants (Fig. 6b), which showed a mean disease severity of 58.15 ± 6.6% (see Table S4).

**Figure 7:**
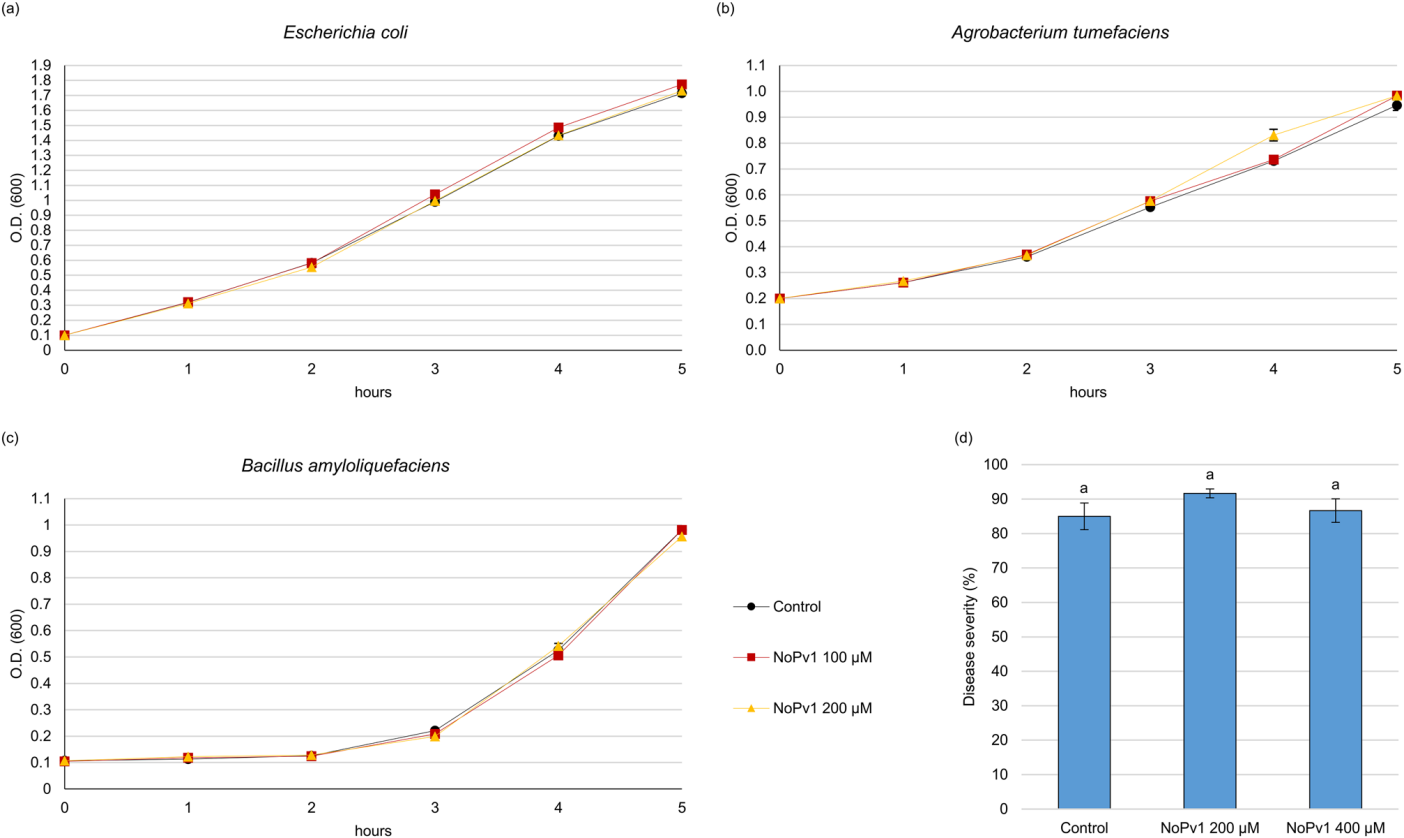
Biological activity of NoPv1 peptide on *Escherichia coli*, *Agrobacterium tumefaciens*, *Bacillus amyloliquefaciens* and *Erysiphe necator.* The NoPv1 peptide was employed to test its effects, at 100 μM and 200 μM, on the growth of *E. coli* (**a**), *Agrobacterium tumefaciens* (**b**) and *Bacillus amyloliquefaciens* (**c**) for 5 h. The growth curves are the average of three biological replicates. (**d**) NoPv1 (200–400 μM) was also co-inoculated with *Erysiphe necator* spores on young grapevine leaves to monitor its antifungal activity. The percentage of disease severity was evaluated at 14 dpi, using the protocol reported in “Materials and methods”. Overall, no inhibitory effects were found. The experiment in (**d**) was carried out twice, average and standard error values of ten replicates from the two experiments are shown. No significant differences were detected (P > 0.05), according to the Kruskal–Wallis test.

**Figure 8:**
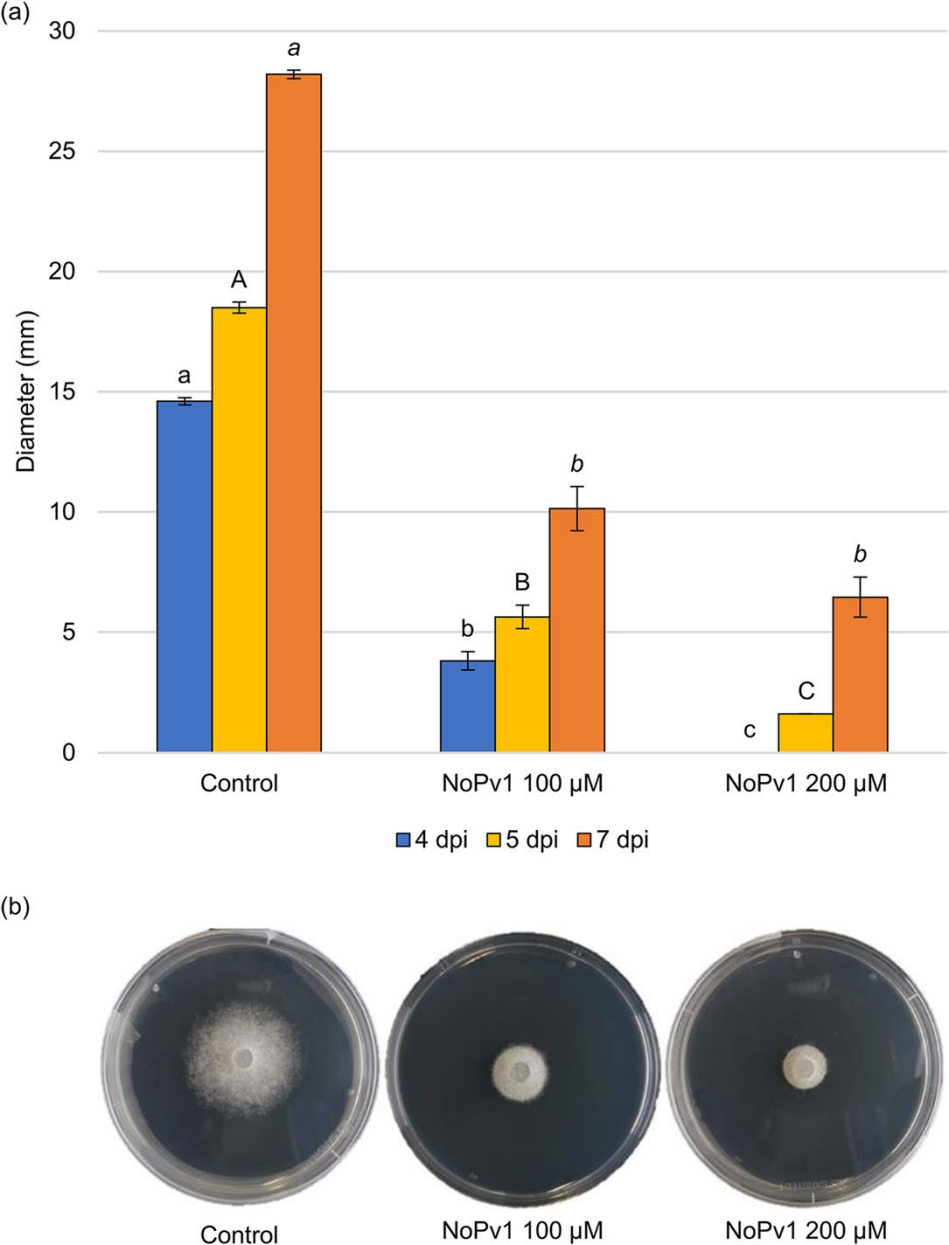
Anti-oomycete activity of NoPv1 on *Phytophthora infestans*. (**a**) The ability of NoPv1 to inhibit the mycelial growth of *P. infestans* at 20 °C was evaluated in vitro by adding 100 μM and 200 μM of peptide to the pea agar medium and measuring the radial growth at 4, 5 and 7 days post inoculation (dpi). (**b**) In representative images of *P. infestans* growth on Petri dishes at 7 dpi, the diameter of *P. infestans* mycelia is smaller in the presence of NoPv1 than under control conditions, indicating that NoPv1 is able to inhibit *P. infestans* growth. Each treatment was carried out on five petri-dishes (replicates), and experiments were carried out twice. Different lowercase, uppercase and italics letters indicate significant differences among treatments at 4, 5 and 7 dpi, respectively, according to a Kruskal–Wallis test (P ≤ 0.05).

NoPv1 specificity was further tested to verify potential harmful effects on non-target organisms. Coherently with the very low homology of CesA enzymes from *Escherichia coli*, *Agrobacterium tumefaciens* and *Bacillus amyloliquefaciens* with *Pv*CesA2 (see also Fig. 1 and Data S1), NoPv1 peptide aptamer, provided directly in the growth medium at 100 μM and 200 μM, was not able to inhibit the growth of these bacteria, as shown by the very similar growth curves (Fig. 7a–c). Furthermore, the co-inoculation of NoPv1 at 200 μM and 400 μM with *Erysiphe necator*, an Ascomycota responsible for grapevine powdery mildew, was not able to reduce the disease severity on leaves (Fig. 7d), in agreement with the fact that the cell wall of *E. necator*, as other Ascomycota, is mainly formed by chitin and other types of glucans^39^, rather than cellulose. On the contrary, comparison of the *Pv*CesA2 cytoplasmic domain used in the yeast two-hybrid screening with the orthologous *P. infestans* CesA2 protein (*Pi*CesA2) showed 97% of amino acid residue identity (Data S1). As it could be hypothesised, NoPv1 was able to inhibit *P. infestans* growth in vitro at the same concentrations used against *P. viticola*, i.e. 100 μM and 200 μM (Fig. 8), however further experiments are needed before concluding that NoPv1 interacts with *Pi*CesA2 and it is able to inhibit cellulose biosynthesis.

### Absence of potential cytotoxicity of NoPv1

**Figure 9:**
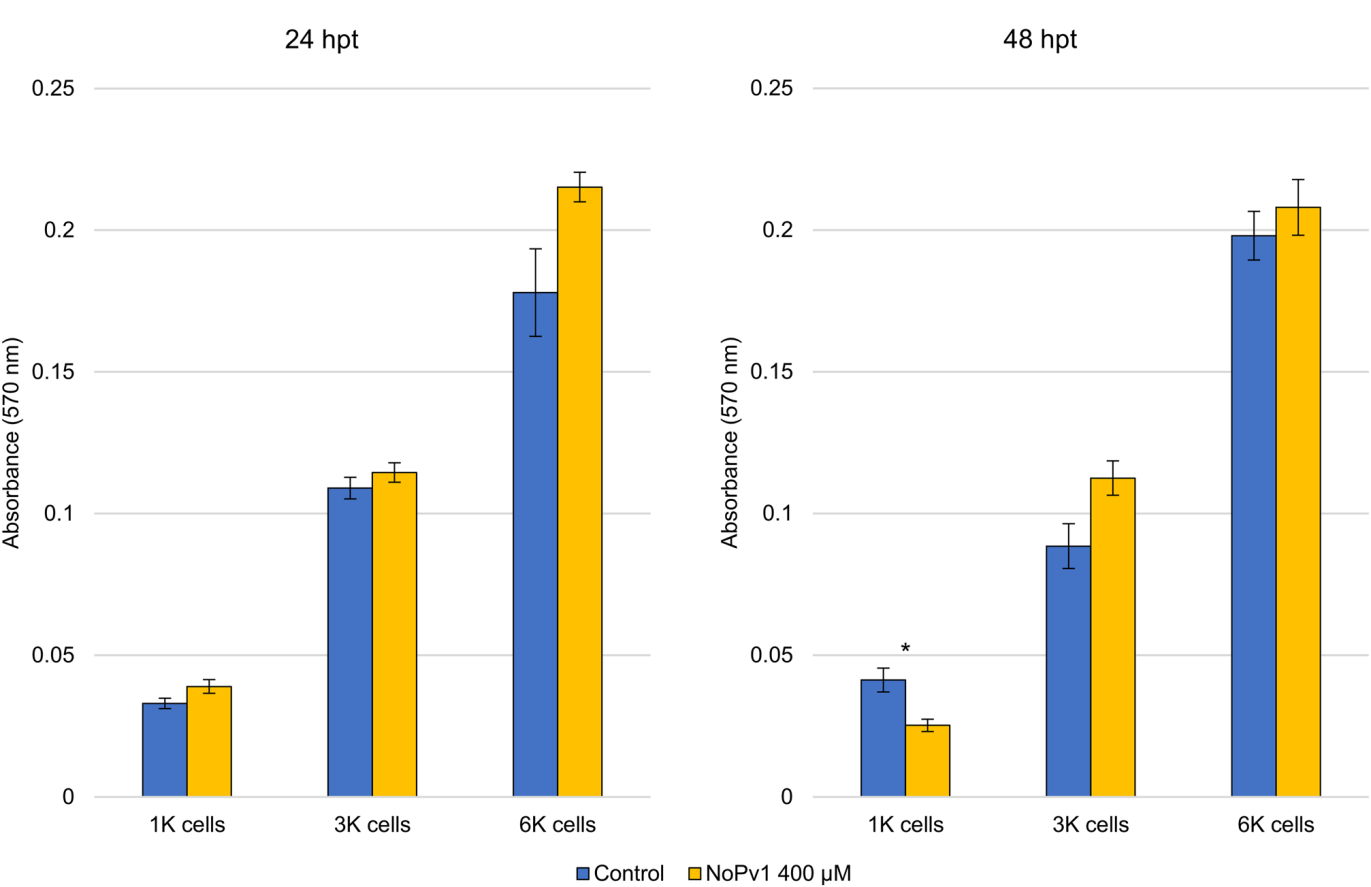
Cell-viability assay to assess the potential cytotoxicity of NoPv1. Immortalized human cells (HKC8), grown in the DMEM-F12 medium at different densities (1 K, 3 K and 6 K cells/100 μl), were grown in the presence of 400 μM NoPv1 and in its absence (Control) for 24 and 48 h. The results demonstrate the absence of any major effect of NoPv1 on the viability of the cells, as shown by the almost identical absorbance levels of MTT-formazan at 570 nm, with the exception of 1 K cell density at 48 h post-treatment (hpt). For each treatment, average and standard error values of four biological replicates are shown. Statistical significance was determined by two-tailed paired Student’s t-test. Each treatment was considered individually and compared with the relative control. *p*-values are represented as *P ≤ 0.05.

To investigate further the potential cytotoxicity of NoPv1, human cells were used to perform an in vitro cell viability test, or MTT assay. Immortalized human cell cultures (HKC8) at different densities (1 K, 3 K and 6 K/100 μl) were grown in DMEM-F12 medium in the presence of 400 μM NoPv1 and their viability was measured after 24 and 48 h from the treatment. No significant differences between control and treated samples were observed after 24 h with respect to the level of absorbance at 570 nm, and cytotoxic effects could only be observed at 1 K cell density after 48 h, indicating that NoPv1 has no major deleterious effects on human cells (see Fig. 9).

### NoPv1 blocks *Plasmopara viticola* germ tube formation

**Figure 10:**
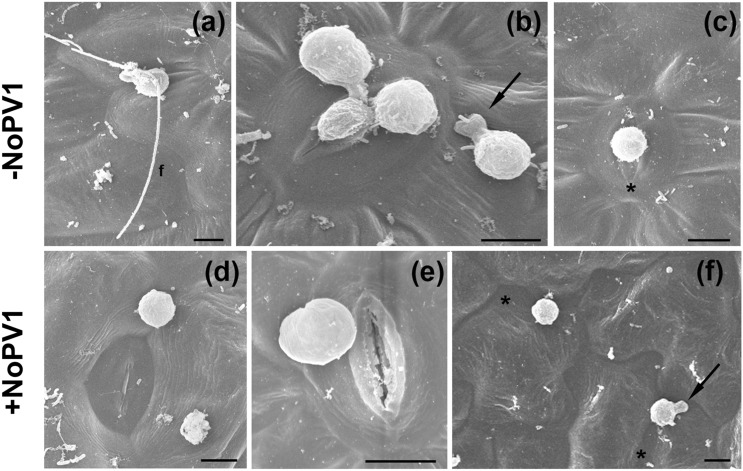
Scanning Electron Microscopy (SEM) images of leaf disks infected with *Plasmopara viticola* zoospores in presence/absence of NoPv1. (**a**–**c**) Grapevine leaf disks infected with a *P. viticola* sporangial suspension (1 × 10^5^ sporangia ml^−1^) were incubated for 6 h in the dark at 22 °C, (f, flagellum). (**d**–**f**) Identical grapevine leaf disks were infected with a *P. viticola* sporangial suspension (1 × 10^5^ sporangia ml^−1^) in presence of 200 μM NoPv1 and incubated for 6 h in the dark at 22 °C. All leaf disks were then fixed, covered with gold and observed with a LEO 1430 scanning electron microscope. NoPv1-treated zoospores were unable to develop a proper germ tube and to penetrate the stomatal cavity of grapevine leaf disks. In (**f**), the black arrow indicates a germ tube primordium that points in the opposite direction with respect to the nearest stomata (asterisk), suggesting that NoPv1-treated zoospores are also unable to sense stomata proximity. In total, about 250 zoospores with and without NoPv1 were observed. The analysis was carried out twice, using 12 independent leaf disks.

Leaf disks of *V. vinifera* cv. Pinot noir were inoculated with *P. viticola* zoospores in the absence (control, Fig. 10a–c) and in the presence (Fig. 10d–f) of 200 μM NoPv1. In control leaf disks, the biflagellate zoospores (n = 90, Fig. 10a) were able to reach the stomata localised on the abaxial leaf surface (Fig. 10b). There, they encysted either alone or in groups (up to 4 germinating cysts/stomata) and emitted a single germ tube (see arrow in Fig. 10b) for each zoospore that penetrated the stomata (Fig. 10b,c). In presence of 200 μM NoPv1, *P. viticola* zoospores were unable to develop any germ tube essential to penetrate the stomata (n = 160, Fig. 10d,e). In the very few cases (5 zoospores out of 160, 3.12%) where a germ tube primordium could be detected (see arrow in Fig. 10f), its orientation was opposite to the stomata localization (see asterisk in Fig. 10f), indicating that NoPv1 prevents germ tube formation and proper stomatal recognition (Fig. 10f).

## Discussion

### The need of alternatives to traditional pesticides

*Plasmopara viticola* is a relevant plant pathogen which requires control measures to avoid severe yield losses. Researchers have dedicated many energies to identify host resistance genes to the pathogen attacks, and a number of breeding programs have introgressed resistance loci from wild North American and Asian *Vitis* species into *V. vinifera* resulting in new downy mildew resistant grapevine cultivars^40,41^. However, most of the European cultivars carry a single major resistance locus named *Rpv3* (Resistance to *P. viticola*), and *P. viticola* isolates able to overcome this resistance have arisen in Europe^42–45^. Because of that and due to the long time required for conventional breeding^46^, wine production is heavily dependent on the use of pesticides to control this disease. Nevertheless, strong selection pressure following repeated pesticide applications has led to the development of resistant pathogen populations, which has limited the success of chemical pesticides in grapevine and other crops^47–50^.

In this scenario, it is important to develop new tools that allow to easily and quickly isolate molecules, intrinsically less dangerous for consumers, farmers and the environment, able to counteract emerging pathogens. Here, we reported the development of an in vivo strategy aimed to isolate synthetic antimicrobial peptide aptamers from a combinatorial library. These synthetic peptide aptamers share several features, including size, level of hydrophobicity and net charge, with the antimicrobial peptides (AMPs), synthesised by the innate immune system of various species including human, animals and plants, which are the first-line defence against foreign attacks^51,52^. This strategy allowed the isolation of the 8 a.a. NoPv1 peptide aptamer able to counteract *P. viticola* and *P. infestans* infections, but it might have a much broader impact in the long term by being applicable to other species and type of crop pathogens, i.e. viruses and bacteria.

### The mechanism of action of NoPv1

NoPv1 inhibits zoospore germ tube formation (see Fig. 10) that requires active cellulose biosynthesis, as reported in *P. infestans* where the cellulose synthases were shown to be localised and play a major role in the growing tip of the germ tube^28^, suggesting a possible inhibitory role of NoPv1 on *Pv*CesA2. This notion seems to be supported by the capability of NoPv1 to affect severely *P. infestans* growth, given that its CesA2 enzyme shares high amino acid sequence identity (97%) with the *Pv*CesA2 (see Fig. 8) and by the failure of NoPv1 to inhibit the growth of bacteria, such as *E. coli*, *A. tumefaciens* and *B. amyloliquefaciens* and to counteract the leaf infection caused by the Ascomycota *E. necator* (see Fig. 7). However, further interaction analyses and in vitro enzymatic assays are needed before concluding that NoPv1 is a specific inhibitor of *Pv*CesA2 and *Pi*CesA2 enzymatic activity. The selectivity of NoPv1 for certain microorganisms might suggest that NoPv1, with two positively charged “R” residues, does not behave as a typical broad spectrum cationic AMP able to alter membrane permeability by simply inserting into the lipid membranes and forming ion channels or pores that eventually result in leakage of cell contents and cell death^53–56^. However, the role of NoPv1 on cell membrane permeabilization remains to be investigated. The replacement of “R” residues with “A” in NoPv1-R1A, NoPv1-R7A and NoPv1-R1A-R7A (see Table 1) abolished the antimicrobial activity (Fig. 4b), but not the physical interaction with *Pv*CesA2 (see Fig. 4a), suggesting that the two cationic residues might be essential for NoPv1 cellular uptake by *P. viticola*, thus reaching the *Pv*CesA2 catalytically active portion, which localizes intracellularly. This is in agreement with the mechanism of action of some cationic AMPs, reported to penetrate cells and affect the cellular physiological processes, without altering the permeability of microbial membranes^57,58^. For instance, the natural echinocadin AMP family targets specifically 1,3 β glucan synthase, an enzyme essential for cell wall integrity of fungi^59^. Furthermore, chitin biosynthesis is blocked by nikkomycins which are the most widely studied peptidyl nucleoside inhibitors of chitin synthases^60,61^. Alternatively, NoPv1 biological activity could strictly rely on the presence of “R” residues without necessarily interacting with the *Pv*CesA2 enzyme.

### The safety and sustainability of NoPv1 potential application under field conditions

Beside target specificity, novel drugs must have a broad-spectrum activity, low toxicity and ideally no off-target organisms. These properties are generally used as guidelines in drug discovery and might give the impression that such molecules are unattainable. The selection of *Pv*CesA2 as a target for peptide aptamers makes NoPv1 both specific for the target and, concomitantly, able to act on a broad spectrum of oomycete pathogens since their cellulose synthases group together in a clade separated from bacteria, viral and plant cellulose synthases (see Fig. 1). As a consequence, NoPv1 is able to inhibit the growth of *P. infestans* (see Fig. 8), an oomycete that causes the serious potato and tomato disease known as late blight or potato blight, but at the same time it does not affect any off-target organism among the ones tested, including plants (see Figs. 3 and 7). Furthermore, the absence of any major cytotoxicity on mammalian cells (see Fig. 9), likely due to the lack of cellulose synthase enzymes and the neutral net charge of cell membranes^35,62^ confers to NoPv1 almost all the desirable features required for an anti-oomycete active compound. Nevertheless, the relatively high concentration of NoPv1 peptide, around 1 mM, shown to have efficient antimicrobial activity under greenhouse conditions seriously challenges its employment under field conditions, mainly as a consequence of the elevated costs of production that are certainly not competitive with the conventional pesticides. However, lower NoPv1 concentrations showed high efficacy on leaf disk assays (e.g. 100 µM and 400 µM in the co-inoculation and nebulization experiment, respectively), suggesting that appropriated formulations could improve NoPv1 stability under field conditions and possibly reduce the risk of degradation by UV light and/or leaf-associated microorganisms. Moreover, a lot can be learnt from the biomedical sector, where recent publications report the successful employment of antifungal peptides in combination with conventional antifungal drugs^63^. Similarly, NoPv1 could be sprayed in combination with conventional active compounds under field conditions, resulting in the reduction of peptide dose, i.e. reduced treatment costs, and in the prevention of drug resistance development as a direct consequence of the mechanistic polyfunctionality improvement of the active compounds. Furthermore, the design of novel delivery systems aimed to improve safety and bioavailability^64^, in combination with precision agriculture tools, may contribute to make the peptide-based pesticides sustainable for the agricultural sector in future.

## Conclusions

We have described a novel approach to identify active compounds to counteract *P. viticola* infection and proposed that this strategy could be applicable to other crop pathogens. In this context, NoPv1 is reported as a highly effective, relatively safe peptide to be used as potential anti-oomycete agent. Despite these features, it must be observed that only a very limited number of antimicrobial peptide-based drugs, around 9, have been approved by the Food and Drug Administration, so far^56^. Based on that, it is reasonable to think that NoPv1 must be subjected to major improvements before its use for agricultural purposes. Obviously, the stability of NoPv1 must be verified at high temperature, in the presence of proteases or at different pH in order to be used under field conditions. However, unfavourable properties limiting peptide usage could be overcome by chemical optimization. For instance, synthetic peptides can be stabilised once chemically modified. Some of them have been reported to keep biological activity at high temperature, and some can resist to hydrolysis mediated by proteases, like tripsin and pepsin^54^. Moreover, the wide variety of structural and functional features identified for various natural antimicrobial peptides represent an invaluable source of ideas for adapting peptides to our needs, providing promising perspectives to the identification of safer alternatives to conventional pesticides. Finally, a cost-efficient peptide manufacturing technology needs to be set-up to compete with the production costs of conventional pesticides. Indeed, the peptide chemical synthesis is not applicable to agriculture purposes due to the high costs of production, however several companies in recent years offer cost-efficient fermentative bioprocesses, up to ton scales, for the production of recombinant peptides.

## Materials and methods

### Phylogenetic and amino acid sequence analysis

CesA and Csl amino acid sequences were obtained from UniprotKB database (uniprot.org), except for KJD55249.1 (BaCesA) which was obtained from the National Center for Biotechnology Information database (ncbi.nlm.nih.gov). Multiple sequence alignment was generated with Clustal omega (ebi.ac.uk/Tools/msa/clustalo)^65^. The dendrogram was constructed with a Minimum-Evolution algorithm and 1000 bootstrap repetitions using MEGA X (megasoftware.net)^66^. Transmembrane regions were predicted with SOSUI (harrier.nagahama-i-bio.ac.jp/sosui)^67^ and domain prediction was performed with CD-BLAST (ncbi.nlm.nih.gov/Structure/cdd/wrpsb.cgi)^68^.

### Plant material and phytopathogens

Two year-old plants of the susceptible *V. vinifera* cv. Pinot noir were grown under greenhouse conditions at 25 ± 1 °C, with a photoperiod of 16 h light and a relative humidity of 70 ± 10%, as described by Perazzolli et al.^69^. A *P. viticola* population was collected in an untreated vineyard sited in the Trentino region (Northern Italy) and maintained by weekly inoculations on potted Pinot noir plants grown in greenhouse as described by Perazzolli et al.^69^. To obtain *P. viticola* inoculum, plants with disease symptoms were incubated overnight in the dark at 99–100% RH and 25 ± 1 °C to promote pathogen sporulation. Sporangia were collected by washing the abaxial surfaces bearing freshly sporulating lesions with cold (4 °C) distilled water and the concentration of the inoculum suspension was adjusted to 1 × 10^5^ sporangia ml^−1^ by counting with a haemocytometer under a light microscope. The *P. infestans* isolate (kindly provided by M. Finckh and A. Butz, University of Kassel, Germany) was grown on pea agar medium (PAM, 12.5% w/v frozen peas in distilled water and 1.2% w/v bacteriological agar) at 20 °C, as reported by Tomada et al.^70^. Infected leaves of greenhouse-grown plants were used as a source of the *E. necator* inoculum.

### RNA extraction and cDNA synthesis

*P. viticola* sporangia were collected by washing the abaxial surfaces of grapevine leaves bearing freshly sporulating lesions with cold (4 °C) distilled water. After centrifugation at 4000×*g* and 4 °C, the pellet (0.05 g) was immediately frozen in N_2_-liquid and stored at − 80 °C. Total RNA was extracted using the Spectrum Plant total RNA kit (Sigma-Aldrich, St. Louis, MO), quantified using the Nanodrop 8000 (Thermo Fisher Scientific, Wilmington, DE) and its quality was checked by agarose gel electrophoresis. RNA was treated with DNase I (Invitrogen, Thermo Fisher Scientific), and first-strand cDNA was synthesised from 500 ng of total RNA using ImProm-II Reverse Transcription System Kit (Promega Corporation, Madison, WI, USA) with a combination of oligo (dT) primers and random hexamers.

### Gene amplification and cloning

The gene fragment encoding the *Pv*CesA2 cytoplasmic portion was amplified from the cDNA template with Phusion High-Fidelity DNA Polymerase (Thermo Fisher Scientific), according to manufacturer’s instruction, using primers GrA007 (5′-GGGGACAAGTTTGTACAAAAAAGCAGGCTTCGACGAGTTTGAGCCGCC-3′) and GrA008 (5′-GGGGACCACTTTGTACAAGAAAGCTGGGTCTACCACTCGGGGTCAAAATATTGG -3′), which contain *attB* sites, and cloned in the Gateway-compatible variant of pGBKT7 (kindly provided by Brendan Davies) passing through pDONR207 (Thermo Fisher Scientific).

NoPv1 mutated derivatives (R residues replaced with A residues) for the two-hybrid experiments were cloned in the following way: the primers GrA_045 (5′-AGTGGATCCAAGCGCTGACGGCGCAGTGTCGTCTTAAGGGGCCCAAAATG-3′) and GrA_046 (5′-CATTTTGGGCCCCTTAAGACGACACTGCGCCGTCAGCGCTTGGATCCACT- 3′) for NoPv1-R1A; GrA_047 (5′-AGTGGATCCAACGTCTGACGGCGCAGTGTGCGCTTAAGGGGCCCAAAATG-3′) and GrA_048 (5′-CATTTTGGGCCCCTTAAGCGCACACTGCGCCGTCAGACGTTGGATCCACT-3′) for NoPv1-R7A; GrA_049 (5′-AGTGGATCCAAGCGCTGACGGCGCAGTGTGCGCTTAAGGGGCCCAAAATG-3′) and GrA_050 (5′-CATTTTGGGCCCCTTAAGCGCACACTGCGCCGTCAGCGCTTGGATCCACT-3′) for NoPv1-R1A-R7A. All primers contain complementary regions and flanking 5′-BamHI and 3′-ApaI restriction sites. After self-annealing, the DNA fragments were digested with BamHI and ApaI restriction enzymes (Fastdigest, Thermo Fisher Scientific), dephosphorylated with FastAP Thermosensitive Alkaline Phosphatase (Thermo Fisher Scientific) and finally ligated into the plasmid pLib2, previously digested with BamHI and ApaI.

### Construction of the peptide aptamer library

The peptide aptamer library was constructed according to Reverdatto et al.^31^ with modifications. The oligonucleotide 5′-GGCAGAGTGGATCCAA(NNK)_8_AAGGGGCCCCTT-3′, which contains complementary regions and flanking 5′-BamHI and 3′-ApaI restriction sites, was self-annealed and the 5′ and 3′ single-stranded ends were filled-in by using the Klenow Fragment (Thermo Fisher Scientific). Subsequently, the DNA fragment was digested with BamHI and ApaI restriction enzymes (Fastdigest, Thermo Fisher Scientific), dephosphorylated with FastAP Thermosensitive Alkaline Phosphatase (Thermo Fisher Scientific) and finally ligated into the plasmid pLib2 (kindly provided by Alexander Shekhtman). The library was then amplified in *E. coli* DH5α competent cells. About 2.5 × 10^6^ colonies were collected with LB medium containing ampicillin and let grow for about 4 h. Plasmids were then purified by using the Qiagen Maxi prep kit.

### Yeast two-hybrid (Y2H) library screening and peptide aptamer identification

The aptamer library was transformed into the yeast strain *Saccharomyces cerevisiae* AH109 (Clontech Laboratories, Palo Alto, CA, USA) harbouring the bait-containing plasmid pGBKT7-*Pv*CesA2^71^. Transformants were selected for growth on selective media without tryptophan, leucine, and either adenine or histidine supplemented with 10 mM 3-AT (3-Amino-1,2,4-triazole, a histidine biosynthesis inhibitor). Yeast plasmid DNA was purified from the positive colonies, electroporated into *E. coli* cells, and the plasmids isolated and sequenced using the GrA_019 primer (5′-TCCAAGCTTTGCAAAGATGG-3′).

### Peptide synthesis and purification

The peptides identified by the Y2H strategy and the NoPv1 mutated derivatives were initially prepared by microwave assisted solid phase synthesis, based on Fmoc chemistry on pre-loaded Wang resin (0.4 meq/g substitution) using a fivefold molar excess of 0.2 M Fmoc-protected amino acids dissolved in NMP and using HOBT/HBTU/DIEA (5:5:10) as activators^72,73^). Coupling reactions were performed for 5 min at 40 W with a maximum temperature of 75 °C. De-protection was performed in two stages using 20% v/v piperidine in DMF (5 min and 10 min each). The cleavage from the resin was performed using 10 ml of Reagent K (TFA/phenol/water/thioanisole/EDT; 82.5/5/5/5/2.5) for 180 min. After cleavage, peptides were precipitated out and washed using ice-cold anhydrous ethyl ether. All peptides were purified by RP-HPLC using a gradient elution of 5–70% v/v solvent B (solvent A: water/acetonitrile/TFA 95/5/0.1; solvent B: water/acetonitrile/TFA 5/95/0.1) over 20 min at a flow rate of 20 ml/min. The purified peptides were freeze-dried and stored at 0 °C. The peptides purity was > 95% and was determined using analytical HPLC (95% v/v A for 5 min; 95–30% v/v A over 20 min) high resolution mass spectrometry (HRMS) and NMR (see Data S2). Peptide identity was also confirmed by electrospray ionization mass spectrometry (ESI–MS). NoPv1 peptide was later purchased at > 95% purity from Bio-Fab Research (Rome, Italy). All the lyophilized peptides and mocks were prepared using the same solvent, either distilled water or 5% v/v DMSO, according to the overall solubility of used peptides.

### Activity of peptide aptamers against *Plasmopara viticola* on leaf disks

Leaves (from the fourth-sixth node) were collected from *V. vinifera* cv. Pinot noir plants grown under greenhouse conditions and surface sterilised by immerging them for 2 min in 1% v/v sodium hypochlorite solution. After that, they were rinsed three times with water and dried on paper. Leaf disks (18 mm diameter) were cut using a cork-borer and placed, abaxial side up, in 9 cm diameter plastic Petri dishes (five disks for each dish), containing four filter papers moistened with 4 ml of sterilised distilled water^42^. Each leaf disk was inoculated with five drops (10 μl) of a *P. viticola* sporangia suspension (1 × 10^5^ sporangia ml^−1^) mixed with the respective peptide aptamer at the appropriate concentration. Dishes were sealed with a plastic film and incubated overnight (16 h) in a growth chamber at 22 ± 1° C in the dark, then dried with filter paper and incubated for 7 days in a growth chamber at 22 ± 1° C and 16 h photoperiod. Disease severity was assessed at 5 and 7 days post inoculation (dpi) as percentage of leaf disk area covered by *P. viticola* sporulation, calculated as sum of the disease severity of the five drops for each disk^74^. Each inoculum drop was scored as surface with no sporulation (0%), scarce sporulation (10%) or fully covered by sporulation (20%). Five replicates (dishes with five disks each) were assessed for each treatment and the experiment was carried out twice.

To analyse the peptide aptamer effects by spray nebulization, Petri dishes (with five leaf disks each) were sprayed using a Potter Precision Spray Tower (Burkard Scientific Co., Uxbridge, UK) with 1.67 ml of the peptide aptamer solution (corresponding to the standard dosage of 10 hl ha^−1^ in a vineyard with a ‘Pergola trentina’ training system) at a pressure of 55 kPa. As control treatments, Petri dishes were sprayed with water (mock) or with copper hydroxide (1.42 g l^−1^ Kocide 2000; Du Pont, Wilmington, DE, USA) as a reference fungicide. Petri dishes were incubated under a laminar flow hood for 5–10 min in order to dry the leaf disks, sprayed with a fresh *P. viticola* sporangia suspension using a small hand sprayer device (0.6 ml per dish). Afterwards, Petri dishes were incubated overnight in the dark at 22 ± 1 °C in a growth chamber, then dried under a laminar flow hood and incubated for 7 days in a growth chamber, as described above. Disease severity was assessed visually as percentage of leaf disk area covered by *P. viticola* sporulation at 5 and 7 dpi^75^. The disease reduction (efficacy) was calculated according to the following formula^76^: (disease severity of mock disks—disease severity of treated disks) / (disease severity of mock disks) × 100. Five replicates (Petri dishes with five leaf disks each) were assessed for each treatment and the experiment was carried out twice. To assess the persistence, as well as the preventive and curative action of the peptide aptamers, treatments were applied on the leaf disks at different times (from 7 days to 2 h) before pathogen inoculation. After each application, leaf disks were dried under a laminar flow hood for 5–10 min and then kept in the growth chamber. In the curative treatment the peptide aptamer was applied after pathogen inoculation (0, 1, 24, 48 h post infection).

### Activity of peptide aptamers against *Plasmopara viticola* on greenhouse-grown plants

*Vitis vinifera* cv. Pinot noir plants were grown in a greenhouse for 2 months as described by Perazzolli et al.^69^. Peptide aptamer was applied with a compressed-air hand sprayer to the abaxial and adaxial surfaces of all leaves (20 to 30 ml, depending on the number of leaves) at 7 days before inoculation, 2 and 0 (co-inoculation), whereas not treated plants were used as control. A fresh sporangia suspension (1 × 10^5^ sporangia ml^−1^) was applied to the abaxial leaf surface using a compressed air hand sprayer (20–30 mL per plant). Inoculated plants were incubated overnight in the dark at 25 ± 1 °C with 99–100% RH. Six days after inoculation, plants were incubated overnight at 25 ± 1 °C with 99–100% RH to promote *P. viticola* sporulation and the disease severity was assessed visually as percentage of abaxial leaf area covered by sporulation in relation to the total leaf area according to EPPO standard guidelines^75^. Five replicates (plants) were assessed for each treatment and time point, and the experiment was carried out twice.

### Antimicrobial activity of peptide aptamers on bacteria

The antibacterial activity of NoPv1 was evaluated in liquid culture using three different bacterial species, *Escherichia coli*, *Agrobacterium tumefaciens* and *Bacillus amyloliquefaciens* (subsp. *plantarum*) strain D 747. Overnight pre-cultures were diluted to OD_600_ of 0.1 (*E. coli*; *B. amyloliquefaciens*) or 0.2 (*A. tumefaciens*) into sterile LB medium. NoPv1 (100 and 200 µM) was then added to the liquid medium of three biological replicates. Bacteria were grown at 28 °C (*A. tumefaciens*) or 37 °C (*E. coli; B. amyloliquefaciens*). The density of the cell population was measured spectrophotometrically at OD_600_ every hour, for 5 h.

### Activity of peptide aptamers against *Phytophthora infestans*

The activity of NoPv1 against *P. infestans* was evaluated on Petri dishes with Pea Agar Medium (PAM) supplemented with 100 µM and 200 µM NoPv1. Mycelium plugs (7 mm in diameter) were cut from 7-day old colonies and placed upside down at the centre of the Petri dish. Petri dishes containing only PAM, without NoPv1, were used as control. Dishes were sealed with a plastic film and incubated in the dark at 20 ± 1 °C. The diameter of *P. infestans* colonies was assessed at 4, 5 and 7 dpi. Five replicates (petri dishes) were analysed for each treatment and the experiment was carried out twice.

### Antifungal activity of peptide aptamers against *Erysiphe necator*

Evaluation of the ability of NoPv1 to protect grapevine plants against *E. necator* was carried out using the method described by Miclot et al.^77^ with minor modifications. The second and third fully expanded leaves from the apex were collected from different *V. vinifera* cv. Pinot noir plants grown under greenhouse condition and surface sterilised by washing for 2 min in a 1% v/v sodium hypochlorite solution. Leaves were rinsed three times for 2 min with distilled water and briefly dried on paper. The base of the petiole was cut off and leaves were placed, adaxial side up, in square culture dishes (120 × 120 mm), containing 1% w/v agar covered with sterile filter paper (1 layer, moistened with 2 ml of sterile distilled water). A small cut was performed with a sterile scalpel on the paper and the petiole was inserted. Four leaves were placed in each culture dish. NoPv1 solution or water (mock) were sprayed on the leaves inside a laminar flow (1–2 ml for each dish) using a small air hand sprayer. Leaves were left to air-dry for 20 min. Pathogen inoculation was performed inside the laminar flow using the dry inoculation method. The open dishes containing the leaves were placed inside a settling tower, a home-made plastic box of 26 × 26 cm base and 42 cm high. Conidia were blown from heavily infected grapevine leaves at the top of the settling tower using an aquarium air pump (Newa Wind NW2 Air Pump, NEWA Tecno Industria, Loreggia, Italy) connected to a Pasteur pipette. Three infected leaves were used as source of inoculum for each round of inoculation. The dishes were then placed in a growth chamber at 22 ± 1 °C, 70% RH and 16 h photoperiod. At 14 dpi, the percentage of disk area covered (% of disease severity) by *E. necator* sporulation was visually assessed, according to the EPPO standard guidelines^78^. Sixteen replicates (leaves) were used for each treatment and the experiment was carried out twice.

### Cell viability and phytotoxicity assays

NoPv1 potential cytotoxicity was tested on SV40-Immortalised human fibroblasts, HKC8, using the MTT assay. This colorimetric assay is dependent on mitochondrial respiration and serves to assess the metabolic activity of a cell. In particular, it measures the activity of the mitochondrial succinate dehydrogenase, active only in living cells, that is capable of reducing MTT (3-[4,5-dimethylthiazol-2-yl]-2,5-diphenyltetrazolium bromide, yellow) to MTT-formazan, a blue/purple substance, the formation of which can be followed by measuring absorption at 570 nm. Therefore, the levels of MTT-formazan and the absorbance at 570 nm are directly proportional to the amount of viable cells present in the sample. Concerning NoPv1 cytotoxicity, HKC8 fibroblasts cultured in DMEM-F12 medium at different cell densities (1 K, 3 K and 6 K cells/100 μl) were exposed to 400 μM NoPv1 for 24 and 48 h.

Potential phytotoxicity of NoPv1 was monitored by estimating the photosynthetic performance of Pinot noir leaves [Maximum quantum yield—F_V_/F_M_—and effective quantum yield—Y_(II)_—of photosystem II] incubated for 7 days with 400 μM and 1 mM NoPv1 at room temperature, using the Imaging PAM (WALZ), as similarly reported in Perreault et al.^79^ and Schreiber et al.^80^. As positive control, 0.1% v/v and 0.2% v/v of BASTA herbicide were used^81^. Mock treatments were performed using water. In total, ten independent leaves were treated and analysed.

### Scanning electron microscopy (SEM) analysis

Leaf disks infected with a *P. viticola* sporangia suspension (1 × 10^5^ sporangia ml^−1^) with and without NoPv1 (200 µM) were fixed for 6 h in 50% v/v ethanol, 5% v/v acetic acid and 3.7% v/v formaldehyde in 0.025 M phosphate buffer, pH 7.0. Samples were collected soon after the infection and at 6 h after the infection. Samples were subsequently washed twice (about 20 min) in 70% v/v ethanol in 0.025 M phosphate buffer, pH 7.0. The material was dehydrated gradually in ethanol series to 100% v/v ethanol, dried in liquid carbon dioxide at the critical point. Samples were subsequently covered with gold using a sputter coater (SEMPREP2; Nanotech, Manchester, UK) and observed with a LEO 1430 scanning electron microscope (LEO Electron Microscopy, leo-usa.com).

### Statistical analysis

Each experiment was carried out at least twice using independent biological samples. Statistical significance of cell viability assay was determined by two-tailed paired Student’s t-test. Different experimental setups were analysed singularly and compared with the relative control. For all the other experiments, data were analysed using the Statistica 13.1 software (TIBCO Software Inc., Tulsa, OK, USA) and a Kruskal–Wallis test was used to demonstrate equivalent results in the two experiments (P > 0.05, non-significant differences between experimental repetitions). Data from the two experimental repetitions were pooled and a Kruskal–Wallis test was then used to detect significant differences among treatments (P ≤ 0.05).

### Patent PCT/IB2018/059834

The amino acid sequence of the NoPv1 peptide and its antimicrobial properties against *Plasmopora viticola* are described in the International Application No. PCT/IB2018/059834; Publication Number WO/2019/116203; Publication Date 20.06.2019.

## Supplementary information

Supplementary Data S1.

Supplementary Data S2.

Supplementary Table S1.

Supplementary Table S2.

Supplementary Table S3.

Supplementary Table S4.
