# Supplementary material for: NoPv1: a synthetic antimicrobial peptide aptamer targeting the causal agents of grapevine downy mildew and potato late blight
Source: Sci Rep. 2020 Oct 16;10:17574. doi: 10.1038/s41598-020-73027-x (PMC7567880; doi:10.1038/s41598-020-73027-x)
Supplement: Supplementary file 1 — Supplementary Data S1. [file 41598_2020_73027_MOESM1_ESM.docx]

**NoPv1: a synthetic antimicrobial peptide aptamer targeting the causal agents of grapevine downy mildew and potato late blight**

Monica Colombo^1+^, Simona Masiero^2+^, Stefano Rosa^2^, Elisabetta Caporali^2^, Silvia Laura Toffolatti^3^, Chiara Mizzotti^2^, Luca Tadini^2^, Fabio Rossi^4^, Sara Pellegrino^5^, Rita Musetti^6^, Riccardo Velasco^7^, Michele Perazzolli^1,8^, Silvia Vezzulli^1*^, Paolo Pesaresi^2*^

^1^ Research and Innovation Centre, Fondazione Edmund Mach, San Michele all'Adige, Italy.

^2^ Department of Biosciences, University of Milan, Milan, Italy.

^3^ Department of Agricultural and Environmental Sciences (DISAA), University of Milan, Milan, Italy.

^4^ Center for Study and Research on Obesity, Department of Medical Biotechnology and Translational Medicine, University of Milan, Milan, Italy.

^5^ DISFARM-Department of Pharmaceutical sciences, University of Milan, Milan, Italy.

^6^ Department of Agricultural, Food, Environmental and Animal Sciences, University of Udine, Udine, Italy

^7^ CREA Research Centre for Viticulture and Enology, Conegliano (TV), Italy

^8^ Centre Agriculture Food Environment (C3A), University of Trento, San Michele all’Adige, Italy

**^+^** These authors contributed equally to the article

** Co-corresponding authors: paolo.pesaresi@unimi.it; silvia.vezzulli@fmach.it*

**Running title:** NoPv1: a low-risk antimicrobial peptide

**Keywords**

*Antimicrobial peptides, Peptide aptamer*, *Pesticide, Phytophthora infestans*, *Plasmopara viticola*, *Vitis vinifera*, *Solanum tuberosum*

**Data S1**. Clustal omega multiple sequence alignment of the full-length cellulose synthase (CesA) and cellulose synthase-like (Csl) amino acid sequences listed in Table S1. The *Pv*CesA2 bait sequence is highlighted in bold. All the typical signatures of most processive glycosyltransferases (three “D” residues and the QXXRW motif) are indicated in red on the bait sequence (Fugelstad *et al.*, 2009; Grenville-Briggs *et al.*, 2008). Black boxes highlight highly conserved amino acids and grey boxes closely related ones.

AtCslD6 1 ------------------------------------------------------------
VvCslD3 1 ------------------------------------------------------------
CrCslD1 1 ------------------------------------------------------------
PpCslD8 1 ------------------------------------------------------------
AtCesA8 1 ------------------------------------------------------------
VvCesA8 1 ------------------------------------------------------------
VvCesA4 1 ------------------------------------------------------------
VvCesA5 1 ------------------------------------------------------------
AtCesA2 1 ------------------------------------------------------------
AtCesA6 1 ------------------------------------------------------------
StCesA1 1 ------------------------------------------------------------
HaCesA2 1 ------------------------------------------------------------
VvCesA6 1 ------------------------------------------------------------
VvCesA9 1 ------------------------------------------------------------
PbCesA 1 ------------------------------------------------------------
VvCesA7 1 ------------------------------------------------------------
StCesA7 1 ------------------------------------------------------------
VvCesA1 1 ------------------------------------------------------------
ZmCesA2 1 ------------------------------------------------------------
VvCesA3 1 ------------------------------------------------------------
OsCesA2 1 ------------------------------------------------------------
AtCslB5 1 ------------------------------------------------------------
StCslH1 1 ------------------------------------------------------------
VvCslH1 1 ------------------------------------------------------------
VvCslB3 1 ------------------------------------------------------------
VvCslG2 1 ------------------------------------------------------------
AtCslG3 1 ------------------------------------------------------------
AtCslE1 1 ------------------------------------------------------------
VvCslE6 1 ------------------------------------------------------------
AtCslA3 1 ------------------------------------------------------------
AtCslA7 1 ------------------------------------------------------------
PbCCesA 1 ------------------------------------------------------------
BaCesA 1 ------------------------------------------------------------
AtmCesA 1 ------------------------------------------------------------
RlCesA 1 ------------------------------------------------------------
KxCesA3 1 ------------------------------------------------------------
EcCesA 1 ------------------------------------------------------------
PsCesA 1 MRSYRMSRADDVANLFQRF---GASSDGYLEI----------------------------
PfCesA 1 ------------------------------------------------------------
PvCesA3 1 ---MGLTGAGVIASVIGILGGVSLSCGGWSSLSLGARSLFVTTQFLSAFAMGFVVAFTAI
PiCesA3 1 ---MGLTGAGIIASVVGILGGVSLSCGGWSSLSLGARSLFVTTQFLSAFAMGFVVAFSAI
PvCesA4 1 ------------------------------------------------------------
PiCesA4 1 ------------------------------------------------------------
PvCesA2 1 ------------------------------------------------------------
PiCesA2 1 ------------------------------------------------------------
PvCesA1 1 ------------------------------------------------------------
PiCesA1 1 ------------------------------------------------------------
NsCesA 1 ------------------------------------------------------------
TvCesA 1 ------------------------------------------------------------
JfCesA 1 ------------------------------------------------------------
TlCesA 1 ------------------------------------------------------------
ThCesA 1 ------------------------------------------------------------
GpCesA 1 ------------------------------------------------------------
AmCesA 1 ------------------------------------------------------------
AtCslD6 1 ------------------------------------------------------------
VvCslD3 1 ------------------------------------------------------------
CrCslD1 1 ------------------------------------------------------------
PpCslD8 1 ------------------------------------------------------------
AtCesA8 1 ------------------------------------------------------------
VvCesA8 1 ------------------------------------------------------------
VvCesA4 1 ------------------------------------------------------------
VvCesA5 1 ------------------------------------------------------------
AtCesA2 1 ------------------------------------------------------------
AtCesA6 1 ------------------------------------------------------------
StCesA1 1 ------------------------------------------------------------
HaCesA2 1 ------------------------------------------------------------
VvCesA6 1 ------------------------------------------------------------
VvCesA9 1 ------------------------------------------------------------
PbCesA 1 ------------------------------------------------------------
VvCesA7 1 ------------------------------------------------------------
StCesA7 1 ------------------------------------------------------------
VvCesA1 1 ------------------------------------------------------------
ZmCesA2 1 ------------------------------------------------------------
VvCesA3 1 ------------------------------------------------------------
OsCesA2 1 ------------------------------------------------------------
AtCslB5 1 ------------------------------------------------------------
StCslH1 1 ------------------------------------------------------------
VvCslH1 1 ------------------------------------------------------------
VvCslB3 1 ------------------------------------------------------------
VvCslG2 1 ------------------------------------------------------------
AtCslG3 1 ------------------------------------------------------------
AtCslE1 1 ------------------------------------------------------------
VvCslE6 1 ------------------------------------------------------------
AtCslA3 1 ------------------------------------------------------------
AtCslA7 1 ------------------------------------------------------------
PbCCesA 1 ------------------------------------------------------------
BaCesA 1 ------------------------------------------------------------
AtmCesA 1 ------------------------------------------------------------
RlCesA 1 ------------------------------------------------------------
KxCesA3 1 ------------------------------------------------------------
EcCesA 1 ------------------------------------------------------------
PsCesA 30 ---DNSLDYQESSVSRTPSAALQRATSQPSLEQ-----------------------PCST
PfCesA 1 ------------------------------------------------------------
PvCesA3 58 VSLSDTNEWVAVIAGGGAGFVIALIVGFLTFFGPYILILITGGIIASYLLLIDAFNGINV
PiCesA3 58 VSLSDTNEWVAVAAGGGAGFVIALIGGFMTIFGPYILILITGGLIACYLLLVDAYDGINV
PvCesA4 1 ------------------------------------------------------------
PiCesA4 1 ------------------------------------------------------------
PvCesA2 1 ------------------------------------------------------------
PiCesA2 1 ------------------------------------------------------------
PvCesA1 1 ------------------------------------------------------------
PiCesA1 1 ------------------------------------------------------------
NsCesA 1 ------------------------------------------------------------
TvCesA 1 ------------------------------------------------------------
JfCesA 1 ------------------------------------------------------------
TlCesA 1 ------------------------------------------------------------
ThCesA 1 ------------------------------------------------------------
GpCesA 1 ------------------------------------------------------------
AmCesA 1 ------------------------------------------------------------
AtCslD6 1 ---------------------------------------MMDG--------------ESP
VvCslD3 1 ----------------------------------------MGSRFFNPKNPSGSVNSGEH
CrCslD1 1 ------------------------------------------------------------
PpCslD8 1 ------MASPRSAA---TGGLGF-------RNAGQSRGAQMGGRSN-PDS--HSSNSGPS
AtCesA8 1 ------------------------------------------------------------
VvCesA8 1 ------------------------------------------------------------
VvCesA4 1 ------------------------------------------------------------
VvCesA5 1 ------------------------------------------------------------
AtCesA2 1 ------------------------------------------------------------
AtCesA6 1 ------------------------------------------------------------
StCesA1 1 ------------------------------------------------------------
HaCesA2 1 ------------------------------------------------------------
VvCesA6 1 ------------------------------------------------------------
VvCesA9 1 ------------------------------------------------------------
PbCesA 1 ------------------------------------------------------------
VvCesA7 1 ---------------------------------------MVRT-----------------
StCesA7 1 ------------------------------------------------------------
VvCesA1 1 ------------------------------------------------------------
ZmCesA2 1 ------------------------------------------------------------
VvCesA3 1 ------------------------------------------------------------
OsCesA2 1 ------------------------------------------------------------
AtCslB5 1 ------------------------------------------------------------
StCslH1 1 ------------------------------------------------------------
VvCslH1 1 ------------------------------------------------------------
VvCslB3 1 ------------------------------------------------------------
VvCslG2 1 ------------------------------------------------------------
AtCslG3 1 ------------------------------------------------------------
AtCslE1 1 ------------------------------------------------------------
VvCslE6 1 ------------------------------------------------------------
AtCslA3 1 ------------------------------------------------------------
AtCslA7 1 ------------------------------------------------------------
PbCCesA 1 ------------------------------------------------------------
BaCesA 1 ------------------------------------------------------------
AtmCesA 1 ------------------------------------------------------------
RlCesA 1 ------------------------------------------------------------
KxCesA3 1 ------------------------------------------------------------
EcCesA 1 ------------------------------------------------------------
PsCesA 64 LPAQNNSTAPDLIAPDKTRRIAADTLTKLLAEAAQARQAEAQARNN--EALAQSMGKGQL
PfCesA 1 ------------------------------------------------------------
PvCesA3 118 FPADNQLARQEFV-------IAFMIIFELVCSST-SKTS-------------------EL
PiCesA3 118 FPADNQLARQEFV-------IAFMIIFELVCCSS-SKTS-------------------EL
PvCesA4 1 ------------------------------------------------------------
PiCesA4 1 ------------------------------------------------------------
PvCesA2 1 ------------------------------------------------------------
PiCesA2 1 ------------------------------------------------------------
PvCesA1 1 ------------------------------------------------------------
PiCesA1 1 ------------------------------------------------------------
NsCesA 1 ------------------------------------------------------------
TvCesA 1 ------------------------------------------------------------
JfCesA 1 ------------------------------------------------------------
TlCesA 1 ------------------------------------------------------------
ThCesA 1 ------------------------------------------------------------
GpCesA 1 ------------------------------------------------------------
AmCesA 1 ------------------------------------------------------------
AtCslD6 8 LRHP------RISHVSNSGS-----DFGS-------SSDYNKYL---------VQ-IPPT
VvCslD3 21 I------------ELNSSSCTYDLDSEAG-------SGEYATYT---------VH-LPPT
CrCslD1 1 ------------------------------------------------------------
PpCslD8 42 LHHARRTSSGRFNNLSRDMSEMGGVTDSE-------LGSDYLYT---------VQ-IPAT
AtCesA8 1 ------------------------------------------------------------
VvCesA8 1 ------------------------------------------------------------
VvCesA4 1 ------------------------------------------------------------
VvCesA5 1 ------------------------------------------------------------
AtCesA2 1 ------------------------------------------------------------
AtCesA6 1 ------------------------------------------------------------
StCesA1 1 ------------------------------------------------------------
HaCesA2 1 ------------------------------------------------------------
VvCesA6 1 ------------------------------------------------------------
VvCesA9 1 ------------------------------------------------------------
PbCesA 1 ------------------------------------------------------------
VvCesA7 5 ------------------------KDFSP-------HPS--PFL---------FHKTPPS
StCesA7 1 ------------------------------------------------------------
VvCesA1 1 ------------------------------------------------------------
ZmCesA2 1 ------------------------------------------------------------
VvCesA3 1 ------------------------------------------------------------
OsCesA2 1 ------------------------------------------------------------
AtCslB5 1 ------------------------------------------------------------
StCslH1 1 ------------------------------------------------------------
VvCslH1 1 ------------------------------------------------------------
VvCslB3 1 ------------------------------------------------------------
VvCslG2 1 ------------------------------------------------------------
AtCslG3 1 ------------------------------------------------------------
AtCslE1 1 ------------------------------------------------------------
VvCslE6 1 ------------------------------------------------------------
AtCslA3 1 ------------------------------------------------------------
AtCslA7 1 ------------------------------------------------------------
PbCCesA 1 ------------------------------------------------------------
BaCesA 1 ------------------------------------------------------------
AtmCesA 1 ------------------------------------------------------------
RlCesA 1 ------------------------------------------------------------
KxCesA3 1 ------------------------------------------------------------
EcCesA 1 ------------------------------------------------------------
PsCesA 122 SRTPAH----VIAVVSAKGGV-GKSTLSAALTSLVKVPGGQTLAIDLDPQNALQHHLNAS
PfCesA 1 ------------------------------------------------------------
PvCesA3 151 ENHRFK----YIIFSCITGGWMAADGLSRLIDSSAVL---STVA-----FDSIQDGGKAA
PiCesA3 151 ENHRFK----YIVFSAITGGWMAADGVSRLIDSGAVL---STVA-----YTSIQDGGKAA
PvCesA4 1 ------------------------------------------------------------
PiCesA4 1 ------------------------------------------------------------
PvCesA2 1 ------------------------------------------------------------
PiCesA2 1 ------------------------------------------------------------
PvCesA1 1 ------------------------------------------------------------
PiCesA1 1 ------------------------------------------------------------
NsCesA 1 ------------------------------------------------------------
TvCesA 1 ------------------------------------------------------------
JfCesA 1 ------------------------------------------------------------
TlCesA 1 ------------------------------------------------------------
ThCesA 1 ------------------------------------------------------------
GpCesA 1 ------------------------------------------------------------
AmCesA 1 ------------------------------------------------------------
AtCslD6 40 PDNNPGPAS---------------------------------------------------
VvCslD3 52 PDNRPSGLDIQLDGRVSQRVEEHYTANSIFTGGHNSVTR---AHLMDKVTESEASHPQMA
CrCslD1 1 ------------------------------------------------------------
PpCslD8 85 PDHPMAGDR-----VIPGKAQQQFVSSTIFTGGFSNQTR---GHTMEKMMEDQGNHPQLG
AtCesA8 1 ----------------------------------------------------------MM
VvCesA8 1 ----------------------------------------------------------MM
VvCesA4 1 --------------------MASNTMAGLVAGSHT-RNE---MHVLHGEQRP-----PTR
VvCesA5 1 ----------------------MEASAGLVAGSHN-RNE---LVVIRRDGES--GPKA--
AtCesA2 1 ----------------------MNTGGRLIAGSHN-RNE---FVLINADESA--RIRSVQ
AtCesA6 1 ----------------------MNTGGRLIAGSHN-RNE---FVLINADENA--RIRSVQ
StCesA1 1 ------------------------------------------------------------
HaCesA2 1 ----------------------METKGRLIAGSHN-RNE---FVLINADEIA--RVTSVK
VvCesA6 1 ----------------------MDTKGRLVAGSHN-RNE---FVLINADEIG--RVTSVK
VvCesA9 1 ------------------------------------------------------------
PbCesA 1 ----------------------MEANAGLVAGSHN-RNE---LVIIRQDGD---GPKPLN
VvCesA7 23 LNIPPS-LPLVFT----SPLSTMEASAGLVAGSHN-RNE---LVVIHG-HE---EPKPLR
StCesA7 1 ----------------------MEAGAGLVAGSHN-RNE---LVVIHG-HE---EHKPLK
VvCesA1 1 ----------------------MEANAGMVAGSHK-RNE---LVRIRHDSDS--GPKPLK
ZmCesA2 1 ----------------------MAANKGMVAGSHN-RNE---FVMIRHDGDAPVPAKPTK
VvCesA3 1 -----------------------------------------------MDSEGESGAKSLK
OsCesA2 1 ----------------------------------------------------MDG--AKS
AtCslB5 1 ------------------------------------------------------------
StCslH1 1 ------------------------------------------------------------
VvCslH1 1 ------------------------------------------------------------
VvCslB3 1 ------------------------------------------------------------
VvCslG2 1 ------------------------------------------------------------
AtCslG3 1 ------------------------------------------------------------
AtCslE1 1 ------------------------------------------------------------
VvCslE6 1 ------------------------------------------------------------
AtCslA3 1 ------------------------------------------------------------
AtCslA7 1 ------------------------------------------------------------
PbCCesA 1 ------------------------------------------------------------
BaCesA 1 ------------------------------------------------------------
AtmCesA 1 ------------------------------------------------------------
RlCesA 1 ------------------------------------------------------------
KxCesA3 1 ------------------------------------------------------------
EcCesA 1 ------------------------------------------------------------
PsCesA 177 PDVA---------GLGGASLSGENWRALLLSGSADTQLLAYGALQLDERRS---------
PfCesA 1 ------------------------------------------------------------
PvCesA3 199 LKGI---------DAGGQSLMFLLWAAVVVIGGLNQLSMRWGLLCYNRVGAHAQLGPVEE
PiCesA3 199 MDGI---------DASAQTLMFVIWGAVFVVGGLNQLSMRWGLMCYNRVGAHAQLGPVEE
PvCesA4 1 --------------------------------------------MTNRDAPRPSALPEDA
PiCesA4 1 --------------------------------------------MANRQPPGLGALPEDA
PvCesA2 1 --------------------------------------------MFGNDKQL---LIKHE
PiCesA2 1 --------------------------------------------MYGNDKQS---LMKHE
PvCesA1 1 --------------------------------------------MSKKDKTAA-GMPPTD
PiCesA1 1 --------------------------------------------MFNKDQTAT-AVPPTD
NsCesA 1 ------------------------------------------------------------
TvCesA 1 ------------------------------------------------------------
JfCesA 1 ------------------------------------------------------------
TlCesA 1 ------------------------------------------------------------
ThCesA 1 -------------------------------------------MMR----------GHQE
GpCesA 1 ------------------------------------------------------------
AmCesA 1 ------------------------------------------------------------
AtCslD6 49 ------------------------------------------------------------
VvCslD3 109 GSKGSTCAI----PG-CDAKIMTDERGEDILPC-ECDFKICRDCYVDAVR------TGDG
CrCslD1 1 ------------------------------------------------------------
PpCslD8 137 AVRGPTCSV----IN-CDGKAMRDERGEDMTPC-DCHFKICRDCYIDAL-------NGSG
AtCesA8 3 ESRSPICNT-------CGEEIGVKSNGEFFVACHECSFPICKACLEYEFK------EGRR
VvCesA8 3 QSGVPPCTT-------CGEPVGFDSNGEVFVACHECNFPVCKSCLDYEIK------EGRK
VvCesA4 32 QSVPKLCRV-------CGDEIGVKADGELFVACHECGFPVCKPCYEYERS------EGNQ
VvCesA5 31 ------------------------------VAAAEWTNLPDITCYEYERR------EGSQ
AtCesA2 33 ELSGQTCQI-------CGDEIELTVSSELFVACNECAFPVCRPCYEYERR------EGNQ
AtCesA6 33 ELSGQTCQI-------CRDEIELTVDGEPFVACNECAFPVCRPCYEYERR------EGNQ
StCesA1 1 ------------------------------------------------------------
HaCesA2 33 ELSGQICKI-------CGDEIEVTVDGEPFVACNECAFPVCRPCYEYERR------EGNQ
VvCesA6 33 ELSGQICQI-------CGDEIEITVDGEPFVACNECAFPVCRPCYEYERR------EGNQ
VvCesA9 1 ------------------------------------------------------------
PbCesA 32 NVNSHICQI-------CGDDVGITTEGELFVACNECGFPVCRPCYEYERH------EGNQ
VvCesA7 70 SLNGQVCEI-------CGDEIGLTVDGEVFVACNECGFPVCRPCYEYERR------EGSQ
StCesA7 31 DLSGQVCDI-------CGDEIGLTVDGDLFVACNECGFPVCRPCYEYERR------EGTQ
VvCesA1 33 HLNGQICQI-------CGDTVGLTATGDVFVACNECAFPVCRPCYEYERK------DGNQ
ZmCesA2 35 SANGQVCQI-------CGDTVGVSATGDVFVACNECAFPVCRPCYEYERK------EGNQ
VvCesA3 14 GLGGQVCQI-------CGDNVGKTVDGEPFIACDVCAFPVCRPCYEYERK------DGNQ
OsCesA2 7 GKQCHVCQI-------CGDGVGTAADGELFTACDVCGFPVCRPCYEYERK------DGSQ
AtCslB5 1 ------------------------------------------------------------
StCslH1 1 ------------------------------------------------------------
VvCslH1 1 ------------------------------------------------------------
VvCslB3 1 ------------------------------------------------------------
VvCslG2 1 ------------------------------------------------------------
AtCslG3 1 ------------------------------------------------------------
AtCslE1 1 ------------------------------------------------------------
VvCslE6 1 ------------------------------------------------------------
AtCslA3 1 ------------------------------------------------------------
AtCslA7 1 ------------------------------------------------------------
PbCCesA 1 ------------------------------------------------------------
BaCesA 1 ------------------------------------------------------------
AtmCesA 1 ------------------------------------------------------------
RlCesA 1 ------------------------------------------------------------
KxCesA3 1 ------------------------------------------------------------
EcCesA 1 ------------------------------------------------------------
PsCesA 219 -L--ERFQESDAHWLVRQIARMQLSARDVVVLDVPCGDLLMLEQALNAASQVLVVLTADA
PfCesA 1 ------------------------------------------------------------
PvCesA3 250 QL--PELPTGATLPAQTITERVRLVC-ENCFATVPAGTAFCTECGEAM------------
PiCesA3 250 QM--PELPTGATLPAQTMTERVRLVC-ENCFATVPAGTAFCTECGEAM------------
PvCesA4 17 EY----CQ----TPLSSA-----QYH-EQLTAAAASGTKLLSQSTMET------------
PiCesA4 17 QY----SQ----TPLSGA-----QYH-EQISSAAAPGKKLLSQATMDV------------
PvCesA2 14 DY--ELHG----TPAT---------------GANDGGAGFYTQEAHLV------------
PiCesA2 14 DY--ELHG----TPAT---------------GANDGGAGFYAQEGRPT------------
PvCesA1 16 DY--SKFA----TP-----------------------------SAMDV------------
PiCesA1 16 DY--EMYA----TP-----------------------------SAMDV------------
NsCesA 1 ------------------------------------------------------------
TvCesA 1 ------------------------------------------------------------
JfCesA 1 ------------------------------------------------------------
TlCesA 1 ------------------------------------------------------------
ThCesA 8 EY--EEMV----------------------------------------------------
GpCesA 1 ------------------------------------------------------------
AmCesA 1 ------------------------------------------------------------
AtCslD6 49 ------------------------------------------------------------
VvCslD3 157 ICPGCKEPYKGEFAAVDNG-------RVLTLSS---------------------------
CrCslD1 1 ------------------------------------------------------------
PpCslD8 184 KCPGCKDDYTVSDEPFSQNT-S--ENDMRALPP---------------------------
AtCesA8 50 ICLRCGNPYDENVFDDVETK-TSKTQSIV-------------------------------
VvCesA8 50 VCLRCSTPYDVSQVKCLAVN-SPESSTMADVET---N-------QSSN------------
VvCesA4 79 CCPQCNTRYKRHKGCARVAGDDEGSLDGDDFND---EFQ-IKNTRDQQNV---FA-----
VvCesA5 55 VCPQCKTRFKRLKGCARVEGDEE-EDDIDDVDN---EFN-FEGRGKVDMQGALAEA----
AtCesA2 80 ACPQCKTRYKRIKGSPRVDGDDEEEEDIDDLEY---EFD-HGMDPE-----HAAEA----
AtCesA6 80 ACPQCKTRFKRLKGSPRVEGDEE-EDDIDDLDN---EFE-YGNNGIGF--DQVSEG----
StCesA1 1 ----------RPQGSQRVDGDDE-EDEFDDLDH---EFD-YD----GT-PRHLSEA----
HaCesA2 80 ACPQCRTRYKRIKGSPKVDGDEE-EEDTDDLEN---EFE-IGVNDRRD-PRHVAEA----
VvCesA6 80 ACPQCKTRYKRIKGSPRVEGDEE-EDDIDDLEN---EFD-FRSNYSRD-PHQVAEA----
VvCesA9 1 ------------------------------------------------------------
PbCesA 79 SCPQCRTRYERHKGSPRVDGDEDED-DTDDLEN---EFN-YRDVHKQD-KQQATDD----
VvCesA7 117 LCPQCKTRFKRLKGCARVEGDDDEE-DIDDIEH---EFN-IDDEQNKN-K-LIAEA----
StCesA7 78 QCPQCKTRYKRLKGSPRVAGDEDEE-DIDDIEH---EFK-VDDEQNKN-R-NIVET----
VvCesA1 80 SCPQCKTRYKRHKGSPRVEGDDEED-DVDDIEN---EFN-YAQGNSKA-R----------
ZmCesA2 82 CCPQCKTRYKRQKGSPRVHGDDEEE-DVDDLDN---EFN-YKQGNGKG-P----------
VvCesA3 61 SCPQCKTRYKRHKGSPAIRGDGEEDGDVDDVVA---DIN-YSSEDQNQ-KQKIAER----
OsCesA2 54 ACPQCKTKYKRHKGSPPILGDESDDVDADDASD---VNY-PTSGNQDH-KHKIAER----
AtCslB5 1 ------------------------------------------------------------
StCslH1 1 ------------------------------------------------------------
VvCslH1 1 ------------------------------------------------------------
VvCslB3 1 ------------------------------------------------------------
VvCslG2 1 ------------------------------------------------------------
AtCslG3 1 ------------------------------------------------------------
AtCslE1 1 ------------------------------------------------------------
VvCslE6 1 ------------------------------------------------------------
AtCslA3 1 ------------------------------------------------------------
AtCslA7 1 ------------------------------------------------------------
PbCCesA 1 ------------------------------------------------------------
BaCesA 1 ------------------------------------------------------------
AtmCesA 1 ------------------------------------------------------------
RlCesA 1 ------------------------------------------------------------
KxCesA3 1 ------------------------------------------------------------
EcCesA 1 -----MSILTRWLL--------IPPVNARL----IGRYRDY-----R---RHGASAFSAT
PsCesA 276 ACYLTLDQMQGWLEP--VLAGPQPPVCHYV----INQFDAS-----RTFSRDMRDVMAKR
PfCesA 1 ------------------------------------------------------------
PvCesA3 295 ------P-----------SEDANPDVSISQAQ----------------MPS-VAMN----
PiCesA3 295 ------P-----------SEDGNPDVSISQAQ----------------MPS-VSMN----
PvCesA4 51 ------Q------------------HTINELTKAKNHEELGKITVHGWMHKQGSRK----
PiCesA4 51 ------Q------------------NTINELTKAKDHEELGKITVHGWMHKQGSRK----
PvCesA2 41 ------H-QQGHAD---PRGPALPPMNVSD-AVGLGGQRDNIISVHGYMHKQGKRT----
PiCesA2 41 ------P-QQGYVD---PRGPALPPMNVSD-AVGLGGQRDNIISVHGYMHKQGKRT----
PvCesA1 29 ------R-QPGETEIVRPGGAFDPPERLPGHPDDGGALQENVITVHGYMHKQGKRT----
PiCesA1 29 ------H-EPGEAEIVRRGS-PDPPVRPPVSSDEGGSFQDNVITVHGYMHKQGKRM----
NsCesA 1 ------------------------------------------------------------
TvCesA 1 ------------------------------------------------------------
JfCesA 1 ------------------------------------------------------------
TlCesA 1 ------------------------------------------------------------
ThCesA 14 ----------------DI-----------------GTF--------RRHHQTGSDD----
GpCesA 1 ------------------------------------------------------------
AmCesA 1 ------------------------------------------------------------
AtCslD6 49 ------------------------------------------------------------
VvCslD3 183 -----------------------------------------------P-V-GVF-----K
CrCslD1 1 ------------------------------------------------------------
PpCslD8 214 -----------------------------------------------PSD-DSS-----R
AtCesA8 78 ------------------------------------------------------------
VvCesA8 87 ----------------------------------------------------------HS
VvCesA4 127 -----------------------------PSENG----------DYNPQQW---------
VvCesA5 106 --------------M------------LQGHMTYGRAYDSDLP--------HVF-----H
AtCesA2 127 --------------A------------LSSRLN--TG----------RGGLDSA-----P
AtCesA6 129 --------------M------------SISRRNSGFP----------QSDLDSA-----P
StCesA1 37 --------------A------------LAARL--GRGTNYNASGLNTPAEVDPA-----A
HaCesA2 130 --------------L------------LSARLNTGRGSQAHVSGFATPSGFDSA-----S
VvCesA6 130 --------------M------------LSAHLNIG--SHAHTSGISTPLDLDSS-----S
VvCesA9 1 ------------------------------------------------------------
PbCesA 129 --------------V------------LHSHMSYGLENDQTMSSMRSQ------------
VvCesA7 166 --------------M------------LHGKMSYGRGPEDDDNAQFPPV-----------
StCesA7 127 --------------I------------LHGKMTYGRGPEDEDSAQYPPV-----------
VvCesA1 124 -----------------------------RQWQG--------------EDADLSSSSRHE
ZmCesA2 126 ----------------------------EWQLQG--------------DDADLSSSARHD
VvCesA3 112 --------------M------------LSWQMTYGRGEDTNYDRE--------------V
OsCesA2 105 --------------M------------LTWRMNSGRNDDIVHSKYDSGEIGHPKYDSGEI
AtCslB5 1 ------------------------------------------------------------
StCslH1 1 ------------------------------------------------------------
VvCslH1 1 ------------------------------------------------------------
VvCslB3 1 ------------------------------------------------------------
VvCslG2 1 ------------------------------------------------------------
AtCslG3 1 ------------------------------------------------------------
AtCslE1 1 ------------------------------------------------------------
VvCslE6 1 ------------------------------------------------------------
AtCslA3 1 ------------------------------------------------------------
AtCslA7 1 ------------------------------------------------------------
PbCCesA 1 ------------------------------------------------------------
BaCesA 1 ------------------------------------------------------------
AtmCesA 1 ------------------------------------------------------------
RlCesA 1 ------------------------------------------------------------
KxCesA3 1 ------------------------------------------------------------
EcCesA 36 LGCFWMILAWIFIPLEHPRWQRIRA--EHKNL-YPHIN----------------------
PsCesA 325 LGGRLLG-------IVHKDNA---L--AEALA-YGHNA----------------------
PfCesA 1 ------------------------------------------------------------
PvCesA3 317 -----------NKSQVPDRWQQV----------------PHRTYMSTTSFVDP-----KH
PiCesA3 317 -----------NKGQVPDRWQHV----------------PHRTYMSTTSFVDP-----KH
PvCesA4 83 -----------FKGPVAKSWRKRYFALEGAKMYYFHSDVDCRKYFNSRN---------GE
PiCesA4 83 -----------FKGPVAKSWRKRYFALEGAKMYYFHSDVDCRKYFNSRN---------GE
PvCesA2 86 -----------IKGPIHKSWKRRYFALEKAKIYYFHSHLECRQYFTTRN---------AD
PiCesA2 86 -----------IKGPIHKSWKRRYFALEKAKIYYFYSHLECRQYFTTRN---------AD
PvCesA1 78 -----------IKGPMHKSWKRRYFALEKAKIYYFHSHQECRLYFTTRN---------TD
PiCesA1 77 -----------MKGPMHKSWKRRYFALEKAKIYYFHSHLECRQYFTTRN---------TD
NsCesA 1 -----------------------------------------------------------M
TvCesA 1 ------------------------------------------------------------
JfCesA 1 ------------------------------------------------------------
TlCesA 1 ------------------------------------------------------------
ThCesA 29 -----------SH-P------RL----------------LSDNMRSNRSS-AA-----HP
GpCesA 1 ------------------------------------------------------------
AmCesA 1 ------------------------------------------------------------
AtCslD6 49 --LSIVLLEIDSNQESV-------------------------------------------
VvCslD3 189 EERRLSLLKSSSPRSTLMKSQTAEFDHN---------------------GWLF-------
CrCslD1 1 ------------------------------------------------------------
PpCslD8 221 LERRLSLLKTKP--GMMSNGSSADFDHA---------------------RWLY-------
AtCesA8 78 ----PTQTNNTSQDSGI--------------------------HARHISTVSTIDSE---
VvCesA8 89 ---TMAAHLNDAQDVGM--------------------------HTRHVSTVSTVDSE---
VvCesA4 139 -------HANGQAFSAA---------------------------------------G--S
VvCesA5 127 TMPQVPLLTNGQMVDDIP----PEQHAL--VP------SFMGGGGKRIHPLPFSDPNL-P
AtCesA2 144 PGSQIPLLTYCDEDADMY----SDRHAL-IVP------PST-GYGNRVYPAPFTDSSA-P
AtCesA6 148 PGSQIPLLTYGDEDVEIS----SDRHAL-IVP------PSLGGHGNRVHPVSLSDPTV-A
StCesA1 64 LNSEIPLLTYGQEDDTIS----ADKHAL-IIP------PFM-GRGKKIHPVPYTDSSM-S
HaCesA2 159 VAPEIPLLTYGEEDVGIS----SDKHAL-IVP------PFN---GKRIHPMPFSDSSL-P
VvCesA6 157 VPSGIPLLTYGQDDVGIS----SDKHAL-IIP------PFM-GRGKRVHPMPFPDSSM-S
VvCesA9 1 ------------------------------------------------------------
PbCesA 151 --------FSLRTVSGMSESNSTSLEHHA-IV------LPPSSGGKRIHPIPYLEGGT-P
VvCesA7 189 -----ITGVRSRPVSGEFP---ISSHAHG-EQ------GLSSSLHKRVHPYPVSEPGS-A
StCesA7 150 -----IAGTRSHPVSGEFP---ISNHGNG-EQ------TLGSSLHKRIHPYPASESGS-A
VvCesA1 141 SQQPIPLLTNGQPLSGEIPSGTPDNQSVRTTS------GPLGPGEKHVHSLPYVDPRQ-P
ZmCesA2 144 PHHRIPRLTSGQQISGEIPDASPDRHSIRSP------------------TSSYVDPSV-P
VvCesA3 132 SHNHIPLLTNGMDVSGELSAASPERLSMAS--------PGAGGGGKRIHPLPYTGDVNQS
OsCesA2 139 PRIYIPSLT-HSQISGEIPGASPDHMMS-----------PVGNIGRRGHPFPYVNHSP--
AtCslB5 1 ------------------------------------------------------------
StCslH1 1 ------------------------------------------------------------
VvCslH1 1 ------------------------------------------------------------
VvCslB3 1 ------------------------------------------------------------
VvCslG2 1 ------------------------------------------------------------
AtCslG3 1 ------------------------------------------------------------
AtCslE1 1 ------------------------------------------------------------
VvCslE6 1 -----------------------------------------------------------M
AtCslA3 1 ------------------------------------------------------------
AtCslA7 1 ------------------------------------------------------------
PbCCesA 1 ------------------------------------------------------------
BaCesA 1 ------------------------------------------------------------
AtmCesA 1 ------------------------------------------------------------
RlCesA 1 ------------------------------------------------------------
KxCesA3 1 ----------------------------------------------------MPEVR---
EcCesA 71 ---------------ASRP------RPLDPVRYLIQTCWLLIGASRKETPKPRRRAF---
PsCesA 350 ---------------VQVPSASPGHRHSGPARAESLADHQTADSGRRRDPIVMTNLS---
PfCesA 1 ----------------------------------------------------MTDTT---
PvCesA3 345 AKEGGVSMKD---NSRSI--RFMDSGVQGPDGKMSQY-NDSIAGVR--------------
PiCesA3 345 AKEGGVSMKD---NGRSI--RFMDSGVQGPDGKMSQY-NDSIAGVR--------------
PvCesA4 123 LVVGAIDLRD---AFKLEQSERLDLPARGIVIHTRHRAWLVCPETD--------------
PiCesA4 123 LVVGAVDLRD---AFKLEQSERLDLPARGIVIHTRHRAWLVCPETD--------------
PvCesA2 126 LVVGAIELKD---ALQLRPCARLDLPHKGFEVHTKRRVWVLCPETD--------------
PiCesA2 126 LVVGAIELKD---ALQLRPCARLDLPHKGFEVHTKRRVWVLCPETD--------------
PvCesA1 118 LVVGAIELKD---ALQLRPCARMDLPHRGFEVMTKRRVWVLCPETD--------------
PiCesA1 117 LVVGAIELKD---ALQLRPCARLDLPHRGFEIMTKRRVWVLCPETD--------------
NsCesA 2 AVIGCLVLPSDVTGLGS-------------------------------------------
TvCesA 1 ------------------------------------------------------------
JfCesA 1 ------------------------------------------------------------
TlCesA 1 ------------------------------------------------------------
ThCesA 49 DDFTAPQWPPSIAGTGSSPSLTLRSGATTPYDHLSSTKHLIYADSR--------------
GpCesA 1 ------------------------------------------------------------
AmCesA 1 ------------------------------------------------------------
AtCslD6 64 --------------PSVSGDIVSGSSGK------------------D--NEPDLTDVRIN
VvCslD3 221 ---------ETKGTYGYGNAIWPEEGGN------------------A--NGENE-----N
CrCslD1 1 ------------------------------------------------------------
PpCslD8 251 ---------QTKGTYGYGNAVWPGEDGY------------------D--GGGGQ-----G
AtCesA8 105 ------------LNDEYGNPIWKNRVESWKDKKDKKSKKKKKDPKATK-AEQHEAQIPTQ
VvCesA8 117 ------------LNDDSGNPIWKNRVESWKDKKSKKKK-------ATS-KAKHEAEIPPE
VvCesA4 151 VAGKDFEG----EKDIYNNDEWKDRVEKWKTRQEKKGLISKDGG----------------
VvCesA5 174 VQPRSMDPSRDLAAYGYGSVAWKERMENWKQKQE-KLQMMKNENG----------GKDWD
AtCesA2 191 PQARSMVPQKDIAEYGYGSVAWKDRMEVWKRRQGEKLQVIKHEGGN---NGR---GSNDD
AtCesA6 196 AHPRPMVPQKDLAVYGYGSVAWKDRMEEWKRKQNEKLQVVRHEGDP---D---------F
StCesA1 111 LPPRPMDPKKDLAVYGYGTVAWKERMEDWKKKQNDKLQVVKHGGK-----G----GANNG
HaCesA2 204 LPPRPMDPKKDLAVYGYGTVAWKERMEEWKKKQSDKLQVVKHQGGK---GG----ENNGG
VvCesA6 204 LPPRPMDPKKDLAVYGYGSVAWKDRMEEWKKKQNDKLQVVKHQGGN---DG----GNFDE
VvCesA9 1 ------------------------------------------------------------
PbCesA 195 VGARPMDPTKDLAQYGYGSVAWKERVESWKLRQGKLQMTMTEGGQLQAGGKGG-P---EE
VvCesA7 233 RW------------DEKKEGGWKERMDDWKMQQGNL----------------G-P---DA
StCesA7 194 RW------------DDKKEGGWKERMEDWKLQQGHV----------------G-Q---DY
VvCesA1 194 VPVRIVDPSKDLNSYGLGNVDWKERVEGWKLKQEKNMMQVTSRYPEGKGDLEGT------
ZmCesA2 185 VPVRIVDPSKDLNSYGLNSVDWKERVESWRVKQDKNMLQVTNKYPEARGDMEGT------
VvCesA3 184 PNIRITDPVREFGSPGLGNVAWKERVDGWKMKQEKNVVPLS--TGHAASEGRGAGDIDAS
OsCesA2 185 ------NPSREF-SGSLGNVAWKERVDGWKMKDKGA-IPMANGTSIAPSEGRGVGDIDAS
AtCslB5 1 ------------------------------------------------------------
StCslH1 1 ------------------------------------------------------------
VvCslH1 1 ------------------------------------------------------------
VvCslB3 1 -------------MQCLLCKEWPA--------QLLSPITSPQTTYT------------IC
VvCslG2 1 ------------------------------------------------------------
AtCslG3 1 -----------------MYQVSLK--------QFVFLLKIKSTTM---------------
AtCslE1 1 ---------------------------------------MVNKDD---------------
VvCslE6 2 LSLR-----NHLPTSRHVGEIWNE--------NYIKAIAYRAEVV---------------
AtCslA3 1 ------------------------------------------------------------
AtCslA7 1 ------------------------------------------------------------
PbCCesA 1 ------------------------------------------------------------
BaCesA 1 ------------------------------------------------------------
AtmCesA 1 ------------------------------------------------------------
RlCesA 1 ------------------------------------------------------------
KxCesA3 6 ---------SST-QSESGMSQWMGKILSI--------R----------------------
EcCesA 107 ---------SGLQNIRGRYHQWMNELPER--------V-SHKTQH---------------
PsCesA 392 ---------LDASPPRTRSSLWLNALSAR--------F-GQQ------------------
PfCesA 6 ---------SSTPFVEGRAEQRLNGAIAR--------F-NRW------------------
PvCesA3 385 ----------------NYYEPSFRSFAMS---------TYSIANR---------------
PiCesA3 385 ----------------NYYEPSFRSFAMS---------TYSIANR---------------
PvCesA4 166 ----------------QDFTMWFDALEFT---------VMSAGSG---------------
PiCesA4 166 ----------------QDFTMWFDALEFT---------VMSAGSG---------------
PvCesA2 169 ----------------EEYRMWFQGVERA---------IVANGAG---------------
PiCesA2 169 ----------------EEYRMWFQGVERA---------IVANGAG---------------
PvCesA1 161 ----------------DEYRLWFEGVEEA---------IVACGSG---------------
PiCesA1 160 ----------------EEYRLWFEGVEEA---------IVACGSG---------------
NsCesA 19 -------------------PVSDKNFITS--------VKMTSINH---------------
TvCesA 1 -------------------------------------MTSLFINN---------------
JfCesA 1 ------------------------------------------------------------
TlCesA 1 ------------------------------------------------------------
ThCesA 95 ----------------SSTPMNGRSPSKE--------VSSDNDDH---------------
GpCesA 1 ------------------------------------------------------------
AmCesA 1 ------------------------------------------------------------
AtCslD6 90 VGEEEEDDTLLSKISYSLTRVVKISPIIIAL-----------------------------
VvCslD3 247 A---CESIKLLSKPWRPLTRKLSIRAAVLSP-----------------------------
CrCslD1 1 ------------------------------------------------------------
PpCslD8 277 PPNLGTLPEFNDKVRRPLTRKVSISTGILSP-----------------------------
AtCesA8 152 QHMEDTPPNTESGATDVLSVVIPIPRTKITS-----------------------------
VvCesA8 157 QQMEEKQS---ADAAQPLSTVVPLPRNKLTP-----------------------------
VvCesA4 191 NDPGDDDDFLLAEARQPLWRKVPIASSKISP-----------------------------
VvCesA5 223 NDGDGPELPLMDEARQPLSRKLPISSSQINP-----------------------------
AtCesA2 245 DELDDPDMPMMDEGRQPLSRKLPIRSSRINP-----------------------------
AtCesA6 244 EDGDDADFPMMDEGRQPLSRKIPIKSSKINP-----------------------------
StCesA1 162 DELDDPDLPKMDEGRQPLSRKMPIASSRLSP-----------------------------
HaCesA2 257 DELDDPDLPMMDEGRQPLSRKLPISSSKISP-----------------------------
VvCesA6 257 DELDDPDLPKMDEGRQPLSRKIPIPSSKINP-----------------------------
VvCesA9 1 ------------------------------------------------------------
PbCesA 251 DDLNGPDLPIMDEARQPLSRKVPFPSSRINP-----------------------------
VvCesA7 261 DDYNDPDMAMIEEARQPLSRKVPIASSKVNP-----------------------------
StCesA7 222 DDSADVDMSMVDEARQPLSRKVPIASSKINP-----------------------------
VvCesA1 248 -GSNGEELQMADDARQPLSRVVPIPSSHLTP-----------------------------
ZmCesA2 239 -GSNGEDMQMVDDARLPLSRIVPISSNQLNL-----------------------------
VvCesA3 242 TDVLVDDSLLNDEARQPLSRKVSIPSSRINP-----------------------------
OsCesA2 237 TDYNMEDALLNDETRQPLSRKVPISSSRINP-----------------------------
AtCslB5 1 -------MADSSSSLHPLCERIS-HKSY--V-----------------------------
StCslH1 1 -------MAPKTPSSLPLYEIKY-RKNY--I-----------------------------
VvCslH1 1 ---------MAKPIPSPLYEKIP-QKNT--L-----------------------------
VvCslB3 28 PTSILLHFPMAKPISSPLHEKFP-QKNT--L-----------------------------
VvCslG2 1 -----------MDSSLPLQLCYV-RKSTAII-----------------------------
AtCslG3 21 -----EPHRKHS-VGDTTLHTCH-PCRRTIP-----------------------------
AtCslE1 7 -----RIRPVHEADGEPLFETRR-RTGRVIA-----------------------------
VvCslE6 34 -----NVWKMGRDGQLPLFETKA-GKGRL-L-----------------------------
AtCslA3 1 ------------------------------------------------------------
AtCslA7 1 ------------------------------------------------------------
PbCCesA 1 -------------MSSSDIESMGYE--P-------LLGNTILDDNIILPSPPTAYEKYLY
BaCesA 1 -------------------------------------------------------MEFIL
AtmCesA 1 -----------------------MS--KAITIIVWLLVSLCVLAIVTMPVSLQTHLVATA
RlCesA 1 ------------MPRLSNFEGTSMR--KARSVIIWAVVSLCMIVLITLPVNLQTQLITSI
KxCesA3 26 ----------------------------GAGLIIGVFGLCALIAATSVTLPPEQQLIVAF
EcCesA 134 ---LD------EKKELGHL-SAGAR--RLILGIIVTFSLILALICVTQPFNPLAQFIFLM
PsCesA 416 --------------------SRTLR--RALKTVAIVVGLLLMALVVTVPLDLYAQCFFAL
PfCesA 30 --------------------PSAPR--TVLVVASCVLGAMLLLGIISAPLDLYSQCLFAA
PvCesA3 405 ---AA------EPVETPNIRKYKMS--GSGMFHVF-------------------------
PiCesA3 405 ---AA------EPVETPNIRKYKMS--GSGMFHVF-------------------------
PvCesA4 186 ---NV------VKRELPNVRVYEMK--GRFSYRLW-------------------------
PiCesA4 186 ---NV------VKRDLPNVRVYEMK--GRSSYRFW-------------------------
PvCesA2 189 ---NI------IERKLPNVRKYLMK--GNQTYRFF-------------------------
PiCesA2 189 ---NI------IERKLPNVRKYLMK--GNQTYRFF-------------------------
PvCesA1 181 ---NI------IERKLPNVRKYYMK--GITTYRVL-------------------------
PiCesA1 180 ---NI------IERKLPNVRKYYMK--GITTYRTL-------------------------
NsCesA 37 -----------DPTFSGNSRSTLKK--RTL------------------------------
TvCesA 9 -----------APTFSGNSRSTLKK--RTL------------------------------
JfCesA 1 ---------------------MVFS--L----RIT-------------------------
TlCesA 1 ------------------------------------------------------------
ThCesA 116 ---NG------VAILHDRDDADIWK--GWRRYLYT-------------------------
GpCesA 1 -------------MILGKEETTTFK--RLLKFT---------------------------
AmCesA 1 ------------------------M--VLLTFNMW-------------------------
AtCslD6 121 -------------------------YRILI--VVRVVSLALFLFWRIRNP----------
VvCslD3 275 -------------------------YRLLV--LVRMAFLGLFLTWRIRNP----------
CrCslD1 1 ------------------------------------------------------------
PpCslD8 308 -------------------------YRLIV--AIRMVVLALFLMWRVQHP----------
AtCesA8 183 -------------------------YRIVI--IMRLIILALFFNYRITHP----------
VvCesA8 185 -------------------------YRGVI--IMRLIILALFFHYRITNP----------
VvCesA4 222 -------------------------YRIVI--VLRLVILAFFFRFRILTP----------
VvCesA5 254 -------------------------YRMII--IIRLVVLGFFFHYRVMHP----------
AtCesA2 276 -------------------------YRMLI--LCRLAILGLFFHYRILHP----------
AtCesA6 275 -------------------------YRMLI--VLRLVILGLFFHYRILHP----------
StCesA1 193 -------------------------YRLSI--LVRLAVVGLFFHYRITHP----------
HaCesA2 288 -------------------------YRLII--ILRLVILGLFFHYRILHP----------
VvCesA6 288 -------------------------YRIII--ILRLVILGFFFHYRILHP----------
VvCesA9 1 ------------------------------------------------------------
PbCesA 282 -------------------------YRMII--VIRLVVIAFFFRYRLLNP----------
VvCesA7 292 -------------------------YRMVI--VARLLVLAFFLRYRILNP----------
StCesA7 253 -------------------------YRMVI--VARLVILAIFLRYRILNP----------
VvCesA1 278 -------------------------YRVVI--ILRLIILGFFLQYRTTHP----------
ZmCesA2 269 -------------------------YRIVI--ILRLIILCFFFQYRISHP----------
VvCesA3 273 -------------------------YRMVI--ILRLIILSIFLHYRITNP----------
OsCesA2 268 -------------------------YRMVI--VLRLIVLCIFLHYRITNP----------
AtCslB5 22 -------------------------LRAVD--LTILGLLYSLLLYRILHI----------
StCslH1 22 -------------------------SRVIE--LFILFLLFSLLAYRFLTL----------
VvCslH1 20 -------------------------HRASD--VTIFFLLLSLLAYRLLSL----------
VvCslB3 56 -------------------------HRALD--LTIFFLLLSLLAYRLLSL----------
VvCslG2 20 -------------------------NRWYT--LIHSTALMALVYYRASFLFQNPE-----
AtCslG3 45 -------------------------YRIYA--VFHTCGIIALMYHHVHSL---L------
AtCslE1 32 -------------------------YRFFS--ASVFVCICLIWFYRIGEI---GD-----
VvCslE6 58 -------------------------FGLYA--VSTFVGICLICVYRLTHL---PE-----
AtCslA3 1 ------------------------------------------------------------
AtCslA7 1 ------------------------------------------------------------
PbCCesA 39 ------------V-KSGR-------WMINTFGLVSSSSLMLGM-WLFVIATG-TY-----
BaCesA 6 HCYPVAMIALILIGFLGSWMNPR--FKKFLIILCLMTNGVYIV-WRFGYTLPVSR-----
AtmCesA 36 ISLILLAT----I----KSFNGQGAWRLVALGFGTAIVLRYVY-WRTTSTLPPVN-----
RlCesA 47 TVVTVMAL----I----KILKGEGTWRLVALAFGTSIVLRYVY-WRTTNTLPPLN-----
KxCesA3 58 VCVVIFFI----V----GHKPSRRS-QIFLEVLSGLVSLRYLT-WRLTETLSFDT-----
EcCesA 182 LLWGVALI----V----RRMPGRFS-ALMLIVLSLTVSCRYIW-WRYTSTLNWDD-----
PsCesA 454 ACFAAMLV----I----RKMPGRIS-VLALVTLSLLASFRYMY-WRLTSTLDFDN-----
PfCesA 68 VCFLAVLV----L----RKIPGRLA-ILALVVLSLVASLRYMF-WRLTSTLGFET-----
PvCesA3 429 -------------------------YFGTA--ATGIFWLYYLTTMYP-QQYFCDHARPTL
PiCesA3 429 -------------------------YFGTA--ATGIFWLYYLTTMYP-QQYFCDHARPTL
PvCesA4 210 -------------------------YIIYI--VTALIELAGIVLWFPLGIEPCDVKYRSD
PiCesA4 210 -------------------------YVIFV--ITALIELAGIVLWFPLGIEPCDVKYKTD
PvCesA2 213 -------------------------YFLFV--IAGIVELLALVFWFAIGLEPCDAARLEV
PiCesA2 213 -------------------------YFLFL--IAGIVELLAIVFWFVIGLEPCDASRLEV
PvCesA1 205 -------------------------YFLFL--LFSIIEIFGLIFWFVVGTQPCDAGYRSL
PiCesA1 204 -------------------------YFLFL--ILSIVEIFGFVFWFVVGVQPCDSGNRTL
NsCesA 54 -------------------------LFRYLAEINLIFGLWYLQ-WRIT----NSI-----
TvCesA 26 -------------------------LFRYLAEINLIFGLWYLQ-WRIT----NSI-----
JfCesA 9 -------------------------FRIFLLLLCTITTISYTV-LRAQ----YTI-----
TlCesA 1 --------------------------------------MVYLG-LRIA----CVI-----
ThCesA 140 -------------------------LTPFLTLLNTGVYLTYLG-LRIA----CVI-----
GpCesA 19 -------------------------TVMSFGLLMLVVGAGYLF-WRVL----NII-----
AmCesA 10 -------------------------GQCMFALLVTGIGWTYLS-WRTW----QLY-----
AtCslD6 144 ---------------NNKAL------WLW------------------------LLSVICE
VvCslD3 298 ---------------NEDAM------WLW------------------------GMSVVCE
CrCslD1 1 ----------------------------------------------------------SE
PpCslD8 331 ---------------NPDAL------WLW------------------------GMSVVCE
AtCesA8 206 ---------------VDSAY------GLW------------------------LTSVICE
VvCesA8 208 ---------------VDSAY------GLW------------------------LTSIICE
VvCesA4 245 ---------------AYDAF------PLW------------------------LISVICE
VvCesA5 277 ---------------VNDAY------ALW------------------------LVSVICE
AtCesA2 299 ---------------VNDAY------GLW------------------------LTSVICE
AtCesA6 298 ---------------VKDAY------ALW------------------------LISVICE
StCesA1 216 ---------------VNDAY------VLW------------------------LLSIICE
HaCesA2 311 ---------------VNDAY------GLW------------------------LTSVICE
VvCesA6 311 ---------------VNDAY------ALW------------------------LTSVICE
VvCesA9 1 ------------------------------------------------------------
PbCesA 305 ---------------VPGAY------GLW------------------------LTSVICE
VvCesA7 315 ---------------VHDAL------GLW------------------------LVSVICE
StCesA7 276 ---------------VHDAI------GLW------------------------LTSIICE
VvCesA1 301 ---------------VKDAY------PLW------------------------LTSVICE
ZmCesA2 292 ---------------VRNAY------GLW------------------------LVSVICE
VvCesA3 296 ---------------VNDAY------PLW------------------------LLSVICE
OsCesA2 291 ---------------VRNAY------PLW------------------------LLSVICE
AtCslB5 45 ----------------SEND------NVW------------------------LLAFFCE
StCslH1 45 ----------------KFHG------LQW------------------------LLALICE
VvCslH1 43 ----------------KNNG------FTW------------------------LLAFLCE
VvCslB3 79 ----------------KNNG------FTW------------------------LLAFLCE
VvCslG2 48 -------------NRAHTPA------SPW------------------------LLVFAGE
AtCslG3 69 ---------------TANTT------LIT------------------------SLLLLSD
AtCslE1 57 -------------NRTVLDR------LIW------------------------FVMFIVE
VvCslE6 83 -------------EG-EVGR------WPW------------------------IGLFLSE
AtCslA3 1 ------------------------------------------------------------
AtCslA7 1 ------------------------------------------------------------
PbCCesA 72 ----------------------------W----------------------FSVFLAVTT
BaCesA 58 ----------------------------P------------------ADIVMGVILIATE
AtmCesA 82 ----------------------------Q-----------------LENFIPGFLLYLAE
RlCesA 93 ----------------------------Q-----------------PENFIPGLLLYLAE
KxCesA3 103 ----------------------------W------------------LQGLLGTMLLVAE
EcCesA 227 ----------------------------P------------------VSLVCGLILLFAE
PsCesA 499 ----------------------------W------------------LDSLLGYGLIVAE
PfCesA 113 ----------------------------W------------------VDMFFGYGLVAAE
PvCesA3 461 PCSELPTSE--TAGCYS--------STVN--FDSASGDGYCIKDVPFMSWVMYAMMIFSE
PiCesA3 461 PCSGLPTSE--TTGCYS--------STVN--FDADSGDGYCIKDVPFMSWLMYAMMIFSE
PvCesA4 243 TCDEIQLLYADPLQCGDKPFNGMWDPPQWYHWSAGIDTVQCFKEPLLSDWIAYFLFYLAE
PiCesA4 243 TCEEIQLLYAETLQCGGKPFNGVWDPPQWYHWSAGIETVQCFKEPHIGDWVSYFLFYLAE
PvCesA2 246 DCNTITLTSLDELRCSSEPFSGYFTPPTWYLQVADVENVICFRDPPIPQWISYFAMILAE
PiCesA2 246 DCATITSTSLETLRCSNEPFSGWFTPPDWYLEVADVDNVICFRDPPIPQWASYFAMLFAE
PvCesA1 238 DCEDVYDSTPDELNCLADPMSGWYTPPDWYMTLAGVDDVACFHNPPLGQWVGFFALVVAE
PiCesA1 237 ACEDVYDNTPDDLNCRADPMSGWFTPPDWYLTLAGVDDVVCFHDPPIAQWVAFFALIAAE
NsCesA 79 ---------------NFDAL--------WLSI--------------------PL--LLAE
TvCesA 51 ---------------NFDAL--------WLSI--------------------PL--LLAE
JfCesA 34 ---------------DQRTPA-----RIWYTTLVV------FLEL---VNGVAFLLMLLE
TlCesA 13 ---------------LAQRAT-----GIT--------------------FAAAWVFIAVE
ThCesA 165 ---------------LAQKSS-----GTT--------------------FAAAWVFIAVE
GpCesA 44 ---------------ALKPS-----------------------------NPVPYIFFALE
AmCesA 35 ---------------LVKST-----------------------------NVIPYLFLSLE
AtCslD6 159 LWFAFSWL---------LDQIPKLFPV--NHATDIEALKATFETPNPDNPTGKSDLPGID
VvCslD3 313 IWFAFSWL---------LDQLPKLCPI--NRSADLNVLKEKFETPNPRNPTGKSDLPGID
CrCslD1 3 VWFTFSWV---------LDQLPKMCPV--NRATDLPVLKEKFDEAGPDNPEGRSDLPGMD
PpCslD8 346 IWFAFSWI---------LDQLPKLCPI--NRLTDLSVLKEKFDMPSPDNPSGRSDLPGVD
AtCesA8 221 IWFAVSWV---------LDQFPKWSPI--NRETYIDRLSARFEREGE-----QSQLAAVD
VvCesA8 223 IWFAVSWV---------LDQFPKWTPI--NRETFIDRLSARYEREGE-----PSELAAVD
VvCesA4 260 IWFAFSWI---------LDQFPKWQPI--NRETYLERLSMRFEREGE-----PNRLSPVD
VvCesA5 292 VWFALSWI---------LDQFPKWLPI--DRETYLDRLSLRYEKEGQ-----PSQLSPVD
AtCesA2 314 IWFAVSWI---------LDQFPKWYPI--ERETYLDRLSLRYEKEGK-----PSGLAPVD
AtCesA6 313 IWFAVSWV---------LDQFPKWYPI--ERETYLDRLSLRYEKEGK-----PSGLSPVD
StCesA1 231 IWFAVSWI---------FDQFPKWCPI--RRETYLDRLSLRYEKEGK-----PSGLAPVD
HaCesA2 326 IWFAVSWI---------LDQFPKWYPI--ERETYLDRLSLRYEKEGK-----PSELASVD
VvCesA6 326 IWFAVSWI---------LDQFPKWYPI--ERETYLDRLSLRYEKEGK-----PSELADID
VvCesA9 1 ------------------------------------------------------------
PbCesA 320 IWFGVSWI---------LDQFPKWLPI--NRETYLDRLSLRYEKEGE-----PSQLAHAD
VvCesA7 330 IWFAFSWI---------LDQFPKWFPI--DRETYLDRLSFRYEREGE-----PNMLSPVD
StCesA7 291 IWFAFSWI---------LDQFPKWFPI--DRETYLDRLSLRYEREGE-----PNMLAPVD
VvCesA1 316 IWFALSWL---------LDQFPKWYPI--NRETFLERLALRYDREGE-----PSQLAPID
ZmCesA2 307 VWFALSWL---------LDQFPKWYPI--NRETYLDRLALRYDREGE-----PSQLAPID
VvCesA3 311 IWFAMSWI---------LDQFPKWLPV--NRETYLDRLALRYDREGE-----PSQLAAVD
OsCesA2 306 IWFALSWI---------LDQFPKWSPI--NRETYLDRLALRYDREGE-----PSQLAPVD
AtCslB5 59 SCFSLVWL---------IFTCLKWSPA--EDIPYINTLNE--R---------VHDLPSLD
StCslH1 59 SWFTFIWI---------LTVSSKWNQV--EPTTYPLRLLE--R---------TLKFPAVD
VvCslH1 57 SWFTFIWI---------LNLSSKWNPV--SYKTYPERLLQCYR---------VDELPPVD
VvCslB3 93 SWFTFIWI---------LNVSTKWNPV--SYKTYPERLLQCYR---------VDELPPVD
VvCslG2 65 LILSFIWL---------LGQAYRWRPV--TRTLFPERLPED------------KHLPAID
AtCslG3 84 IVLAFMWA---------TTTSLRYKPV--RRTEYPEKYAAE-----------PEDFPKLD
AtCslE1 74 IWFGLYWV---------VTQSSRWNPV--WRFPFSDRLSRRY----------GSDLPRLD
VvCslE6 99 LWYILYWF---------VILSVRWSPI--YRNTFKDRLTQRY----------EKVLPGID
AtCslA3 1 ------------------------------------------------------------
AtCslA7 1 ------------------------------------------------------------
PbCCesA 82 VYFFSSYL-------MVNCAGKDFNLK-----------FHKNVKQLH-----SGETPAVD
BaCesA 72 CIGFLQ-L-------LV-FYTLVWKER-----------KREPETLGD-----QDHWPSVD
AtmCesA 97 MYSVVM-L-------ALSLVIVSMPLP-----------SRKT-RPGS-----PDYRPTVD
RlCesA 108 MYSVAM-L-------ALSLFIVATPLP-----------SRPS-RAAN-----PGRLPHVD
KxCesA3 117 LYALMM-L-------FLSYFQTIAPLH-----------RAPLPLPPN-----PDEWPTVD
EcCesA 241 TYAWIV-L-------VLGYFQVVWPLN-----------RQPVPLPKD-----MSLWPSVD
PsCesA 513 FYTLIV-I-------VLGYVQTAWPLH-----------RKPVIMPSD-----SSQWPTVD
PfCesA 127 FYALIV-L-------IFGYVQTAWPLR-----------RTPVWLKTE-----PEEWPTVD
PvCesA3 509 FLNFFLGL---------LFNFSMWRPI--RRGA---RFMNDFKPPIP-----KEQWPTVD
PiCesA3 509 FLNYFLGL---------LFNFSMWRPI--RRGA---RYMNDFKPPIP-----KEQWPTVD
PvCesA4 303 FISISLGF---------LYYLGMWKPV--RRGA---RYLRDFEPHFP-----PSKWPTVD
PiCesA4 303 FISISLGF---------LYYLGMWKPV--RRGA---RYLRDFEPHFP-----PEKWPTVD
PvCesA2 306 LLTFALGV---------LYYLGMWKPV--RRGA---HYF**DEFEPPVP**-----**DELWPKVD**
PiCesA2 306 VLTFALGV---------LYYLGMWKPV--RRGA---HYFDEFEPPVP-----DDLWPKVD
PvCesA1 298 LVSIVLGA---------LYYLGMWKPV--RRGA---HYFDEFDPKVP-----DDLWPKID
PiCesA1 297 LVSIVLGA---------LYYLGMWKPV--RRGA---HYFDEFDPKVP-----DDLWPKID
NsCesA 94 IYSYLGGV---------MFVIGLWRPL--VRQI---KSLDQLTPSLP-----QTDWPIVD
TvCesA 66 IYSYIGGV---------MFVIGLWRPL--VRQI---KSLDQLTPPLP-----PTDWPIVD
JfCesA 65 RVRSNASPGLPIPSSKRVPGDVQRSSSSTLKAT---DLNEVADKSNI-----DAVWPTVT
TlCesA 33 LAVAIPSL-------M-HNGWTMWSLK-----S---RHRPKMRLV-------GNDVPTVD
ThCesA 185 LVVAVPSL-------M-HNIWTMWSLK-----K---RHRPKMRLT-------GNDVPTVD
GpCesA 60 AILYTSAI-------V-WLIDLLWSEP-----P---DRSQP----PP-----PKRWPTVD
AmCesA 51 IMLYTSSV-------L-WFVDMLWTRD-----Y---RRRPAILPVEN-----HAEWPTMD
AtCslD6 208 VFVSTADAEKEPPLVTANTILSILSVDYPV-EKLSVYISDDGGSLVTFEAIAEAASFAKI
VvCslD3 362 MFVSTADPEKEPPLVTANTILSILAADYPV-EKLSCYVSDDGGALLTFEAMAEAASFANL
CrCslD1 52 IFVSTADPEKEPPLVTANTILSILAADYPV-EKLACYLSDDGGALLTFEAMAEAASFAQV
PpCslD8 395 IFVSTADPEKEPPLTTANTILSILASEYPL-EKLACYLSDDGGALLSFEALAEAASFARV
AtCesA8 265 FFVSTVDPLKEPPLITANTVLSILALDYPV-DKVSCYVSDDGAAMLSFESLVETADFARK
VvCesA8 267 FFVSTVDPLKEPPLITANTVLSILAVDYPV-DKVSCYVSDDGSAMLSFESLVETADFARK
VvCesA4 304 VFVSTVDPLKEPPIITANTVLSILSLDYPV-EKVSCYVSDDGASMLLFDSLAETAEFARR
VvCesA5 336 IFV-------------------------------SCYVSDDGAAMLTFEALSETSEFARK
AtCesA2 358 VFVSTVDPLKEPPLITANTVLSILAVDYPV-DKVACYVSDDGAAMLTFEALSDTAEFARK
AtCesA6 357 VFVSTVDPLKEPPLITANTVLSILAVDYPV-DKVACYVSDDGAAMLTFEALSETAEFARK
StCesA1 275 IFVSTVDPLKEPPLITANTVLSILACDYPV-DKVSCYVSDDGAAMLTFEALSETSEFARK
HaCesA2 370 VFVSTVDPMKEPPLITANTVLSILAVDYPV-EKVACYVSDDGAAMLTFEAISETSEFARK
VvCesA6 370 IFVSTVDPMKEPPLITANTVLSILAVDYPV-EKVACYVSDDGAAMLTFEALSETSEFARR
VvCesA9 1 --------MKEPPLITANTVLSILAVDYPV-EKVACYVSDDGAAMLTFEALSETSEFARR
PbCesA 364 IFVSTVDPAKEPPLVTANTMLSILAVDYPV-DKVSCYVSDDGAAMLTFEALSETSEFARK
VvCesA7 374 IFVSTVDPLKEPPLVTANTVLSILAMDYPV-DKISCYISDDGASILTFEALSETAEFARR
StCesA7 335 VFVSTVDPMKEPPLVTANTILSILAMDYPI-DKISCYLSDDGASMCTFEALSETAEFARK
VvCesA1 360 VFVSTVDPLKEPPLVTANTVLSILAVDYPV-DKVSCYVSDDGSAMLTFEALSETSEFARK
ZmCesA2 351 VFVSTVDPLKEPPLITANTVLSILAVDYPV-DKVSCYVSDDGSAMLTFESLSETAEFARK
VvCesA3 355 IFVSTVDPLKEPPLVTANTVLSILAVDYPV-DKVSCYVSDDGAAMLTFEALSETSEFARK
OsCesA2 350 IFVSTVDPMKEPPLVTANTVLSILAVDYPV-DKVSCYVSDDGAAMLTFDALAETSEFARK
AtCslB5 97 MFVPTADTVRESPIITVNTVLSLLAVNYPA-NKLACYVSDDGCSPLTYFSLKEASKFVKI
StCslH1 97 IFVTTADPVLEPPLITINTVLSLLAVDYPA-NKLACYVSDDGASIVTYYSLVEASKFAKH
VvCslH1 97 MFVTTADPMLEPPIITVNTVLSLLAVDYPA-NKLSCYVSDDGASPLTFFALLEASKFAKL
VvCslB3 133 MFVTTADPMLEPPIITVNTVLSLLAVDYPA-NKLSCYVSDDGASPLTFYALLEASKFAKL
VvCslG2 102 VFICTADPKREPTFGVMNTVISAMALDYPP-ERLHVYVSDDGGSSLTLYGMKEAWAFARS
AtCslG3 122 VFICTADPYKEPPMMVVNTALSVMAYEYPS-DKISVYVSDDGGSSLTLFALMEAAKFSKH
AtCslE1 113 VFVCTADPVIEPPLLVVNTVLSVTALDYPP-EKLAVYLSDDGGSELTFYALTEAAEFAKT
VvCslE6 138 IFVCTANPIIEPPTMVINTVLSVMAYDYQP-EKLSIYLSDDGGSCLTFYALLEASQFSKI
AtCslA3 1 --------------MIGSSTIYGT------------------------------------
AtCslA7 1 ------------------------------------------------------------
PbCCesA 119 IFLPV---CGEDLLIINNTWNYVSKLDWPN---LRVHVLDDGK-----------------
BaCesA 107 ILIAT---YNEERHVLKKSVAGCLSLDYPK-ELVNIYLCDDGR-----------------
AtmCesA 132 VFVPS---YNEDAELLANTLAAAKNMDYPA-DRFTVWLLDDGGSVQK---RNASN-----
RlCesA 143 VFVPS---YNEDAGLLGNTLAAAKAMDYPA-EKLHVWLLDDGGTLQK---RNSGK-----
KxCesA3 153 IFVPT---YNEELSIVRLTVLGSLGIDWPP-EKVRVHILDDGR-----------------
EcCesA 277 IFVPT---YNEDLNVVKNTIYASLGIDWPK-DKLNIWILDDGG-----------------
PsCesA 549 VFIPS---YNEALSIVKLTIFAAQSIDWPR-DKLRVYVLDDGR-----------------
PfCesA 163 VFIPT---YNEALSIVKLTIFAAQAMDWPK-DKLRVHVLDDGR-----------------
PvCesA3 550 IFLCH---YMEPVTDSMQTLKNCLAMQYPP-ELLHIFILDDGYTKSV---WDANNHF-KV
PiCesA3 550 IFLCH---YMEPVTDSMQTLKNCLAMQYPP-ELLHIFILDDGYTKSV---WDANNHF-KV
PvCesA4 344 ILLCH---YAEPAEDTIATLEKIMNLDYPP-HLVHVWICDDGYCKAK---WEPGAPVPKV
PiCesA4 344 ILLCH---YAEPADDTIATLEKIMNLDYPP-HLFHVWICDDGYCKAK---WEAGATVPKV
PvCesA2 347 **ILLCH**---**YSEPAEEAIDTLMACMNVQYPP**-**HLLQIWVCDDGYCKTK**---**WTKGNPIPTV**
PiCesA2 347 VLLCH---YSEPAEETIDTLMACMNLQYPP-HLLQIWVCDDGYCKTK---WTKGNPVPTV
PvCesA1 339 VLLCH---YSEPAEETIDTLLACINLQYPP-HLLQIYVLDDGYCSTK---WTKGNPVPAI
PiCesA1 338 VLLCH---YSEPAEETIDTLMACMNLQYPP-HLVQIYVLDDGYCGSK---WTKGNPVPAI
NsCesA 135 VFITC---YNEPPEMVEQTAQAALAIDYPP-TKLYVYVLDDGNSPA-MRAMTEKLCI--A
TvCesA 107 VFITC---YNEPPEMVEQTAQAALAIDYPP-TKLYVYVLDDGNSPT-MRAMTERLCI--A
JfCesA 117 ILITC---CGEDPEVIRGTVLAAQRVRYPVASKVQVYLCDDGKNLDGIPLHVDDLLP---
TlCesA 70 VFVTC---CGEDDEVVMDTVRGALDQDYPG-DKFRVIVLDDAKSAG-LQREC--------
ThCesA 222 CFVTC---CGEDDELVMDTVRGACDQDYPI-DRFRVIVLDDGKSAG-LERSC--------
GpCesA 95 ILVPC---CTEPTELIKDTVKAALGQDYPR-ECFMVHICDDGGDDV-LKDWI--------
AmCesA 90 IFIPC---CKEPTELVKDTVMAALWQDYPA-DKFMVYVCDDGGDDA-LREWV--------
AtCslD6 267 --WVPFCRKHKIEPRNPESYFGLKRDPYKDKV--RHDFVRERRYVKRAYDEFKVRVNALP
VvCslD3 421 --WVPFCRKHDIEPRNPESYFTLKRDPYKNKV--RPDFVRERRRVKREYDEYKVRINGLP
CrCslD1 111 --WIPFCRKHAIEPRNPDSYFNMKGDPTKNQM--RQDFVRDRRRVKREYDEFKVRINGLP
PpCslD8 454 --WIPFCRKHKIEPRNPETYFLLKGDPTKNKV--RSDFVKDRRKVKREYDEFKVRVNGLP
AtCesA8 324 --WVPFCKKYSIEPRAPEFYFSLKIDYLRDKV--QPSFVKERRAMKRDYEEFKIRMNALV
VvCesA8 326 --WVPFCKKFSIEPRAPEFYFSQKIDYLKDKI--QPSFVKERRAMKRDYEEFKVRVNALV
VvCesA4 363 --WVPFCKKHSIEPRAPEFYFSQKIDYLKDKV--DPSFVKERRAMKREYEEFKVRINALV
VvCesA5 365 --WVPFCKKFNIEPRAPEFYFAQKIDYLKDKV--LPSFVKERRAMKREYEEFKVRINALV
AtCesA2 417 --WVPFCKKFNIEPRAPEWYFSQKMDYLKNKV--HPAFVRERRAMKRDYEEFKVKINALV
AtCesA6 416 --WVPFCKKYCIEPRAPEWYFCHKMDYLKNKV--HPAFVRERRAMKRDYEEFKVKINALV
StCesA1 334 --WVPFCKKFNIETRAPEWYFSLKVDYLKNKV--HPSFVRERRAMKRDYEEFKVRINGLV
HaCesA2 429 --WVPFCKRFSIEPRAPEWYFAQKVDYLKDRV--DPAFIRERRAMKREYEEFKVRINGLV
VvCesA6 429 --WVPFCKKFSIEPRAPEWYFAQKVDYLKDKV--HPEFVRERRAMKREYEEFKIRINALV
VvCesA9 52 --WVPFCKKFSIEPRAPEWYFAQKVDYLKDKV--HPEFVRERRAMKREYEEFKIRINALV
PbCesA 423 --WVPFCKKFNIEPRAPEAYFALKIDYLKDRV--QPTFVKERRAMKREYEEFKVRVNALV
VvCesA7 433 --WVPFCKKFSIEPRAPEMYFSLKIDYLKDKV--QPTFVKERRAMKREYEEFKVRINAIV
StCesA7 394 --WVPFCKKFAIEPRAPEFYFSLKIDYLKDKV--QPTFVKERRAMKREYEEFKVRVNALV
VvCesA1 419 --WVPFCKKHNIEPRAPEFYFAQKIDYLKDKI--QPSFVKERRAMKREYEEFKIRINALV
ZmCesA2 410 --WVPFCKKHNIEPRAPEFYFAQKIDYLKDKI--QPSFVKERRAMKREYEEFKIRINALV
VvCesA3 414 --WVPFCKKYSIEPRAPEWYFALKIDYLKDKV--QPSFVKDRRAMKREYEEFKVRVNGLV
OsCesA2 409 --WVPFCKKYSIEPRAPEWYFAQKIDYLKDKV--QASFVKDRRAMKREYEEFKVRVNALV
AtCslB5 156 --WAPFCKKYNVRVRAPFRYFLNPLVATDD-----SVFSKDWKMMKREYVKLCRKVEDAT
StCslH1 156 --WVPFCKKYNIALRAPFRYFSGNSSPPQDS---SQEFQQDWTRMKDEYKQLCKKIEDAS
VvCslH1 156 --WVPFCKKYGIQPRAPFRYFSRELLPSHDN---SMEFLQEYRKIKEEYEELRRRIEDAT
VvCslB3 192 --WVPFCKKYGIQTRAPFRYFSRELLPSHDN---STEFLQEYRKIMDEYEELRRRIEHAT
VvCslG2 161 --WLPFCRTHGIKTRCPEAYFSSAEDDEGAD----LRGTEFFEERK--------------
AtCslG3 181 --WLPFCKKNNVQDRSPEVYFSSKLRSRS----------DEAENIKMMYEDMKSRVEHVV
AtCslE1 172 --WVPFCKKFNVEPTSPAAYLSSKANCLD----------SAAEEVAKLYREMAARIETAA
VvCslE6 197 --WLPFCKKFKVEPRCPEAYFSSTPKPHHDD----PLMAEEWSSIKKLYEDMRNRIEAVM
AtCslA3 11 -----FLTYEMIWKRQELVN----------------AECDKW------------------
AtCslA7 1 ---------MIPRIQPELVK----------------KECDRW------------------
PbCCesA 156 ---------------SDEVK----------------QLTG--------------------
BaCesA 146 ---------------RTDIQ----------------KLAE--------------------
AtmCesA 180 -----IVEAQAAQRRHEELK----------------KLCE--------------------
RlCesA 191 -----LLEAQAAAARHIELK----------------QLCD--------------------
KxCesA3 192 ---------------RPEFA----------------AFAA--------------------
EcCesA 316 ---------------REEFR----------------QFAQ--------------------
PsCesA 588 ---------------REDFR----------------EFCE--------------------
PfCesA 202 ---------------RDDFR----------------EFCR--------------------
PvCesA3 602 TVNTKVIEI-AGDLRGDLARLMHERVV--GP-VQDDQSLKSWRRQHSSVRE---------
PiCesA3 602 TVNTKVIEV-AGDLRGDLARLMHERVV--GP-VQDDQSLKSWRRQHSSVRE---------
PvCesA4 397 SVNHGVIED-AGDVRHEVAQFMYDRVC--ES-Y--ELEVDEWRKEHTTVKM---------
PiCesA4 397 SVNTGVIED-AGDVRHEVAQFMYDRVC--ES-Y--ELEVDEWRKEHTTVKM---------
PvCesA2 400 **ELNKGILET**-**AGDLRQEVAQFMYDRVC**--**DP**-**NE**-**DMEVYAWRKLHSSANL**---------
PiCesA2 400 ELNKGILET-AGDLRQEVAQFMYDRVC--DP-NE-DMEVYAWRKLHSSANL---------
PvCesA1 392 ELNKVILEK-AGDLRQEVAQFMYDRVC--DP-NE-DMEVYAWRKLHSSANL---------
PiCesA1 391 ELNKVVLEK-SGDLRQEVAQFMYDRVC--DP-NE-DMEVYAWRKLHSSANL---------
NsCesA 188 DLQSPLLQQEANRIDAERSH-LVERLQKLESFTSNIQVAEEWLSKSSSVPN---------
TvCesA 160 DLQSPLLQQEANRIDAERSH-LVERLQNLESFTPNIQVAEEWLSKSSSVPS---------
JfCesA 171 ------------------------------------------------------------
TlCesA 117 ------------------------------------------------------------
ThCesA 269 ------------------------------------------------------------
GpCesA 142 ------------------------------------------------------------
AmCesA 137 ------------------------------------------------------------
AtCslD6 323 HSIRRRSDAFNSKEEIKALEKWKHWKVKVEEDQIKEPRPALVAPKATW-MSDGTHWPGTW
VvCslD3 477 DSIRRRSDAYNAREEIKALKLQ------RQNKNDDETLENVKVPKATW-MADGTHWPGTW
CrCslD1 167 ESIRRRSDAYNAHEEIKAKRQQ------IE--AGLEPIEPLNVSKATW-MADGTYWPGAW
PpCslD8 510 DSIRRRSDAYNAHEEIRAKRHQ------ME--SGGDPSEPLNIPKATW-MADGTHWPGTW
AtCesA8 380 AKAQ-------------------------------------KTPEEGWTMQDGTSWPGNN
VvCesA8 382 AKAQ-------------------------------------KTPEEGWTMQDGTAWPGNN
VvCesA4 419 AKAQ-------------------------------------KKPEEGWTMQDGTPWPGNI
VvCesA5 421 AKAQ-------------------------------------KVPEEGWTMQDGTPWPGNN
AtCesA2 473 ATAQ-------------------------------------KVPEEGWTMQDGTPWPGNN
AtCesA6 472 ATAQ-------------------------------------KVPEDGWTMQDGTPWPGNS
StCesA1 390 ATAQ-------------------------------------KVPEDGWTMQDGTPWPGNL
HaCesA2 485 ATAQ-------------------------------------KVPEDGWTMQDGTPWPGNN
VvCesA6 485 SMAQ-------------------------------------K------------------
VvCesA9 108 SMAQ-------------------------------------KVPEEGWTMQDGTPWPGNN
PbCesA 479 AKAQ-------------------------------------KVPEEGWTMQDGTPWPGNN
VvCesA7 489 AKAV-------------------------------------KVPPEGWIMQDGTPWPGNN
StCesA7 450 AKAT-------------------------------------KMPPGGWIMQDGTPWPGNN
VvCesA1 475 AKAQ-------------------------------------KTPEEGWTMQDGTPWPGNN
ZmCesA2 466 AKAQ-------------------------------------KVPEEGWTMADGTAWPGNN
VvCesA3 470 AKAQ-------------------------------------KIPEEGWIMQDGTPWPGNN
OsCesA2 465 AKAQ-------------------------------------KVPEEGWIMQDGTPWPGNN
AtCslB5 209 GDSHW-L--------DA--------------------------------DDDFEAFS-N-
StCslH1 211 T----QE--------PE--------------------------------TCDFDVFS-N-
VvCslH1 211 VKSISYE--------LS--------------------------------TADFVAFS-N-
VvCslB3 247 LKSISHE--------LS--------------------------------TADFVAFS-N-
VvCslG2 201 --------------------KI---------------------------KKEFELFRER-
AtCslG3 229 ESGKVET-------AFITCDQF---------------------------RGVFDLWTDK-
AtCslE1 220 RLGRIPE-------EAR--VKY---------------------------GDGFSQWDAD-
VvCslE6 251 NVGQITE-------EIR--K-Q---------------------------HKGFGEWNLA-
AtCslA3 32 ------------------------------------------------------------
AtCslA7 18 ------------------------------------------------------------
PbCCesA 165 ------------------------------------------------------------
BaCesA 155 ------------------------------------------------------------
AtmCesA 199 ------------------------------------------------------------
RlCesA 210 ------------------------------------------------------------
KxCesA3 201 ------------------------------------------------------------
EcCesA 325 ------------------------------------------------------------
PsCesA 597 ------------------------------------------------------------
PfCesA 211 ------------------------------------------------------------
PvCesA3 649 -----------------------------LRKEG--------------------------
PiCesA3 649 -----------------------------LRKEG--------------------------
PvCesA4 442 -----------------------------PQNSK--------------------------
PiCesA4 442 -----------------------------PTNAN--------------------------
PvCesA2 446 -----------------------------**PSPSR**--------------------------
PiCesA2 446 -----------------------------PSPSR--------------------------
PvCesA1 438 -----------------------------PSSSR--------------------------
PiCesA1 437 -----------------------------PSPSR--------------------------
NsCesA 238 -----------------------------QDKTTDEVL--------AQSLQQFIRWLSPK
TvCesA 210 -----------------------------QDTTKTEVL--------AQSLQQFIRWLSPK
JfCesA 171 ------------------------------------------------------------
TlCesA 117 ------------------------------------------------------------
ThCesA 269 ------------------------------------------------------------
GpCesA 142 ------------------------------------------------------------
AmCesA 137 ------------------------------------------------------------
AtCslD6 382 AV--SGPHHSRGDH-ASVIQVLLDPPG----------------DEPVEGKGGEGRALDLE
VvCslD3 530 VV--PGPEHSKGDH-AGIIQVMLKPPS----------------DEPLNGSSIDANPIDLT
CrCslD1 218 ST--PTVDQGRGDH-AGIIQVMLAPPS----------------SEPLFGNSGDDNLIDTT
PpCslD8 561 TH--SGKEHGRGDH-AGIIQVMLAPPT----------------AEPLMGSSDEENIIDTT
AtCesA8 403 T----------RDH-PGMIQV-------------------------FLGYSG---ARDIE
VvCesA8 405 P----------RDH-PGMIQV-------------------------FLGHSG---AHDIE
VvCesA4 442 T----------RDH-PGMIQVLQGYSNSFGRTNLFYDVTNATSLQVYLGSEG---ALDVE
VvCesA5 444 I----------RDH-PGMIQV-------------------------FLGQSG---GHDTD
AtCesA2 496 V----------RDH-PGMIQV-------------------------FLGHSG---VRDTD
AtCesA6 495 V----------RDH-PGMIQV-------------------------FLGSDG---VRDVE
StCesA1 413 V----------RDH-PGMIQV-------------------------FLGNDG---VRDIE
HaCesA2 508 V----------RDH-PGMIQV-------------------------FLGHNG---VHDVE
VvCesA6 490 --------------------V-------------------------FLGHNG---VRDVE
VvCesA9 131 I----------FFLYFCLAQV-------------------------FLGHNG---VRDVE
PbCesA 502 T----------RDH-PGMIQV-------------------------FLGHSG---GRDTN
VvCesA7 512 T----------KDH-PGMIQV-------------------------FLGHSG---GLDAE
StCesA7 473 T----------RDH-PGMIQV-------------------------FLGQSG---GTDVD
VvCesA1 498 P----------RDH-PGMIQW----------------------------------GLDTD
ZmCesA2 489 P----------RDH-PGMIQV-------------------------FLGHSG---GLDTD
VvCesA3 493 T----------RDH-PGMIQV-------------------------FLGQSG---GLDTE
OsCesA2 488 T----------RDH-PGMIQV-------------------------FLGHSG---GLDTE
AtCslB5 226 --------TKPNDH-STIVKVVWENK--------------------------GGVGD---
StCslH1 225 --------IQQKNH-PTIIKVILENK--------------------------EGVAD---
VvCslH1 229 --------IKKGSH-PTIIKVILENK--------------------------ESRSD---
VvCslB3 265 --------IKKGSH-PTIIKVILENK--------------------------ESRSD---
VvCslG2 213 --------VMR------------------------------------------ATENGAE
AtCslG3 254 --------FTRHDH-PTIIQVLQNSE---------------------------NDMDDTK
AtCslE1 243 --------ATRRNH-GTILQVLVDGR--------------------------EGNTI---
VvCslE6 273 --------SEPQNH-QTILQILIDGR--------------------------DGKAVDVE
AtCslA3 32 ------------------------------------------------------------
AtCslA7 18 ------------------------------------------------------------
PbCCesA 165 ------------------------------------------------------------
BaCesA 155 ------------------------------------------------------------
AtmCesA 199 ------------------------------------------------------------
RlCesA 210 ------------------------------------------------------------
KxCesA3 201 ------------------------------------------------------------
EcCesA 325 ------------------------------------------------------------
PsCesA 597 ------------------------------------------------------------
PfCesA 211 ------------------------------------------------------------
PvCesA3 654 -G----KGVQRRDC--------------------------------AVGS----LSDDYD
PiCesA3 654 -G----KGVQRRDC--------------------------------AVGS----LSDDYD
PvCesA4 447 -P----RLVNRVDC--------------------------------AVGS----VRDDYM
PiCesA4 447 -P----RIVNRSDC--------------------------------AVGS----VRDDYH
PvCesA2 451 -**C**----**KVVNRADC**--------------------------------**AVGS**----**FRDDYR**
PiCesA2 451 -V----KVVNRADC--------------------------------AVGS----FRDDYR
PvCesA1 443 -P----KVVNRADC--------------------------------AVGS----FRDDYR
PiCesA1 442 -P----KVVNRADC--------------------------------AVGS----FRDDYR
NsCesA 261 HHSIAERLKTERQV--------------------------------LAGA----IRQK-E
TvCesA 233 HHSVTERLKTERQA--------------------------------LAAA----IRQK-E
JfCesA 171 -------------------------------------------------I----VLDP-S
TlCesA 117 -----------------------------------------------------------A
ThCesA 269 -----------------------------------------------------------A
GpCesA 142 --------------------------------------------------------QN-E
AmCesA 137 --------------------------------------------------------EN-E
AtCslD6 423 GVDIRLPMLVYVSREKRPGYDHNKKAGAMNALVRASAI--MSNGPFILNLDCDHYVYNSR
VvCslD3 571 EVDIRLPMLVYVSREKRPGYDHNKKAGAMNALVRASAI--MSNGPFILNLDCDHYIYYSE
CrCslD1 259 EVDIRLPMLVYVSREKRPNYDHNKKAGAMNALVRASAI--MSNGPFILNLDCDH------
PpCslD8 602 DVDIRLPMLVYMSREKRPGYDHNKKAGAMNALVRTSAV--MSNGPFILNLDCDHYIFNSL
AtCesA8 424 --GNELPRLVYVSREKRPGYQHHKKAGAENALVRVSAV--LTNAPFILNLDCDHYVNNSK
VvCesA8 426 --GNELPRLVYVSREKRPGYQHHKKAGAENALVRVSAV--LTNAPFILNLDCDHYVNNSK
VvCesA4 488 --GKELPRLVYVSREKRPGYQHHKKAGAMNALIRVSAV--LTNAPFMLNLDCDHYINNSK
VvCesA5 465 --GNELPRLVYVSREKRPGFNHHKKAGAMNALVRVSAV--LTNAPYLLNLDCDHYINNSK
AtCesA2 517 --GNELPRLVYVSREKRPGFDHHKKAGAMNSLIRVSAV--LSNAPYLLNVDCDHYINNSK
AtCesA6 516 --NNELPRLVYVSREKRPGFDHHKKAGAMNSLIRVSGV--LSNAPYLLNVDCDHYINNSK
StCesA1 434 --GNVLPRLIYVSREKRPGFDHHKKAGAMNALMRVSAV--ISNAPYMLNVDCDHYINNSK
HaCesA2 529 --GNELPRLVYVSREKRPGFDHHKKAGAMNSLVRVSAI--ITNAPYMLNVDCDHYINNSK
VvCesA6 502 --GNELPRLVYVSREKRPGFDHHKKAGAMNALMRVSAI--ISNAPYLLNVDCDHYINNSK
VvCesA9 153 --GNELPRLVYVSREKRPGFDHHKKAGAMNALMRVSAI--ISNAPYLLNVDCDHYINNSK
PbCesA 523 --GNELPRLVYVSREKRPGFDHHKKAGAMNALVRVSAV--LTNAPFFLNLDCDHYINNSK
VvCesA7 533 --GNELPRLVYVSREKRPGFHHHKKAGAMNALIRVSAV--LTNAPFMLNLDCDHYLNNSK
StCesA7 494 --GHELPRLVYVSREKRPGFQHHKKAGAMNALVRVAGV--LTNAPFMLNLDCDHYLNNSK
VvCesA1 513 --GNELPRLVYVSREKRPGFQHHKKAGAMNALIRVSAV--LTNGAYLLNVDCDHYFNNSK
ZmCesA2 510 --GNELPRLVYVSREKRPGFQHHKKAGAMNALIRVSAV--LTNGAYLLNVDCDHYFNSSK
VvCesA3 514 --GNELPRLVYVSREKRPGFQHHKKAGAMNALVRVSAV--LTNGPFLLNLDCDHYINNSK
OsCesA2 509 --GNELPRLVYVSREKRPGFQHHKKAGAMNALVRVSAV--LTNGQYLLNLDCDHYINNSK
AtCslB5 248 --EKEVPHLVYISREKRPNYLHHYKTGAMNFLLRVSGL--MTNAPYTLNVDCDMYANEPD
StCslH1 247 ----GLPHLVYISREKRPKHPHQFKAGAMNVLTRVSGV--MTNAPFMLNVDCDMYANNPQ
VvCslH1 251 ----GLPHLVYVSREKHPKHPHHYKAGAMNVLTRVSGA--MTNAPFMLNVDCDMYANNPQ
VvCslB3 287 ----GLPHLVYVSREKDPKHPHHYKAGAMNVLTRVSGA--MTNAPFMLNVDCDMYANNPQ
VvCslG2 223 --EAEMPILVYVSREKTYSHPHHFKAGALNVLLRVSSM--ISNSPYILVLDCDMYCNDPA
AtCslG3 278 --KYIMPNLIYVSREKSKVSSHHFKAGALNTLLRVSGV--MTNSPIILTLDCDMYSNDPA
AtCslE1 265 ----AIPTLVYLSREKRPQHHHNFKAGAMNALLRVSSK--ITCGKIILNLDCDMYANNSK
VvCslE6 298 --GQPLPTLVYLSREKRSKYHHNFKAGAMNTLIRVSSR--ISNGEIILNVDCDMYSNNSE
AtCslA3 32 --ARKGINIMSEIRDNR----IGYKAGALKAGMMHN---YVKQCEFVAIFDADFQP-DPD
AtCslA7 18 --SKEGVNITFEIRDNR----NGYKAGALREGMRHS---YVKQCDYVAIFDADFQP-DPD
PbCCesA 165 -----RFGFNYSTRSTN----YMKKAGNLRDAFAKT------SAPFFVIFDADFCP-RKD
BaCesA 155 -----ELGVHYVTRPNN----EHAKAGNLNHAMSCS------NGELIVTMDADMVP-LPS
AtmCesA 199 -----DLDVRYLTRERN----VHAKAGNLNNGLAHS------TGELVTVFDADHAP-ARD
RlCesA 210 -----DLDVHYLTRDRN----EHAKAGNLNNGMKHS------TGELIAVFDADHAP-ARD
KxCesA3 201 -----ECGANYIARPTN----EHAKAGNLNYAIGHT------DGDYILIFDCDHVP-TRA
EcCesA 325 -----NVGVKYIARTTH----EHAKAGNINNALKYA------KGEFVSIFDCDHVP-TRS
PsCesA 597 -----QIGVGYLTRENN----YHAKAGNLNEALKST------DGEYIAMFDADHVP-TRS
PfCesA 211 -----KVGVNYIRRDNN----FHAKAGNLNEALKVT------DGEYIALFDADHVP-TRS
PvCesA3 673 YRDRGIPRVTFIGRMKPE--THHSKAGNINNALFN----EGADGKYLLILDNDMKP-HPK
PiCesA3 673 YRDRGIPRVTFIGRMKPE--THHSKAGNINNALFN----EGADGKYLLILDNDMKP-HPK
PvCesA4 466 Y--HGLPALTFVGRIKPR--VHHSKAGNINNVLYN----EGASGRYAIILDNDMKP-HEM
PiCesA4 466 Y--HGLPKLTFVGRIKPP--VHHSKAGNINNVLYN----EGACGRYAIILDNDMKP-HEM
PvCesA2 470 **Y**--**PGLPHVTFIGRVKPE**--**THYSKAGNINNCLYN**----**EGANGRYLIILDTDMQP**-**HPK**
PiCesA2 470 Y--PGLPHVTFIGRVKPE--THYSKAGNINNCLYN----EGANGRYLIILDTDMQP-HPK
PvCesA1 462 Y--PGLPHVTFIGRVKPE--THYYKAGNINNCLYN----EGSSGRYMLLLDSDVQP-HPK
PiCesA1 461 Y--PGLPHVTYVGRVKPE--THYSKAGNINNCLYN----EGANGRYMLLLDSDMQP-HPK
NsCesA 284 LELVELTRFRYIARPKPAGVPHHAKAGNLNYAIFS----GETSGEFILTLDADHIP-KPQ
TvCesA 256 LELVELARLRYIARPKPVGVPHHAKAGNLNYAIFS----GETSGELILTLDADHIP-KPQ
JfCesA 177 AFSTVRAPPIYIRRKKTPSVPHHAKAGNLNHALKC-----TPYSDFVAVFDADMQC-DPD
TlCesA 118 LLSHTYPNLYYMARTKIPGQPHHFKAGNLNYGLEQSHHLPGGAGEFMAALDADMIP-ERD
ThCesA 270 ILSHTYPNLYYMARAKIPGQPHHFKAGNLNYGLDQVQLLPGGASQFMAALDADMIP-ERD
GpCesA 145 LQKERNLNVNYIRRVKIKGVPHHYKAGNMNHALG------ITHGEYVGVLDSDMIT-APD
AmCesA 140 QA-VNGTNVRYIRRVKTPGVPHHAKAGNINHAMT------VTTGEFVGILDADMLV-RPE
AtCslD6 481 AFRDGICFMMD-H-----------------------------------------------
VvCslD3 629 ALREGMCYMMD-R-----------------------------------------------
CrCslD1 ------------------------------------------------------------
PpCslD8 660 AIREAMCFFMD-K-----------------------------------------------
AtCesA8 480 AVREAMCFLMD-P-----------------------------------------------
VvCesA8 482 AVREAMCFLMD-P-----------------------------------------------
VvCesA4 544 AAREAMCFLMD-P-----------------------------------------------
VvCesA5 521 ALRESMCFMMD-P-----------------------------------------------
AtCesA2 573 AIRESMCFMMD-P-----------------------------------------------
AtCesA6 572 ALREAMCFMMD-P-----------------------------------------------
StCesA1 490 ALREAMCFMMD-P-----------------------------------------------
HaCesA2 585 ALREAMCFMMD-P-----------------------------------------------
VvCesA6 558 ALREAMCFMMD-P-----------------------------------------------
VvCesA9 209 ALREAMCFMMD-P-----------------------------------------------
PbCesA 579 ALREAMCFLMD-P-----------------------------------------------
VvCesA7 589 AVREAMCFLMD-P-----------------------------------------------
StCesA7 550 AAREAMCFLMD-P-----------------------------------------------
VvCesA1 569 ALKEAMCFMMD-P-----------------------------------------------
ZmCesA2 566 ALREAMCFMMD-P-----------------------------------------------
VvCesA3 570 ALREAMCFLMD-P-----------------------------------------------
OsCesA2 565 ALREAMCFLMD-P-----------------------------------------------
AtCslB5 304 VVRQAMCVFLQNS-----------------------------------------------
StCslH1 301 VVLHAMCYFLG-A-----------------------------------------------
VvCslH1 305 IFHHSMCLLLG-S-----------------------------------------------
VvCslB3 341 IFHHAMCLLLG-S-----------------------------------------------
VvCslG2 279 SVRQAMCCHLD-P-----------------------------------------------
AtCslG3 334 TPVRALCYLTD-P-----------------------------------------------
AtCslE1 319 STRDALCILLD-E-----------------------------------------------
VvCslE6 354 SVRDALCFFMD-E-----------------------------------------------
AtCslA3 82 FLERTIPFL---------------------------------------------------
AtCslA7 68 FLHRTVPFL---------------------------------------------------
PbCCesA 209 FITEIMPYF---------------------------------------------------
BaCesA 199 FLQKTVGYF---------------------------------------------------
AtmCesA 243 FLLETVGYF---------------------------------------------------
RlCesA 254 FLLETVGYF---------------------------------------------------
KxCesA3 245 FLQLTMGWM---------------------------------------------------
EcCesA 369 FLQMTMGWF---------------------------------------------------
PsCesA 641 FLQVAMGWF---------------------------------------------------
PfCesA 255 FLQVSLGWF---------------------------------------------------
PvCesA3 726 FLLAVLPFFFSEGEAV--------------------------------------------
PiCesA3 726 FLLAVLPFFFSEGEAV--------------------------------------------
PvCesA4 517 FIQATLPFFFDVPQ--STKIMRCCAPGCGDSGKICCALCQAAGVPEAQIMFCSKDCYNAS
PiCesA4 517 FIQATLPFFFDAPQ--NSKITRCCAPGCGDIGKICCALCQAAGVPESQIMYCSKDCYNAS
PvCesA2 521 **FILATLPFFFDDEDRQDKAKYICCGVGCNAVAKLCCASCQIAGVPEEQISYCSKDCFENA**
PiCesA2 521 FILATLPFFFDDEDRQDKAKYICCGIGCNAVAKLCCASCQIAGVPEEQISYCSKDCFENA
PvCesA1 513 FILATLPFFFDDEDRQLKNKYSCSCLGCQNVAKMCCASCKIAGVPEERISYCSKECFENA
PiCesA1 512 FVLATLPFFFDDEDRQYKNKYSCSCMGCQNVAKMCCASCKIAGVPEERISYCSKECFENA
NsCesA 339 FLKRVLPYFYTYNL----------------------------------------------
TvCesA 311 FLKRVLPYFYTYNL----------------------------------------------
JfCesA 231 FLLETVSRL---------------------------------------------------
TlCesA 177 WLRALLPHA---------------------------------------------------
ThCesA 329 WLRALLPHA---------------------------------------------------
GpCesA 198 FLATLVPYL---------------------------------------------------
AmCesA 192 YLRRMLAQ----------------------------------------------------
AtCslD6 493 ------------------------------------------------------------
VvCslD3 641 ------------------------------------------------------------
CrCslD1 ------------------------------------------------------------
PpCslD8 672 ------------------------------------------------------------
AtCesA8 492 ------------------------------------------------------------
VvCesA8 494 ------------------------------------------------------------
VvCesA4 556 ------------------------------------------------------------
VvCesA5 533 ------------------------------------------------------------
AtCesA2 585 ------------------------------------------------------------
AtCesA6 584 ------------------------------------------------------------
StCesA1 502 ------------------------------------------------------------
HaCesA2 597 ------------------------------------------------------------
VvCesA6 570 ------------------------------------------------------------
VvCesA9 221 ------------------------------------------------------------
PbCesA 591 ------------------------------------------------------------
VvCesA7 601 ------------------------------------------------------------
StCesA7 562 ------------------------------------------------------------
VvCesA1 581 ------------------------------------------------------------
ZmCesA2 578 ------------------------------------------------------------
VvCesA3 582 ------------------------------------------------------------
OsCesA2 577 ------------------------------------------------------------
AtCslB5 317 ------------------------------------------------------------
StCslH1 313 ------------------------------------------------------------
VvCslH1 317 ------------------------------------------------------------
VvCslB3 353 ------------------------------------------------------------
VvCslG2 291 ------------------------------------------------------------
AtCslG3 346 ------------------------------------------------------------
AtCslE1 331 ------------------------------------------------------------
VvCslE6 366 ------------------------------------------------------------
AtCslA3 91 ------------------------------------------------------------
AtCslA7 77 ------------------------------------------------------------
PbCCesA 218 ------------------------------------------------------------
BaCesA 208 ------------------------------------------------------------
AtmCesA 252 ------------------------------------------------------------
RlCesA 263 ------------------------------------------------------------
KxCesA3 254 ------------------------------------------------------------
EcCesA 378 ------------------------------------------------------------
PsCesA 650 ------------------------------------------------------------
PfCesA 264 ------------------------------------------------------------
PvCesA3 742 ----------------------------------------------------------DG
PiCesA3 742 ----------------------------------------------------------DG
PvCesA4 575 GHVKSSVHRRQTQNTMSE------RVTCASCGSKINHKKGLCRKCNRAVSRRETNQFVGI
PiCesA4 575 GHVKSSVHRRQTQNTMSE------RMMCASCGSKINQKKGLCRKCNRAVSRRDSNQFVGV
PvCesA2 581 **MHVQSAVHRRQVNGTMSDTNASKIDMRCMNCDAKLP**-**KSGVCRKCGNKGADG**-**ED**---**VS**
PiCesA2 581 MHVQSAVHRRQVNGTMSDTRASKIDMRCMNCDAKLP-KNGVCRKCGNHGADG-ED---VS
PvCesA1 573 MHMQSDLHRRQVNGTLSDVHARKKELRCMNCNSKLG-KTGVCRKCNTNNHNGDAD---MT
PiCesA1 572 MHVQSDLHRRQVNGTLSDVRATKKELRCMNCDSKLG-KSGVCRKCNTNNNNGDAD---MS
NsCesA 353 ------------------------------------------------------------
TvCesA 325 ------------------------------------------------------------
JfCesA 240 ------------------------------------------------------------
TlCesA 186 ------------------------------------------------------------
ThCesA 338 ------------------------------------------------------------
GpCesA 207 ------------------------------------------------------------
AmCesA 200 ------------------------------------------------------------
AtCslD6 493 ---------DGDRVSYVQFPQRFEGID-------PSDRYANKNTVFFDINLRALDGIQGP
VvCslD3 641 ---------GGDRLCYVQFPQRFEGID-------PSDRYANRNTVFFDVNMRALDGLQGP
CrCslD1 ------------------------------------------------------------
PpCslD8 672 ---------GGDRLAYVQFPQRFEGVD-------PNDRYANHNTVFFDVNMRALDGLQGP
AtCesA8 492 --------VVGQDVCFVQFPQRFDGID-------KSDRYANRNIVFFDVNMRGLDGIQGP
VvCesA8 494 --------QVGQDVCYVQFPQRFDGID-------RSDRYANRNTVFFDVNMKGLDGIQGP
VvCesA4 556 --------QLGKKLCYVQFPQRFDGID-------LHDRYANRNVVFFDINMKGLDGIQGP
VvCesA5 533 --------LLGKRVCYVQFPQRFDGID-------KNDRYANRNTVFFDINMKGLDGIQGP
AtCesA2 585 --------QSGKKVCYVQFPQRFDGID-------RHDRYSNRNVVFFDINMKGLDGIQGP
AtCesA6 584 --------QSGKKICYVQFPQRFDGID-------RHDRYSNRNVVFFDINMKGLDGLQGP
StCesA1 502 --------TSGKKICYVQFPQRFDGID-------RHDRYSNRNVVFFDINMKGLDGIQGP
HaCesA2 597 --------TSGKKICYVQFPQRFDGID-------RHDRYSNRNVVFFDINMKGLDGIQGP
VvCesA6 570 --------TSGKKICYVQFPQRFDGID-------RNDRYSNRNVVFFDINMKGLDGIQGP
VvCesA9 221 --------ISGKKICYVQFPQRFDGID-------RNDRYSNRNVVFFDINMKGLDGIQGP
PbCesA 591 --------TVGKRVCYVQFPQRFDGID-------RNDRYANHNTVFFDINLKGLDGIQGP
VvCesA7 601 --------QTGRKVCYVQFPQRFDGID-------RNDRYANRNTVFFDINMKGLDGIQGP
StCesA7 562 --------QMGKKVCFVQFPQRFDGID-------KHDRYANRNTVFFDINMKGLDGIQGP
VvCesA1 581 --------AFGKKTCYVQFPQRFDGID-------LHDRYANRNIVFFDINLKGLDGVQGP
ZmCesA2 578 --------ALGRKTCYVQFPQRFDGID-------LHDRYANRNIVFFDINMKGLDGIQGP
VvCesA3 582 --------NLGKSVCYVQFPQRFDGID-------RNDRYANRNTVFFDINLRGLDGIQGP
OsCesA2 577 --------NLGRRVCYVQFPQRFDGID-------RNDRYANRNTVFFDINLRGLDGLQGP
AtCslB5 317 --------KNSNHCAFVQFPQKFYDS------------YTNELAVLQSILGRGVAGIQGP
StCslH1 313 --------KDEIDCGFVQFPQFFYDGL-------KEDPYGNQLKVLHEYFGRGISGIQGP
VvCslH1 317 --------KNEQDCGFVQTPQSFYDGL-------KDDPFGNQFGVLYKYVVSGIAGLQGP
VvCslB3 353 --------KNEQDCGFVQSPQCFYDGL-------KDDPFGNQLVVLYKYLGSGIAGLQGP
VvCslG2 291 --------KLSPSLAFVQFPQRFHNIS-------SNDIYDSQMRSAFSTLWEGMDGLDGP
AtCslG3 346 --------KIKTGLGFVQFPQTFQGIS-------KNDIYACAYKRLFEINMIGFDGLMGP
AtCslE1 331 --------KEGKEIAFVQFPQCFDNVT-------RNDLYGSMMRVGIDVEFLGLDGNGGP
VvCslE6 366 --------ESGHEIAYVQFPQCFNNIT-------KNDLYANS----LNVELAGFGSNGGP
AtCslA3 91 --------IHNHEISLVQCRWKFVNANECLM---TRMQEMSLNYHFVAEQESGSSIHAFF
AtCslA7 77 --------IHNPKLALVQGRWEFVNAGQCMM---TRLQEMSLSYHFTIEQQVGSSTFAFF
PbCCesA 218 --------NYDKSIAIVQTPQFFEVRPD-QT--WVERAAGSVQELFYRFIQVSRESFGAA
BaCesA 208 --------KKE-KVAFVQTPQAFYNEDPYQYNLFSGANIPNEQDFFMRRLQAGKDRFNAV
AtmCesA 252 --------EEDPRLFLVQTPHFFVNPDPIERNLRTFETMPSENEMFYGIIQRGLDKWNGA
RlCesA 263 --------EDDPKLFLVQTPHFFINPDPLERNLRTFDKMPSENEMFYGIIQRGLDKWNAA
KxCesA3 254 --------VEDPKIALMQTPHHFYSPDPFQRNLSAGYRTPPEGNLFYGVVQDGNDFWDAT
EcCesA 378 --------LKEKQLAMMQTPHHFFSPDPFERNLGRFRKTPNEGTLFYGLVQDGNDMWDAT
PsCesA 650 --------LKDPKLAMLQTPHFFFSPDPFEKNLDTFRSVPNEGELFYGLLQDGNDLWNAT
PfCesA 264 --------LKDPKLAMLQTPHFFFSPDPFEKNLDTFRAVPNEGELFYGLVQDGNDLWNAT
PvCesA3 744 GGRQYSDDISWNQVSYVQTPQYFEDTPQLTI---MGDPCGHKNTIFFDAVQCGRDGFDSA
PiCesA3 744 GGRQYSDDISWNQVSYVQTPQYFEDTPQLTI---MGDPCGHKNTIFFDAVQCGRDGFDSA
PvCesA4 629 SADDYSDHVSVNQVGYVQTPQYFEDCMQLR----LGDPCGHRNSTFFDSAQTGMDGYDCA
PiCesA4 629 SADDYSDHVSVNQVGYVQTPQYFEDCLQLR----LGDPCGHRNSTFFDSAQTGMDGYDCA
PvCesA2 636 **SLHTYSDDVRDNAVAFVQTPQYFRDCIQLQ**----**IGDPMGHRNATFYDAIQTGQDGYDCA**
PiCesA2 636 SLHNYSDDVRDNAVAFVQTPQYFRDCIQLQ----IGDPMGHRNATFYDAIQTGQDGYDCA
PvCesA1 629 ILHTYSDDVRDNAVGFVQTPQYFRDCVQLQ----IGDPLGHRNATFYDAIQTGQDGYDCA
PiCesA1 628 ILHTYSDDVRDNAVGFVQTPQYFRDCVQLQ----IGDPLGHRNSTFYDAIQTGQDGYDCA
NsCesA 353 ----FNGKYEQNRIAFVQTPQDFYNIP-------PSDPFGHRASLFYGPLQQGKDGMNAA
TvCesA 325 ----FNGKYEQNRIAFVQTPQDFYNIP-------PSDPFGHRASLFYGPLQQGKDGMNAA
JfCesA 240 --------CEDKQAAFVQTPQSFTNAEESS----ARDPLDTISRVYYNVILPGWAAWGCT
TlCesA 186 --------VQDPKMALVCPPQLFYNTP-------PSDPLAQSLDFFVHVIEPIKDAMGVA
ThCesA 338 --------VMDPKMALVCPPQLFYNTP-------PSDPLAQSLDFFVHVIEPIKDAMGVA
GpCesA 207 --------YERTDVAFVQSPQAYYNIP-------AGDPLAHYTTMFYDIIMPWRDGRDSA
AmCesA 200 --------FSSPRVSFVQCPQAYYNVP-------DGDPLGQVCAFFYDVVMPHRDTRNSA
AtCslD6 537 MYVGTGCLFRRTALYGFNPPDVFVVE-------EEPSGSY-CFPLIKKRSPATVAS----
VvCslD3 685 MYVGTGCLFRRTALYGFDPPRSKEHP-------GC-WS-C-CFGRG-KKKPASVANA---
CrCslD1 ------------------------------------------------------------
PpCslD8 716 VYVGTGCVFRRIALYGFDPPRIRDHG-------CC-FQIC-CFCCA-PKKPKMKKTK---
AtCesA8 537 VYVGTGTVFRRQALYGYSPPSKPRILPQSSSSS--------CCCLTKKKQPQDPSEI---
VvCesA8 539 VYVGTGCVFNRQALYGYGPPNLPNLPKASSSSSSCSWCGCCSCCCPSKKPSKDLSEV---
VvCesA4 601 VYVGTGCVFNRQALYGYDPPVSEK--RPKMTCD-C-WPSWCCCCCGGSRKSKSKKKVERG
VvCesA5 578 IYVGTGCVFRRQALYGYDAPKTKK--PPTRTCN-C-WPKWCCCG--GRKKKKKTNKP---
AtCesA2 630 IYVGTGCVFRRQALYGFDAPKKKK--PPGKTCN-C-WPKWCCLCCGLRKKSK--TK----
AtCesA6 629 IYVGTGCVFRRQALYGFDAPKKKK--GPRKTCN-C-WPKWCLLCFGSRKNRKAKTV----
StCesA1 547 IYVGTGCVFRRQALYGYDAPKKTK--PPGKTCN-C-WPKWCCCCFGSRKKHKKAKTT---
HaCesA2 642 IYVGTGCVFRRQALYGYDAPVKKK--PPGRTCN-C-LPRWCCCCCRSKKKNKKSKSK---
VvCesA6 615 IYVGTGCVFRRQALYGYDAPVNKK--PPGKTCN-C-WPKWCCLCCGSRKKNKKVKST---
VvCesA9 266 IYVGTGCVFRRQALYGYDAPVNKK--PPGKTCN-C-WPKWCCLCCGSRKKNKKVKST---
PbCesA 636 VYVGTGCVFKRQALYGYDPPPKDKISKRSHISG-I-CPTWC---CGPRMPRPKKPKS---
VvCesA7 646 VYVGTGCVFRRQALYGYDPPKGPKRPKMV-SCD-C-CPCF---------GRRKKLQK---
StCesA7 607 VYVGTGCVFRRQALYGYNPPKRAKRPRMV-SCD-C-CPCF---------GRKKKLDK---
VvCesA1 626 VYVGTGCCFNRQALYGYDPVLTEADLEPN-----I-IVKSC---CGSRKKGRGGNKK---
ZmCesA2 623 VYVGTGCCFNRQALYGYDPVLTEADLEPN-----I-VVKSC---CGRRKRK---NKS---
VvCesA3 627 VYVGTGCVFNRTALYGYEPPIKPKHKKPG-----V-FSLCC---GGSRKKGSK-SSK---
OsCesA2 622 VYVGTGCVFNRTALYGYEPPIKQ--KRPG-----Y-FSSLC---GGRKK-TKK-SKE---
AtCslB5 357 FYIGTGCFHTRRVMYGLSSDDLEDNGNIS------------------QV-----A-----
StCslH1 358 FYQGSGCFHRRKVIYGSSPYEKI-------------------------------------
VvCslH1 362 NYSGTGCFHRRKVIYGLWPDGRMEF-----------------------K-----G-----
VvCslB3 398 TYIGTGCFHRRKVIYGLWPDGRMEI-----------------------K-----G-----
VvCslG2 336 VLSGTGFYMKRVALYGTSIQGDTS------------------------------------
AtCslG3 391 NHVGTGCFFNRRGFYGAPSNLILP------------------------------------
AtCslE1 376 LYIGTGCFHRRDVICGRKYGEE--------------------------------------
VvCslE6 407 SYIGTGCFHRRETLCGKKYSEE--------------------------------------
AtCslA3 140 GFNGTAGVWRIAALNE--------------------------------------------
AtCslA7 126 GFNGTAGVWRISALNE--------------------------------------------
PbCCesA 267 VCVGTCAMYRREALVP--------------------------------------------
BaCesA 259 MYVGSNTVFRRSALEE--------------------------------------------
AtmCesA 304 FFCGSAAVLRREALQD--------------------------------------------
RlCesA 315 FFCGSAAVLSRKALES--------------------------------------------
KxCesA3 306 FFCGSCAILRRTAIEQ--------------------------------------------
EcCesA 430 FFCGSCAVIRRKPLDE--------------------------------------------
PsCesA 702 FFCGSCAVLRRSSLLD--------------------------------------------
PfCesA 316 FFCGSCAVIRREPLLE--------------------------------------------
PvCesA3 801 AFAGTNAVFRRQAFDS--------------------------------------------
PiCesA3 801 AFAGTNAVFRRQAFDS--------------------------------------------
PvCesA4 685 SFAGTNAIFRREALDS--------------------------------------------
PiCesA4 685 SFAGTNAIFRREALDS--------------------------------------------
PvCesA2 692 **SFAGTNAIFRREALDS**--------------------------------------------
PiCesA2 692 SFAGTNAMFRREALDS--------------------------------------------
PvCesA1 685 SFAGTNALVRRQALDS--------------------------------------------
PiCesA1 684 SFAGTNALIRREALDS--------------------------------------------
NsCesA 402 FYTGTNAILRREALINVGLQYFADDF----------------------------------
TvCesA 374 FYTGTNAILRREALINVGLQYFADDF----------------------------------
JfCesA 288 PCAGTNFLMRRAAIDS--------------------------------------------
TlCesA 231 WCTGSGYVARREALEE--------------------------------------------
ThCesA 383 WCTGSGYIARREALEQ--------------------------------------------
GpCesA 252 PCVGTGMIFRRKALED--------------------------------------------
AmCesA 245 PSVGTGVVFRRKALEE--------------------------------------------
AtCslD6 585 -----------------------------------EP-----------------------
VvCslD3 731 --------P-EE---E----D--------------ES-----------------------
CrCslD1 ------------------------------------------------------------
PpCslD8 763 --------TKQR---E----S--EV-------AGLTD-----------------------
AtCesA8 586 -----YKDAK-----RE--ELDAAIFNLGDLDN---------------------------
VvCesA8 596 -----YRDSK-----RD--DLNAAIFNLKEIDN---------------------------
VvCesA4 657 LLGGVYSKKKKMMGKNYSRKGSGPVFDLEEIEEGLEG-----------------------
VvCesA5 629 -----KSELKKRNSRKADAGGHVPVCALEGIEEGIE------------------------
AtCesA2 680 ------AKDKKTNTKE----TSKQIHALENVDEGVIV-----------------------
AtCesA6 681 ------AADKKKKNRE----ASKQIHALENIEEGRVT-----------------------
StCesA1 600 -----KDNKKKPKSKE----ASPQIHALENIEEGIE------------------------
HaCesA2 695 ------SHE-KKKSKE----ASKQIHALENIEEGIE------------------------
VvCesA6 668 ------DKKKKMKNRE----ASKQIHALENIEEGIE------------------------
VvCesA9 319 ------DKKKKMKNRE----ASKQIHALENIEEGIE------------------------
PbCesA 688 -----KSSGKLKCSARL--DSAVPIFSLEDMGERIE------------------------
VvCesA7 691 -----------------------YAKH----GENGE------------------------
StCesA7 652 -----------------------YKSEVNGDAANAQ------------------------
VvCesA1 674 -----YID-KKRQVKRT--ESTIPIFNMEDIEEGVE------------------------
ZmCesA2 668 -----YMDSQSRIMKRT--ESSAPIFNMEDIEEGIE------------------------
VvCesA3 674 -----KGSDKKKSSKHV--DPTVPIFNLEDIEEGVEGTVLIFLFGNIYFTGCGTHHPLLI
OsCesA2 666 -----KSTEKKKSHKHV--DSSVPVFNLEDIEEGIEG-----------------------
AtCslB5 389 ------------------------------------------------------------
StCslH1 381 ------------------------------------------------------------
VvCslH1 389 ------------------------------------------------------------
VvCslB3 425 ------------------------------------------------------------
VvCslG2 360 ------------------------------------------------------------
AtCslG3 415 ------------------------------------------------------------
AtCslE1 398 ------------------------------------------------------------
VvCslE6 429 ------------------------------------------------------------
AtCslA3 156 ------------------------------------------------------------
AtCslA7 142 ------------------------------------------------------------
PbCCesA 283 ------------------------------------------------------------
BaCesA 275 ------------------------------------------------------------
AtmCesA 320 ------------------------------------------------------------
RlCesA 331 ------------------------------------------------------------
KxCesA3 322 ------------------------------------------------------------
EcCesA 446 ------------------------------------------------------------
PsCesA 718 ------------------------------------------------------------
PfCesA 332 ------------------------------------------------------------
PvCesA3 817 ------------------------------------------------------------
PiCesA3 817 ------------------------------------------------------------
PvCesA4 701 ------------------------------------------------------------
PiCesA4 701 ------------------------------------------------------------
PvCesA2 708 ------------------------------------------------------------
PiCesA2 708 ------------------------------------------------------------
PvCesA1 701 ------------------------------------------------------------
PiCesA1 700 ------------------------------------------------------------
NsCesA 428 ------------------------------------------------------------
TvCesA 400 ------------------------------------------------------------
JfCesA 304 ------------------------------------------------------------
TlCesA 247 ------------------------------------------------------------
ThCesA 399 ------------------------------------------------------------
GpCesA 268 ------------------------------------------------------------
AmCesA 261 ------------------------------------------------------------
AtCslD6 587 -----EYYTDEEDRFDIGLIRKQFGSSSMLVNSVKVAEFEGRPLATVHSSRLGRPPGSLT
VvCslD3 738 -----HGLRETDDEMNSSLLPKSFGNSSFLIDSIPVAEFQGRPLADHPSVKNGRQPGALT
CrCslD1 ------------------------------------------------------------
PpCslD8 776 -----HTTSDDDDEIEASMLPKRYGSSAVFAASIPVAEFQGRPLADK-GVHNGRPAGALT
AtCesA8 607 -----YDEYDRSMLISQTSFEKTFGLSTVFIESTLMEN-GGV------------P-----
VvCesA8 617 -----YDEHERSLLISQMSFEKTFGLSSVFIESTLMEN-GGV------------P-----
VvCesA4 694 -----YDELEKSSLMSQKNFEKRFGQSPVFITSTLMED-GGL------------P-----
VvCesA5 660 -----GIESENVALMSEQKLEKKFGQSPVFVASTLLEN-GGT------------L-----
AtCesA2 707 ----PVSNVEKRSEATQLKLEKKFGQSPVFVASAVLQN-GGV------------P-----
AtCesA6 708 ----KGSNVEQSTEAMQMKLEKKFGQSPVFVASARMEN-GGM------------A-----
StCesA1 627 -----GIDSEKAALMPQIKLEKKFGQSPVFVASTLLED-GGI------------P-----
HaCesA2 720 -----GIDNEKSALMPQIKFEKKFGQSSVFIAATLMED-GGV------------P-----
VvCesA6 694 -----GIDNDRSLLMPQVKFEKKFGQSPVFIASTLLEE-GGV------------P-----
VvCesA9 345 -----GIDNDRSLLMPQVKFEKKFGQSPVFIASTLLEE-GGV------------P-----
PbCesA 717 -----GMEDEKSSLMSLQNFEKRFGQSPVFVASTLLED-GGV------------P-----
VvCesA7 700 -----GLEEDKEMLMSQMNFEKKFGQSAIFVTSTLMEQ-GGV------------P-----
StCesA7 665 -----GFDDDNELLMSQMNFEKKFGQSAIFVTSTLMIE-GGV------------P-----
VvCesA1 702 -----GYDDEKSLLMSQKSLEKRFGQSPVFIAATFMEQ-GGI------------P-----
ZmCesA2 697 -----GYEDERSVLMSQRKLEKRFGQSPIFIASTFMTQ-GGI------------P-----
VvCesA3 727 FKIGAGFDDEKSLLMSQMSLEKRFGQSAVFVASTLMEN-GGV------------P-----
OsCesA2 696 ----SGFDDEKSLLMSQMSLEKRFGQSSVFVASTLMEY-GGV------------P-----
AtCslB5 389 ----------TREFLAEDSLVRKYGNSKELVKSVVDALQRKS--------------N---
StCslH1 381 ----------TAGELKDEYIQKTYGMSEKLSTSIAKTLLEGS--------------NIIE
VvCslH1 389 ----------RI----DERLEKTFGNSKEFTKTAARILSGLS--------------G-IS
VvCslB3 425 ----------RSGKLTDERIQKTFGNSKEFTKTAARILSGLS--------------G-IS
VvCslG2 360 ----------------LTELRQTFGYSDEFI-----------------------------
AtCslG3 415 ----------------EIDELKPNRIVDKPIN----------------------------
AtCslE1 398 -----------------EEEEESERIH---------------------------------
VvCslE6 429 -----------------CEREQTTRNNNERIE----------------------------
AtCslA3 156 ------------------------------------------------------------
AtCslA7 142 ------------------------------------------------------------
PbCCesA 283 ------------------------------------------------------------
BaCesA 275 ------------------------------------------------------------
AtmCesA 320 ------------------------------------------------------------
RlCesA 331 ------------------------------------------------------------
KxCesA3 322 ------------------------------------------------------------
EcCesA 446 ------------------------------------------------------------
PsCesA 718 ------------------------------------------------------------
PfCesA 332 ------------------------------------------------------------
PvCesA3 817 ------------------------------------------------------------
PiCesA3 817 ------------------------------------------------------------
PvCesA4 701 ------------------------------------------------------------
PiCesA4 701 ------------------------------------------------------------
PvCesA2 708 ------------------------------------------------------------
PiCesA2 708 ------------------------------------------------------------
PvCesA1 701 ------------------------------------------------------------
PiCesA1 700 ------------------------------------------------------------
NsCesA 428 -----------------TKDEKR-------------------------------------
TvCesA 400 -----------------TKDEKR-------------------------------------
JfCesA 304 ------------------------------------------------------------
TlCesA 247 ------------------------------------------------------------
ThCesA 399 ------------------------------------------------------------
GpCesA 268 ------------------------------------------------------------
AmCesA 261 ------------------------------------------------------------
AtCslD6 642 GSRKPLDFATVNEAVNVISCWYEDKTEWGFNVGWIYGSVTEDVVTGFRMHEKGWRSFYCV
VvCslD3 793 ISREPLGAATVAEAISVISCWYEDKTEWGQRVGWIYGSVTEDVVTGYRMHNRGWRSIYCV
CrCslD1 ------------------------------------------------------------
PpCslD8 830 IPREPLDASTVAEAINVVSCFYEDKTEWGGRVGWIYGSVTEDVVTGFRMHNRGWRSIYCV
AtCesA8 644 --DSVNPSTLIKEAIHVISCGYEEKTEWGKEIGWIYGSITEDILTGFKMHCRGWRSIYCM
VvCesA8 654 --ESANSPILIKEAIHVISCGYEEKTEWGKEIGWIYGSVTEDILTGFKMHCRGWRSLYCM
VvCesA4 731 --EGTNSTALIKEAIHVISCGYEEKTEWGKEIGWIYGSVTEDILTGFKMHCRGWKSVYCM
VvCesA5 697 --KSASPASLLKEAIHVISCGYEDKTEWGKEVGWIYGSVTEDILTGFKMHCHGWRSIYCI
AtCesA2 745 --RNASPACLLREAIQVISCGYEDKTEWGKEIGWIYGSVTEDILTGFKMHCHGWRSVYCM
AtCesA6 746 --RNASPACLLKEAIQVISCGYEDKTEWGKEIGWIYGSVTEDILTGFKMHSHGWRSVYCT
StCesA1 664 --PGATSASLLKEAIHVISCGYEDKTEWGKEIGWIYGSVTEDILTGFKMHCHGWRSVYCM
HaCesA2 757 --KGASSASLLKEAIHVISCGYEDKTEWGKEIGWIYGSVTEDILTGFKMHCHGWRSVYCT
VvCesA6 731 --KGATTASLLKEAIHVISCGYEDKTEWGKEVGWIYGSVTEDILTGFKMQCHGWRSVYCI
VvCesA9 382 --KGATTASLLKEAIHVISCGYEDKTEWGKEVGWIYGSVTEDILTGFKMQCHGWRSVYCI
PbCesA 754 --HTANPGSLLKEAIHVISCGYEDKTEWGKEIGWIYGSVTEDILTGFKMHCRGWRSIYCM
VvCesA7 737 --PSSSPAALLKEAIHVISCGYEDKTDWGLELGWIYGSITEDILTGFKMHCRGWRSIYCM
StCesA7 702 --PSSSPAALLKEAIHVISCGYEDKTEWGLELGWIYGSITEDILTGFKMHCRGWRSVYCM
VvCesA1 739 --PSTNPATLLKEAIHVISCGYEDKTDWGKEIGWIYGSVTEDILTGFKMHARGWISIYCM
ZmCesA2 734 --PSTNPASLLKEAIHVISCGYEDKTEWGKEIGWIYGSVTEDILTGFKMHARGWQSIYCM
VvCesA3 769 --QSAAPETLLKEAIHVISCGYEDKSEWGREIGWIYGSVTEDILTGFKMHARGWRSIYCM
OsCesA2 734 --QSATPESLLKEAIHVISCGYEDKSDWGTEIGWIYGSVTEDILTGFKMHARGWRSIYCM
AtCslB5 422 --PQKSLANLIEAAQEVGHCHYEYQTSWGN-LGWMYDSVAEDINTSVGIHLRGWTSSFIS
StCslH1 417 QFNSDSPSSSIEIAHQVGSCGFEFGTAWGQKLGWLYGSVAEDILTGLFIQSRGWKSAYCL
VvCslH1 420 DCP-YDLSNRVEAAHQIASCSYEYGTNWGTKIGWLYGTTTEDILTGMRIHARGWKSTDCR
VvCslB3 460 HCP-YDLLNRVEAAQEVATCSYEYGTSWGTKVSCKYLEQQE-------------------
VvCslG2 375 -----------------------------KSLSPKYLPNIS---------------NGGD
AtCslG3 431 ------AQDVLALAHRVAGCIYELNTNWGSKIGFRYGSLVEDYYTGYRLHCEGWRSVFCR
AtCslE1 408 ----E--NLEPEMIKALASCTYEENTQWGKEMGVKYGCPVEDVITGLTIQCRGWKSAYLN
VvCslE6 444 ----ENASVLEETCKVLASCSYEDYTQWGKEMGLKYGCPVEDTLTGLSIQCRGWKSIYFT
AtCslA3 156 ------------------------------AGGWKDRTTVEDMDLAVRACLHGWKFVYVH
AtCslA7 142 ------------------------------SGGWNDQTTVEDMDLAVRATLRGWKFLYID
PbCCesA 283 ------------------------------FGGTAEIGFSEDVHTGFSVVDAGWKLKYVP
BaCesA 275 ------------------------------IGGFATGVITEDMATGMLLQ-TKFKSVFVK
AtmCesA 320 ------------------------------TEGFSGVSITEDCETALALHSRGWNSIYVD
RlCesA 331 ------------------------------QNGFSGISITEDCETALALHGSGWNSIYVD
KxCesA3 322 ------------------------------IGGFATQTVTEDAHTALKMQRLGWSTAYLR
EcCesA 446 ------------------------------IGGIAVETVTEDAHTSLRLHRRGYTSAYMR
PsCesA 718 ------------------------------IGGVATETVTEDAHTALKLNRAGYNTAYLA
PfCesA 332 ------------------------------IGGVAVETVTEDAHTALKLNRLGYNTAYLA
PvCesA3 817 ------------------------------IGGICYGTQTEDAYTGNVLHTSGWDSVYFR
PiCesA3 817 ------------------------------IGGICYGTQTEDAYTGNVLHTSGWDSVYFR
PvCesA4 701 ------------------------------VCGIQYGSLTEDAYTGKMMVDKGWKGYYFR
PiCesA4 701 ------------------------------VCGIQYGSLTEDAYTGKMMVDKGWKGYYFR
PvCesA2 708 ------------------------------**IGGIQYGSLTEDCYTGQVLCSMGWKAQYFR**
PiCesA2 708 ------------------------------IGGIQYGSLTEDCYTGQVLCSMGWKAQYFR
PvCesA1 701 ------------------------------IGGIQYGTLTEDCYTGERLVSMGWKALYFR
PiCesA1 700 ------------------------------IGGIQYGTLTEDCYTGERLVSMGWKALYFR
NsCesA 434 ------------------------LDEFQLVGGLSSNSITEDMNTAMRLHGAGWKSVYHH
TvCesA 406 ------------------------LDEFQLVGGLSSNSITEDMNTAMRLHGAGWKSVYHH
JfCesA 304 ------------------------------IGGFPTGCVTEDYLMSMKLHGKGWTSLYLN
TlCesA 247 ------------------------------IGNFPLGSLAEDVATSTMMLGKGWKTAYVH
ThCesA 399 ------------------------------IGNFPLGSLAEDVATSTLMLGKGWKTAYVH
GpCesA 268 ------------------------------IGGFSIGTITEDFDTAMACQNRGWKTIYVN
AmCesA 261 ------------------------------VGGMSTGTLTEDFDTSIKLMGRGWHTVYIN
AtCslD6 702 TE--------PDAFRGSAPINLTDRLHQVLRWATGSVEIFFSRN------------NAIF
VvCslD3 853 TK--------RDAFRGTAPINLTDRLHQVLRWATGSVEIFFSRN------------NALL
CrCslD1 ------------------------------------------------------------
PpCslD8 890 TK--------RDAFRGTAPINLTDRLHQVLRWATGSVEIFFSRN------------NALL
AtCesA8 702 PL--------RPAFKGSAPINLSDRLHQVLRWALGSVEIFLSRH------------CPLW
VvCesA8 712 PL--------RPAFKGSAPINLSDRLHQVLRWALGSVEIFLSRH------------CPLW
VvCesA4 789 PK--------RAAFKGSAPINLSDRLHQVLRWALGSVEIFLSRH------------CPLW
VvCesA5 755 PS--------RPAFKGSAPINLSDRLHQVLRWALGSIEIFLSRH------------CPLW
AtCesA2 803 PK--------RAAFKGSAPINLSDRLHQVLRWALGSVEIFLSRH------------CPIW
AtCesA6 804 PK--------LAAFKGSAPINLSDRLHQVLRWALGSVEIFLSRH------------CPIW
StCesA1 722 PD--------RPAFKGSAPINLSDRLHQVLRWALGSVEIFFSRH------------CPIW
HaCesA2 815 PK--------IPAFKGSAPINLSDRLHQVLRWALGSVEILLSRH------------CPIW
VvCesA6 789 PK--------RPAFKGSAPINLSDRLHQVLRWALGSVEIFFSRY------------CPIW
VvCesA9 440 PK--------RPAFKGSAPINLSDRLHQVLRWALGSVEIFFSRY------------CPIW
PbCesA 812 PP--------RPAFKGSAPINLSDRLNQVLRWALGSVEICLSRH------------CPIW
VvCesA7 795 PK--------RPAFKGSAPINLSDRLNQVLRWALGSVEIFFSRH------------SPVW
StCesA7 760 PK--------LAAFKGSAPINLSDRLNQVLRWALGSVEIFFSHH------------SPVW
VvCesA1 797 PP--------RPAFKGSAPINLSDRLNQVLRWALGSIEILLSRH------------CPIW
ZmCesA2 792 PP--------RPCFKGSAPINLSDRLNQVLRWALGSVEILLSRH------------CPIW
VvCesA3 827 PK--------RPAFKGSAPINLSDRLNQVLRWALGSVEILFSRH------------CPIW
OsCesA2 792 PK--------RPAFKGSAPINLSDRLNQVLRWALGSVEILFSRH------------CPIW
AtCslB5 479 PD--------PPAFIGSTPTLGLEAIVQQRRWATGAIEVLFNKQ------------SPFM
StCslH1 477 PD--------PPAFLGCAPAAGPASMIQQKRWATGLFEVLLNSK------------SPII
VvCslH1 479 PD--------PPAFLGCAPSGGPAALIQQKRWATGLLEVLFSKN------------SPFI
VvCslB3 ------------------------------------------------------------
VvCslG2 391 SL--------SAQFVGSSVTNLNDLLVQGTRWSSGLVDVGISKF------------CPFI
AtCslG3 485 PK--------RAAFCGDSPKSLIDVVSQQKRWAIGLLEVAISRY------------SPIT
AtCslE1 462 PE--------KQAFLGVAPTNLHQMLVQQRRWSEGDFQIMLSKY------------SPVW
VvCslE6 500 PE--------RKAFLGVAPTTLLQSLIQHKRWAEGDFQIFLSSY------------CPFT
AtCslA3 186 D----------VEVKNELPSTFKAYRFQQHRWSCGPANLWRKMT------------MEIL
AtCslA7 172 D----------LKVKSELPCSFKALRSQQHRWTCGPANLLRKMA------------GQII
PbCCesA 313 L----------NLAKGVCPYELKSFFSQQYRWALGSTTL-CFNP------------HFWK
BaCesA 304 E----------VLAVGLSPETWTDLLKQRDRWCRGNIQCGKKWN------------PLFL
AtmCesA 350 K----------PLIAGLQPATFASFIGQRSRWAQGMMQILIFRQ------------PLFR
RlCesA 361 K----------PLIAGLQPATFASFIGQRSRWAQGMMQILRFRF------------PLLK
KxCesA3 352 I----------PLAGGLATERLILHIGQRVRWARGMLQIFRIDN------------PLFG
EcCesA 476 I----------PQAAGLATESLSAHIGQRIRWARGMVQIFRLDN------------PLTG
PsCesA 748 I----------PQAAGLATESLSRHVAQRIRWARGMAQIFRTDN------------PLLG
PfCesA 362 I----------PQAAGLATESLSRHINQRIRWARGMAQIFRTDN------------PLLG
PvCesA3 847 KDFEGDAKDRIRLCEGAVPETVAAAMGQKKRWAKGAVQILLMK-NESEVDPDWRPPRVPA
PiCesA3 847 KDFEGDAKDRIRLCEGAVPDTVAAAMGQKKRWAKGAVQILLMK-NESEVDPDWRPPRVPA
PvCesA4 731 KDLEGEEADRIRLAEGAVPESVAAALAQRKRWAKGNFQIFLRN-KKSLVDPLWQPPQVEL
PiCesA4 731 KDLEGEEADRIRLAEGAVPESVAAALAQRKRWAKGNFQIFLRN-KKSLVDPEWATPQVEL
PvCesA2 738 **KDFEGEPSERIRLAEGLIPDSVAGSLAQRKRWAKGNFQIALMNKKTQYFDPEW**KMPEVQV
PiCesA2 738 KDFEGEPSERIRLAEGLIPDSVAGSLAQRKRWAKGNFQIALMNKKTQYFDPEWKMPEAQI
PvCesA1 731 KDFEGETEERVRLAEGKIPDSVAGAMAQRKRWAKGNFQTALMKKSKNVADPEWKRPHVDI
PiCesA1 730 KDFEGEAEERIRLAEGLIPDSVAGAMAQRKRWAKGNFQTALMKKNKNVADPEWKRPHVDI
NsCesA 470 E----------LLAEGLAPDDLSSTLKQRLRWAQGTIQVLVKEN------------PLRK
TvCesA 442 E----------LLAEGLAPDDLSSTLKQRLRWAQGTIQVLVREN------------PLSK
JfCesA 334 K----------VLARGLAPENIKDLFRQRSRWAKGNLQIWFNHN------------PIIE
TlCesA 277 E----------PLQFGTVPEDYGSHLKQRTRWAIGTVDTSFKLN------------FCLW
ThCesA 429 E----------PLQFGTVPEDYGSHLKQRTRWAIGTVDTSFKLN------------FCLW
GpCesA 298 K----------KMQFGLVPDTLDATLKQRERWGVGTLQIFFKRN------------PLLM
AmCesA 291 D----------KLQYGLVPTDLRSALRQRERWAIGTLEILWKRN------------PLTA
AtCslD6 742 AG------PKLKLLQRIAYLNVGIYPFTSIFILTYCFL------PPLSLFSGHFVVE---
VvCslD3 893 AS------HRMKFLQKIAYMNVGIYPFTSIFLVVYCFL------PALSLFSGEFIVQ---
CrCslD1 ------------------------------------------------------------
PpCslD8 930 AS------SRLKFLQRIAYLNVGIYPFTSIFLLVYCFL------PALSLYTGQFIVQ---
AtCesA8 742 YGCSGG---RLKLLQRLAYINTIVYPFTSLPLVAYCTL------PAICLLTGKFIIP---
VvCesA8 752 YGFGGG---RLKWLQRMAYINTIVYPFTSLPLIAYCSL------PAICLLTGKFIIP---
VvCesA4 829 YGYGG----KLKWLERLAYINTIVYPFTSIPLLAYCTI------PAVCLLTGKFIIP---
VvCesA5 795 YGYGG----GLKWLERLSYINATVYPWTSIPLLAYCTL------PAVCLLTGKFITP---
AtCesA2 843 YGYGG----GLKWLERFSYINSVVYPWTSLPLIVYCSL------PAVCLLTGKFIVP---
AtCesA6 844 YGYGG----GLKWLERLSYINSVVYPWTSLPLIVYCSL------PAICLLTGKFIVP---
StCesA1 762 YGYGC----GLKPLERFSYINSVVYPLTSIPLIIYCTL------PAVFLLTRKFNWF---
HaCesA2 855 YGYGC----GLKWLERFSYINSVVYPLTSIPLIAYCTL------PAVCLLTGKFIVP---
VvCesA6 829 YGYGG----GLKWLERFSYINSVVYPWTSIPLIAYCTL------PAFCLLTGKFIVP---
VvCesA9 480 YGYGG----GLKWLERFSYINSVVYPWTSIPLIAYCTL------PAFCLLTGSSLSPRQG
PbCesA 852 YGYGGGKSGGLKCLERLAYINTTVYPLTSLPLLAYCVL------PAVCLLTGKFIIP---
VvCesA7 835 YGYKGG---NLKWLERFAYVNTTVYPFTSLPLLAYCTL------PAICLLTGKFIMP---
StCesA7 800 YGHKGG---NLKWLERLSYINTTIYPFTSLPLLAYCTL------PAVCLLTGKFIMP---
VvCesA1 837 YGYNG----RLKLLERLAYINTIVYPLTSIPLIAYCVL------PAICLLTGKFIIP---
ZmCesA2 832 YGYNG----RLKLLERLAYINTIVYPITSVPLIAYCVL------PAICLLTNKFIIP---
VvCesA3 867 YGYGG----RLKWLERFAYVNTTIYPITAIPLLVYCTL------PAVCLLTGKFIIP---
OsCesA2 832 YGYGG----RLKFLERFAYINTTIYPLTSIPLLLYCIL------PAICLLTGKFIIP---
AtCslB5 519 GMFHG----KIKFRQRLAYFWAL-MCLRSIPELIYCLL------PAYCLLHDSALFP---
StCslH1 517 GTLFG----KLQLRQCMAYLQIQLWGLRSIFEVCYAIL------PAYCLITNSHFLP---
VvCslH1 519 VTFTA----KLQFRQCLAYMWIISWGLRPIPELCYLAL------PAYCIMAGSHFLP---
VvCslB3 ------------------------------------------------------------
VvCslG2 431 YG-PL----KTSFLENICYSELSFFPFYFLPVWCFGTI------PQLCLFHGVPLYP---
AtCslG3 525 YG-VK----SMGLVTGVGYCQYACWAFWSLPLIVYGFL------PQLALLYQSSVFP---
AtCslE1 502 YG-KG----KISLGLILGYCCYCLWAPSSLPVLIYSVL------TSLCLFKGIPLFP---
VvCslE6 540 YG-HK----MIPLKLQISYCLFVLLAPNCLPTLYYVAI------PSLCLLKGISLFP---
AtCslA3 224 --QNK----KVSAW-KKLYLIYNFFFIRKIVVHIFTFVFYCLILPTTVLFPELQV---PK
AtCslA7 210 --RSE----NVSLW-KKWYMLYSFFFMRKIVAHILTFCFYCVILPATVLFPEVTV---PK
PbCCesA 350 ----S----ALSLRQKVCFLSGMLYFQTTAVATILSSI------PGIIML------KNFP
BaCesA 342 ----K----GLTPMQRILYFDGILFWFFGVFKMVYILA------PLLFLLFGIHSLKTDL
AtmCesA 388 ----R----GLSFTQRLCYMSSTLFWLFPFPRTIFLFA------PLFYLFFDLQIFVASG
RlCesA 399 ----R----GLTIPQRFCYMSSTLFWLFPFPRTIFLFA------PLFYLFFDLEIFTASG
KxCesA3 390 ----R----GLSWGQRLCYLSAMTSFLFAVPRVIFLSS------PLAFLFFGQNIIAASP
EcCesA 514 ----K----GLKFAQRLCYVNAMFHFLSGIPRLIFLTA------PLAFLLLHAYIIYAPA
PsCesA 786 ----K----GLSLGQRLCYANSMLHFFYGLPRLVFLTA------PLAYLLFGAEVMHASA
PfCesA 400 ----K----GLKWGQRICYANAMLHFFYGLPRLVFLTA------PLAYLIFGAEIFHASA
PvCesA3 906 PDPKP----SLTFPRKMFFYDSVLYPFGSIPALCYVSI------AVYYLCTGDAPIYARG
PiCesA3 906 PDPKP----SLTFPRKMFFYDSVLYPLGSIPALCYVSI------AVYYLCTGDAPIYARG
PvCesA4 790 PPKRK----INKFMRWVFFMNLTVYPIGSFPAIFFFYV------TAYFLYTGQAPIYTSG
PiCesA4 790 PPKRK----INKFMRWVFFMNLTVYPIGSFPAIFFFYI------TGYFLYTGQAPIYTSG
PvCesA2 798 PTYHK----SNKFMRRVFYFNSTLYPLGSITAILFYYI------TIYFLFSGYAPIYMAG
PiCesA2 798 PSYHK----SNKFMRRVFYFNSTLYPLGSITAILFYYI------TLYFLFSGYAPIYMAG
PvCesA1 791 PKYRK----PNTFMRRVFYLNSTLYPIDSISVILVYYV------TLYFLYTGFAPIYVNG
PiCesA1 790 PKYRK----PNNFMRRVFYLNSTLYPIDSIPVILLYYI------TLYFLYTGYAPIFVNG
NsCesA 508 SG--------LTFWQRLQYFKTMYSYFSGFATLIFISC------PIIYFFTEIVPVKTYG
TvCesA 480 PG--------LTFWQRLQYFKTMYSYFSGFATLIFISC------PIIYFFTEISPVKTYG
JfCesA 372 PG--------LTPIQRLLYMSVGVGYLNSALLVFWMVI------PIFHLITGIL--PIF-
TlCesA 315 GEKVR----KMTFAQRFS-------GFLYASLSLYTIL------LSIS-LFAIPVILIMG
ThCesA 467 GEKVR----QMTFAQRFS-------GFLYASLSLYTIL------LSIS-LFAIPIILIMG
GpCesA 336 KG-------QLKFHQRVMYFSAGMSYLLPVAILAFTLL------PFLTIVFDWPVVPVR-
AmCesA 329 KG--------LSFHQRVMFFSCGLSYIIPVGIVIFAVL------PVLTLFWQFPIMPVM-
AtCslD6 787 --------------------------TLTGSFL-IYL-LIITLSLCGLAVLEV-------
VvCslD3 938 --------------------------SLSVAFL-TYL-LGITITLCLLAVLEI-------
CrCslD1 ------------------------------------------------------------
PpCslD8 975 --------------------------NLNLAFL-IYL-LTITISLCSLAVLEV-------
AtCesA8 790 --------------------------TLSNLAS-MLF-LGLFISIILTSVLEL-------
VvCesA8 800 --------------------------TLSNLAS-VWF-LGLFISIILTSVLEL-------
VvCesA4 876 --------------------------TLTNFAS-VWF-MALFLSIIV-------------
VvCesA5 842 --------------------------ELSNVAS-LWF-LSLFICIFATGILEM-------
AtCesA2 890 --------------------------EISNYAG-ILF-MLMFISIAVTGILEM-------
AtCesA6 891 --------------------------EISNYAS-ILF-MALFSSIAITGILEM-------
StCesA1 809 -------------------------PEISNYAS-ILF-MGLFIMIAVTSVIEM-------
HaCesA2 902 --------------------------EISNYAS-IIF-MALFISIAATGILEM-------
VvCesA6 876 --------------------------EISNYAS-IIF-MALFISIAATGVLEM-------
VvCesA9 530 FPLENGTFDISCQVQSPANPNFLHDVQISNYAS-IIF-MALFISIAATGVLEM-------
PbCesA 903 --------------------------SISNLAS-LWF-ISLFISIFATGILEM-------
VvCesA7 883 --------------------------TISTFAS-LFF-IALFISIFATGILEL-------
StCesA7 848 --------------------------EISTLAS-LFF-IALFLSIFTTGILEL-------
VvCesA1 884 --------------------------EISNFAS-MWF-ILLFVSIFATGILEL-------
ZmCesA2 879 --------------------------EISNYAG-MFF-ILLFASIFATGILEL-------
VvCesA3 914 --------------------------QISNIAS-IWF-ISLFLSIFATGILEM-------
OsCesA2 879 --------------------------EISNFAS-IWF-ISLFLSIFATGILEM-------
AtCslB5 565 --------------------------KGPCLCT-IV---TLVGMHCLYSLWQF-------
StCslH1 564 --------------------------QANELSI-VIP-ASIFIIYNLYVLSEY-------
VvCslH1 566 --------------------------NVQDPAV-LIP-ISLFVSYNFHTLLEY-------
VvCslB3 ------------------------------------------------------------
VvCslG2 477 --------------------------EVSNSFF-GVF-PFIFLSACSKHLLEV-------
AtCslG3 571 --------------------------KSSDPWF-WLY-IVLFLGAYGQDLLDF-------
AtCslE1 548 --------------------------KVSSSWF-IPF-GYVTVAATAYSLAEF-------
VvCslE6 586 --------------------------KISSLWI-LPF-AHVISSSCAYSL----------
AtCslA3 274 ------------------------------W------ATVYFPTTITILNAIA-------
AtCslA7 260 ------------------------------W------AAFYLPSLITLLIAIG-------
PbCCesA 390 ------------------------------DHILVFNITFAIPSILFGYILMP-------
BaCesA 388 ------------------------------WSIT----SFWLPAFLGSYLSFK-------
AtmCesA 434 ------------------------------GEFL----AYTAAYMLVNLMMQN-------
RlCesA 445 ------------------------------GEFL----AYTLAYMLVNLMMQN-------
KxCesA3 436 ------------------------------LALL----AYAIPHMFHAVGTAS-------
EcCesA 560 ------------------------------LMIA----LFVLPHMIHASLTNS-------
PsCesA 832 ------------------------------LMIT----AYVLPHLAHASLTNS-------
PfCesA 446 ------------------------------LMIV----AYVLPHLVHSSLTNS-------
PvCesA3 956 ------------------------------TK----FLYSFLPVTFCRWVLNL---LANR
PiCesA3 956 ------------------------------TK----FLYSFLPVTFCRWVLNL---LANR
PvCesA4 840 ------------------------------LR----LLMALVPKIVAQSILSA---LSNR
PiCesA4 840 ------------------------------LR----LLMALVPKIVAQSILSA---LSNR
PvCesA2 848 ------------------------------ER----LVYALVPKLLVQGLLSA---LSNR
PiCesA2 848 ------------------------------AR----LVYALVPKLLIQGVLSA---LSNR
PvCesA1 841 ------------------------------LR----VLVALVPKLIVQGLLSA---LSTR
PiCesA1 840 ------------------------------LR----VLVALVPKLIVQGLLSA---MSTR
NsCesA 554 ------------------------------TD----FILHFLPTFIINRLTFV---VAIW
TvCesA 526 ------------------------------ID----FIVHFLPTFIINRLTFI---VAIW
JfCesA 415 ---------------------------LQDTW---------MAYIIVAQLLFTQVAGIFL
TlCesA 357 KPLVA---------------------YANDTQL-RWLIRACFANTIVNRLCEF---VL--
ThCesA 509 KPLVA---------------------YANDTQL-RWLIRACFANTIVNRLCEF---VL--
GpCesA 382 ---------------------------AGETKILMMFLG---PWLLMNRVLFY---SLYW
AmCesA 374 ---------------------------AGNTQTLVFLLV---PYLFCTRLIIY---VMYW
AtCslD6 812 ------KWSGISLEEWWRNEQFWLIGGTSAHLVA---------------VLQGILKV---
VvCslD3 963 ------KWSGITLEEWWRNEQFWLIGGTSAHLAA---------------VIQGLLKV---
CrCslD1 ------------------------------------------------------------
PpCslD8 1000 ------KWSGISLEEWWRNEQFWVIGGTSAHLAA---------------VFQGILKV---
AtCesA8 815 ------RWSGVSIEDLWRNEQFWVIGGVSAHLFA---------------VFQGFLKM---
VvCesA8 825 ------RWSGVSIEDLWRNEQFWVIGGVSAHLFA---------------VFQGFLKM---
VvCesA4 895 ----------------------------TAHLFA---------------VFQGLLKV---
VvCesA5 867 ------RWSGVGIDEWWRNEQFWVIGGVSAHLFA---------------VFQGLLKV---
AtCesA2 915 ------QWGGVGIDDWWRNEQFWVIGGASSHLFA---------------LFQGLLKV---
AtCesA6 916 ------QWGKVGIDDWWRNEQFWVIGGVSAHLFA---------------LFQGLLKV---
StCesA1 835 ------QWGGVSIDDWWRNEQFWVIGGASSHLFA---------------LFQGLLKV---
HaCesA2 927 ------QWGGVGIHDWWRNEQFWVIGGASSHLFA---------------LFQGLLKV---
VvCesA6 901 ------QWGRVAIDDWWRNEQFWVIGGASSHLFA---------------LFQGLLKV---
VvCesA9 581 ------QWGRVAIDDWWRNEQFWVIGGASSHLFA---------------LFQGLLKV---
PbCesA 928 ------RWSGVGIDEWWRNEQFWVIGGVSAHLFA---------------VFQGLLKV---
VvCesA7 908 ------RWSGVSIEEWWRNEQFWVIGGVSAHLFA---------------VVQGLLKV---
StCesA7 873 ------RWSGVSIEEWWRNEQFWVIGGVSAHLFA---------------VVQGLLKI---
VvCesA1 909 ------RWSGVSIEDWWRNEQFWVIGGTSAHLFA---------------VFQGLLKV---
ZmCesA2 904 ------RWSGVGIEDWWRNEQFWVIGGTSAHLFA---------------VFQGLLKV---
VvCesA3 939 ------RWSGVGIDEWWRNEQFWVIGGVSAHLFA---------------VFQGLLKV---
OsCesA2 904 ------RWSGVGIDEWWRNEQFWVIGGISAHLFA---------------VFQGLLKV---
AtCslB5 588 ------MSLGFSVQSWYVVQSLWRIIATSSWLFS---------------IQDIILKL---
StCslH1 589 ------IRANEPIIAWMNNQRMWRVNAMSAWLFG---------------ILSATTKL---
VvCslH1 591 ------WGAGYSIRACWNNLRMWRITAVTSWLFG---------------FLSVILKL---
VvCslB3 ------------------------------------------------------------
VvCslG2 502 ------ILAGGSIQTWSNEQRIWMIKSVTSHLYG---------------SLDAIMKR---
AtCslG3 596 ------VLEGGTYGGWWNDQRMWSIRGFSSHLFG---------------FIEFTLKT---
AtCslE1 573 ------LWCGGTFRGWWNEQRMWLYRRTSSFLFG---------------FMDTIKKL---
VvCslE6 608 ---------------------------------------------------ETILKL---
AtCslA3 291 ------TPRSLHLLVFW--------------------ILFENVMS-MHRTKA-TFIGLLE
AtCslA7 277 ------RLRSIHLLAFW--------------------VLFENAMS-LLRAKA-LVMGLFE
PbCCesA 413 -------LWSAQDYPWTVNQI--KVAQSYSHLFAIKDKLFGSVMT-WVPTGG-AVG----
BaCesA 407 ------AVSDRQRSMSW-------------------SHIYDTSMA-PHMAIS-ALSEFIF
AtmCesA 453 ------YLYGSFRWPWI-------------------SELYEYVQT-VHLLPA-VVSVIFN
RlCesA 464 ------YLYGSFRWPWI-------------------SELYEYVQT-VHLLPA-VVSVMLN
KxCesA3 455 ------KINKGWRYSFW-------------------SEVYETTMA-LFLVRV-TIVTLLS
EcCesA 579 ------KIQGKYRHSFW-------------------SEIYETVLA-WYIAPP-TLVALIN
PsCesA 851 ------RIQGRFRHSFW-------------------NEVYEAVLA-WYIMGP-VLMALVN
PfCesA 465 ------RIQGRFRHSFW-------------------NEVYETVLA-WYILPP-VLVALVN
PvCesA3 979 AVDNNDVWRA----------------------------QQ-TWFSFSFITMMA-------
PiCesA3 979 AVDNNDVWRA----------------------------QQ-TWFSFSFITMMA-------
PvCesA4 863 TVDNDDVLRS----------------------------QQ-TWFSYAFVHVMA-------
PiCesA4 863 TVDNDDVLRS----------------------------QQ-TWFSYAFVHVMA-------
PvCesA2 871 TVENSDVIRS----------------------------QE-VWFAYAFTNCTA-------
PiCesA2 871 TVDNSDVIRS----------------------------QE-VWFAYAFTNCTA-------
PvCesA1 864 GVQNSDVVRS----------------------------QE-TRFVYAFTNFTA-------
PiCesA1 863 GVENSDVVRS----------------------------QE-TRFVYAFTNFTA-------
NsCesA 577 GIPASEIWRS---------------------------EQYAIALF-PIFIQA-VWSVF--
TvCesA 549 GIPASEIWRS---------------------------EQYAIALF-PVFIQA-VWSVF--
JfCesA 439 SIPSGGDQFTEHLLSFW-------------------SSSQFAWMF-QYYHAKATIVSF--
TlCesA 390 FIPAGYHTGQ------R-------------------GSRYQLWMS-PYIALC-IIRSFVL
ThCesA 542 FIPAGYHTGQ------R-------------------GSRYQLWMS-PYIALC-IIRAFAL
GpCesA 409 GLPGGFQARN------R-------------------DPQLFIWMS-PYLFVA-LLKYM-F
AmCesA 401 NVPDAMTARN------R-------------------DFQLFLWMA-PYLCVA-LGKFL-M
AtCslD6 848 ---IAGVEISFTLTSKSSTGG----------DDEDDEFADL--YLFK-WTA---------
VvCslD3 999 ---VAGIEISFTLTSKS--AG----------DDADEDFADL--HLIK-WTS---------
CrCslD1 ------------------------------------------------------------
PpCslD8 1036 ---MAGVEISFTLTSKS--AG----------DDEDDIYADL--YIVK-WTS---------
AtCesA8 851 ---LAGLDTNFTVTSKT--------------AD-DLEFGEL--YIVK-WTT---------
VvCesA8 861 ---VAGLDTNFTVTAKA--------------AD-DGEFGEL--YMIK-WTT---------
VvCesA4 909 ---LAGVDTNFTVTSKA--------------AD-DAEFGDL--YLFK-WTT---------
VvCesA5 903 ---LAGVDTNFTVTSKA--------------GD-DVEFSEL--YAFK-WTT---------
AtCesA2 951 ---LAGVNTNFTVTSKA--------------AD-DGAFSEL--YIFK-WTT---------
AtCesA6 952 ---LAGVDTNFTVTSKA--------------AD-DGEFSDL--YLFK-WTS---------
StCesA1 871 ---LAGVNTSFTVTSKA--------------AD-DGEFSEL--YLFK-WTS---------
HaCesA2 963 ---LAGVNTNFTVTSKA--------------AD-DGEFSEL--YLFK-WTS---------
VvCesA6 937 ---LAGVNTNFTVTSKG--------------GD-DGEFSEL--YLFK-WTS---------
VvCesA9 617 ---LAGVNTNFTVTSKG--------------GD-DGEFSEL--YLFK-WTS---------
PbCesA 964 ---FAGIDTNFTVTSKS--------------SE-DEDFGEL--YAFK-WTS---------
VvCesA7 944 ---LAGIDTNFTVTSKA--------------VD-DEEFGEL--YTFK-WTT---------
StCesA7 909 ---LAGIDTNFTVTSKA--------------TD-DEDFGEL--YAFK-WTT---------
VvCesA1 945 ---LAGIDTNFTVTSKA--------------SDDDGDFAEL--YVFK-WTS---------
ZmCesA2 940 ---LAGIDTNFTVTSKA--------------SDEDGDFAEL--YVFK-WTS---------
VvCesA3 975 ---LAGIDTNFTVTSKA--------------SDEDGDFAEL--YMFK-WTT---------
OsCesA2 940 ---LAGIDTSFTVTSKA--------------SDEEGDFAEL--YMFK-WTT---------
AtCslB5 624 ---LGISQIGFVIAKKTIPETKSVYESKPSQGEDDVPKLNLGKFEFD-SSG---------
StCslH1 625 ---LGFSETAFEITKKDQNDT----------------NSDIGRFTFD-DSP---------
VvCslH1 627 ---LGLSETVFEVTKKDQSTT-------PG----EGSDKDSGRFTFD-GSL---------
VvCslB3 ------------------------------------------------------------
VvCslG2 538 ---ISMRKASFLPTNKVVD-S------------DHVKLYQMGKFDFRISTT---------
AtCslG3 632 ---LNLSTHGFNVTSKANDDE------------EQSKRYEKEIFEFGPSSS---------
AtCslE1 609 ---LGVSESAFVITAKVAE-E------------EAAERYKEEVMEFGVESP---------
VvCslE6 614 ---LGFAKSSFAVTSKVAD-E------------EESKRFEKEVMEFGAPSP---------
AtCslA3 323 ----AGRVNEWVVTEKLGDTL------------KSKLIGKATTKLY---TR---------
AtCslA7 309 ----TGRVQEWVVTEKLGDTL------------KTKLIPQVPN------VR---------
PbCCesA 458 -------------------VA------------TSRFN--QARILCGSWTT---------
BaCesA 440 ----KKR-FDFKVTPKGVNTD------------RRKFKYTTALPCA--------------
AtmCesA 486 ----PGK-PTFKVTAKDESIA------------EARLS-EISRPFF--------------
RlCesA 497 ----PRK-PTFKVTAKDESIA------------VSRLS-EISRPFF--------------
KxCesA3 488 ----PSR-GKFNVTDKGGLLE------------KGYFDLGAVYPNI--------------
EcCesA 612 ----PHK-GKFNVTAKGGLVE------------EEYVDWVISRPYI--------------
PsCesA 884 ----PKF-GGFNVTDKGGVVE------------EKFFDWTLARPYI--------------
PfCesA 498 ----PKA-GGFNVTDKGGIID------------KQFFDWKLARPYL--------------
PvCesA3 1003 ----IVEAIQARVTGKDKSWA------------NTGA-----------------GQKTSW
PiCesA3 1003 ----IVEAIQARVTGKDKSWA------------NTGA-----------------GQKTSW
PvCesA4 887 ----VFETIYWKITGKEATWA------------NTGA-----------------LGGNSI
PiCesA4 887 ----VFETIYWKITGKEAAWA------------NTGA-----------------LGGNSI
PvCesA2 895 ----VLEAFWWKITGREPKWF------------NTGG-----------------AKRGSI
PiCesA2 895 ----VLEAFWWKITGKEPKWF------------NTGG-----------------ASRGST
PvCesA1 888 ----MLGAIGWKLTGRTSQWR------------SKRD-----------------ASRGSL
PiCesA1 887 ----MLGAIVWKFTGRKSRWL------------NKRD-----------------ATRGSL
NsCesA 606 ----TGQKMNFQVTPKER-----------------------------------QSGIYLS
TvCesA 578 ----TGQKINFQVTPKQR-----------------------------------QSGIYLQ
JfCesA 477 ----LGRDLSFKVSDKGGPQR-------------------------------SHVSASLR
TlCesA 423 PKWLGGQTQAFKPTGSLSSAL------------NERDPKLKKNMFRRLWAILFNY-----
ThCesA 575 PKWLGGEAQAFKPTGSLSSAL------------NERDPKLKKNMFRRLWTILINY-----
GpCesA 441 P---FL-ETTFKVTNTA------------------------KNMEQTN-ALFT--LKGLR
AmCesA 433 S---GIKATSFKVTNAA------------------------PKKGKKRWCEFGELGSDLA
AtCslD6 883 LMIPPLTIIILNIVAILFAVCRTV-------FSANP-------QWSNLLGGTFFASWVLL
VvCslD3 1032 LMIPPVTIIITNLIGIAVGVVRTI-------YSELP-------QWSRLLGGVFFSFWVLV
CrCslD1 ------------------------------------------------------------
PpCslD8 1069 LFIPPITIGITNIVAIAVGVSRTI-------YSTNP-------EWSKLLGGVFFSLWVLM
AtCesA8 881 LLIPPTSLLIINLVGVVAGFSDAL-------NKGYE-------AWGPLFGKVFFAFWVIL
VvCesA8 891 LLIPPTTLLIINLVGVVAGFSDAL-------NSGYE-------AWGPLFGKVFFAFWVIL
VvCesA4 939 LLIPPTTLIILNMVGVVAGVSDAI-------NNGYG-------SWGPLFGKLFFAFWVIV
VvCesA5 933 LLIPPTTLLIINLIGVVAGISNAI-------NNGYE-------SWGPLFGKLFFAFWVIV
AtCesA2 981 LLIPPTTLLIINIIGVIVGVSDAI-------SNGYD-------SWGPLFGRLFFALWVIV
AtCesA6 982 LLIPPMTLLIINVIGVIVGVSDAI-------SNGYD-------SWGPLFGRLFFALWVII
StCesA1 901 LLIPPMTLLILNIIGVVVGVSDAI-------NNGYD-------SWGPLFGRLFFALWVIV
HaCesA2 993 LLIPPMTLLIINIIGVVVGISDAI-------NNGYE-------TWGPLFGKLFFALWVIV
VvCesA6 967 LLIPPLTLLILNIIGVMVGISDAI-------NNGYE-------EWGPLFGKLFFALWVIV
VvCesA9 647 LLIPPLTLLILNIIGVMVGISDAI-------NNGYE-------EWGPLFGKLFFALWVIV
PbCesA 994 LLIPPTTLLIINLVGVVAGISDAI-------NNGYQ-------TWGPLFGKIFFAFWVIV
VvCesA7 974 LLIPPTTLLIINLVGVVAGISDAI-------NNGYQ-------SWGPLFGKLFFAFWVIV
StCesA7 939 LLIPPTTILIINLVGVVAGISDAI-------NNGYN-------SWGPLFGKLFFAFWVIV
VvCesA1 976 LLIPPTTVLVVNLVGIVAGVSYAI-------NSGYQ-------SWGPLFGKLFFAIWVIV
ZmCesA2 971 LLIPPTTVLVINLVGMVAGISYAI-------NSGYQ-------SWGPLFGKLFFSIWVIL
VvCesA3 1006 LLIPPTTLLIINLVGVVAGISYAI-------NSGYQ-------SWGPLFGKLFFAFWVIV
OsCesA2 971 LLIPPTTILIINLVGVVAGISYAI-------NSGYQ-------SWGPLFGKLFFAFWVIV
AtCslB5 671 LFIPGTFIMLVNLAALAGYLVRLQ-------RSSCSH-----GGGGSGLAEACGCILVVM
StCslH1 656 IFVPGTAVLLLNLSALFIGVLDFK-------KGN---------NIEWGLGEVICIMWIVF
VvCslH1 663 IFVPATTLLLVHLMALVTALLGLF-------DLV---------GIESRIGEIICSVWVVL
VvCslB3 ------------------------------------------------------------
VvCslG2 573 VLASMVTLVVLNMVAFMAGLARAI-------VFGN--------WEKMLIQVLLSLY-ILI
AtCslG3 668 MFLPLTTVAIVNLLAFVWGLYGLF-------AWGE----------GLVLELMLASF-AVV
AtCslE1 644 MFLVLGTLGMLNLFCFAAAVARLV-------SGDG-G-----DLKTMGMQFVITGV-LVV
VvCslE6 649 MFTILATLALLNLFAFIGGIKRMI-------MDVPAH-----VLDSLLLQLLLCGV-LVF
AtCslA3 355 ------FGQRLNWRELVVGLYIFFCGCYDFAYGGSYF--------------YVYLFLQSC
AtCslA7 338 ------FRERVHLLELLVGAYLLFCGIYDIVYGKNTL--------------YVYLLFQSV
PbCCesA 476 ------FVVGFTYLMSAFRVYQGY----NYINFLPGI--------------ILTTINLFT
BaCesA 469 ------ILLAMTLFALGKILYELW-------FLHTFDMNVTSYHLFW---ALYNMTGLIL
AtmCesA 514 ------VIFALLLVAMAFAAWRIY----SEPYK----ADVTLVVGGW---NLLNLIFAGC
RlCesA 525 ------VIFAVQIVALIITIYKIY----AEPYK----ADVTLVVGGW---NVINLIMAGC
KxCesA3 517 ------ILGLIMFGGLARGVYELS-----FGHLDQIAERAYLLNSAW---AMLSLIIILA
EcCesA 641 ------FLVLLNLVGVAVGIWR-------YFYGPPTEMLTVVVSMVW---VFYNLIVLGG
PsCesA 913 ------VLLTLNAVVFALGIYSLY----QLGWNNDAITLTIVINMAW---TIYNIIITSA
PfCesA 527 ------VLLAVNLIGLGFGI---H----QLIWGDASTAVTVAINLTW---TLYNLIITSA
PvCesA3 1030 TEIPNVLFFFTLLFSQ---LVALI----RFFEYENA----T--NPWNYVSAMFFGFFVMS
PiCesA3 1030 TEIPNVLFFFTLLFSQ---LVALV----RFFEYENA----T--NPWNYVSAMFFGFFVMS
PvCesA4 914 MELPNLLVFLSMVFGM---IWDTV----RYFAGYNNAATTH--GTPLLFASLFLGGFLAS
PiCesA4 914 MELPNLLVFLAMVFGM---MWDTV----RYFAGYNNAATTH--GTPLYFASLFLGGFLAS
PvCesA2 922 AELPNVIIFFGTVVGI---LWAVV----RFLAGYNSIQTSH--GASLLFASLMMGLFLAV
PiCesA2 922 AELPNVIIFFGTVVGV---LWSVV----RFLAGYNSIQTSH--GASLLFASLMMGLFIAV
PvCesA1 915 AELPNVLLFSGAAFGI---IWATV----RYMVSYYNRVFSH--GDSMLCAAIFMGFHIAY
PiCesA1 914 AELPNVLVFSGAVFGI---IWAMV----RYIVAYYNRVFSH--GDSMLLAAMLMGFYIAY
NsCesA 627 MIWPQLLIFFLTIIGI---FWSLY----SFAIGRLNHPWVNLLNSSW---AVYNLLLLSG
TvCesA 599 MIWPQLMFLLLTIIGI---VWSLY----NFVIGHLNHPWVNLLNSSW---AIYNLLLLSG
JfCesA 502 LTWFNLVVSVIMLAAL---AYRIV----LLAIKAKNTGLNPQD-VQYLLSAV-FILNIIC
TlCesA 466 MAAFHFAFVYLTLVAATLVSYRCF----FQESHRVKDILICLVTHAFWPP-LTFIFICSS
ThCesA 618 MALFHLAFVYLTLVAVCLVSYRCF----FQESNRVRDILICLVTHAFWPP-LTFIFICSS
GpCesA 470 TVWFHLLYVLGSIGIF---VWRVY----VTNFS------KCEDTFKLVMQAIFLAYNVQN
AmCesA 466 KCSFHIAYCVLGLAAL---IYRGA----HWNPT------SCVDNFQFASQVLYVLLNVQS
AtCslD6 929 HMYPFAKGLMGRG--G------KTP------------T------------VVYVWS----
VvCslD3 1078 HLYPFAKGLMGRR--G------RTP------------T------------IVFVWA----
CrCslD1 ------------------------------------------------------------
PpCslD8 1115 HLYPFFKGLMGKG--G------KTP------------T------------IIYVWA----
AtCesA8 927 HLYPFLKGLMGRQ--N------RTP------------T------------IVILWS----
VvCesA8 937 HLYPFLKGLMGRQ--N------RTP------------T------------IVVLWS----
VvCesA4 985 HLYPFLKGLMGRQ--N------RTP------------T------------IVVLWS----
VvCesA5 979 HLYPFLKGLLGRQ--N------RTP------------T------------IIIVWS----
AtCesA2 1027 HLYPFLKGMLGKQ--D------KMP------------T------------IIVVWS----
AtCesA6 1028 HLYPFLKGLLGKQ--D------RMP------------T------------IIVVWS----
StCesA1 947 HLYPFLKGCMGRQ--N------NVP------------T------------IIIVWS----
HaCesA2 1039 HLYPFLKGLIGKQ--H------RLP------------T------------IIVVWS----
VvCesA6 1013 HLYPFLKGLMGKQ--D------RLP------------T------------IIVVWS----
VvCesA9 693 HLYPFLKGLMGKQ--D------RLP------------T------------IIVVWS----
PbCesA 1040 HLYPFLKGLMGRQ--N------RTP------------T------------IVIVWS----
VvCesA7 1020 HLYPFLKGLMGRQ--N------RTP------------T------------IVVIWS----
StCesA7 985 HLYPFLKGLMGRQ--N------RTP------------T------------IVVIWS----
VvCesA1 1022 HLYPFLKGLLGRQ--N------RTP------------T------------IVIVWS----
ZmCesA2 1017 HLYPFLKGLMGRQ--N------RTP------------T------------IVIVWS----
VvCesA3 1052 HLYPFLKGLMGRQ--N------RTP------------T------------IVVVWS----
OsCesA2 1017 HLYPFLKGLMGRQ--N------RTP------------T------------IVVVWA----
AtCslB5 719 LFLPFLKGLF-EHGKY------SIP------------L------------STLSKA----
StCslH1 700 VFWAFLKGLF-AKGKY------GIP------------T------------SSILKA----
VvCslH1 707 CFSPFLKGLF-GKGKY------GIP------------K------------STICKS----
VvCslB3 ------------------------------------------------------------
VvCslG2 617 MSYPVIEGMILRKDKG------RIP------------Y------------SVTLLS----
AtCslG3 710 NCLPIYEAMVLRIDDG------KLP------------K------------RVCFVA----
AtCslE1 690 INWPLYKGMLLRQDKG------KMP------------M------------SVTVKS----
VvCslE6 696 INLPVYQGLFLQKDAS------RVP------------Y------------SVTYQS----
AtCslA3 395 AFFVAGVGYIGTFVPTV-------------------------------------------
AtCslA7 378 AFFVVGFGFVGKYVPASSYLA---------------------------------------
PbCCesA 512 AL---PFIINRK------------------------------------------------
BaCesA 513 AL---LVAFDRPRFRSSERFTVNKPAAFSTVSGDEH-ECELIDVSDTGARIRLPYQADLH
AtmCesA 557 AL---GVVSERGDKSASRRITVKRRCEVKLEGSDAWVPASIDNVSVHGLLINLFDSAT-T
RlCesA 568 AL---GVVSERGERALSRRVRVNRRCEFGVNG--KWYAASIEDVSVHGARLHIFNKQLDE
KxCesA3 563 AI---AVGRETQQKRNSHRIPATIPVEVANADGSIIVTGVTEDLSMGGAAVKMSWPAK--
EcCesA 685 AV---AVSVESKQVRRSHRVEMTMPAAIAREDGHLF-SCTVQDFSDGGLGIKINGQAQ--
PsCesA 960 AI---AVASEIRQVRTEPRVQARLPIRVTRADGVVF-DAVTQDFSQTGLGLVMPADSG--
PfCesA 571 AV---AVASEARQVRSEPRVSAKLPVSIICADGRVL-DGTTQDFSQNGFGLMLSDGHS--
PvCesA3 1077 QFYPMVKMSI-----------TEY---C----GWDHTA-----ATFTAN-VFGSLL----
PiCesA3 1077 QFYPMVKMSI-----------TEY---C----GWDHTA-----ATFTAN-VFGSLL----
PvCesA4 965 QLGPMVRMSL-----------QTY---F----GWSHKS-----LTDQGN-MVGNFW----
PiCesA4 965 QLGPMVRMSL-----------QTY---F----GWSHKS-----LTDQGN-IVGSFS----
PvCesA2 973 KLAPSVRMSI-----------QEY---F----GWSYES-----LMDQGN-VVGSIS----
PiCesA2 973 KLAPSVRMSI-----------QEY---F----GWSYES-----LMDQGN-VVGSIS----
PvCesA1 966 NLGPTVRMSI-----------QEY---F----GWSYQS-----LMDQGN-FMGSIS----
PiCesA1 965 NLGPSVRMSI-----------QEY---F----GWSYQS-----LMDQGN-FMGSIS----
NsCesA 677 IIRASVWQM-----P-------KYD-SK----KV--------------------------
TvCesA 649 IIRASVWQM-----P-------KDH-SN----KI--------------------------
JfCesA 553 VQSQVVFLAIKDN------ILR----------GCSHIF----------VLVWQNLS----
TlCesA 521 LWTPIGYAIDPPQMPDREELLVRDP-QT----KVAHPTPESKKIAFGGQAAWFEFE----
ThCesA 673 LWTPVAYAIDPPQMPDREDLLVRDP-ET----KVAHPTTQSKKIAFGGQAAWFEFE----
GpCesA 517 MIPPVLFLVNPISVDPD--LVRTYD-EY----GIPHVP-LSATVPPTGKFIFILEI----
AmCesA 513 IAAPIVYVLSPDKGPADRRERLDYS-KF----GVPIVD-PVKVAPKPGKWVWLLEV----
AtCslD6 953 GLIAI-CLSLLYITIKNSE----------------IDGGSFMLV----------------
VvCslD3 1102 GLIAI-TISLLWVA----------------------------------------------
CrCslD1 ------------------------------------------------------------
PpCslD8 1139 GLLSV-IISLLWVYISPQDGGA-A-----------VGGGGFTFP----------------
AtCesA8 951 ILLAS-VFSLVWVRINPFVSKT-DTT--------SLSLNCLLIDC---------------
VvCesA8 961 VLLAS-VFSLVWVKINPFVQKV-DNS--------TIAQSCISIDC---------------
VvCesA4 1009 ILLAS-IFSLVWVRIDPFLPKQ-TG---------PVLKQC-GVEC---------------
VvCesA5 1003 ILLAS-IFSLLWVRIDPFLAKS-DG---------PVLEEC-GLDCN--------------
AtCesA2 1051 ILLAS-ILTLLWVRVNPFVAK--GG---------PVLEIC-GLNCGN-------------
AtCesA6 1052 ILLAS-ILTLLWVRVNPFVAK--GG---------PILEIC-GLDCL--------------
StCesA1 971 ILLAS-ICSLLWVRLNPFTAK--GG---------LS------------------------
HaCesA2 1063 ILLAS-VLTLLWVRINPFVSK--GG---------IVLEIC-GLNCD--------------
VvCesA6 1037 ILLAS-IFSLLWVRVNPFVSK--GG---------IVLEVC-GLDCD--------------
VvCesA9 717 ILLAS-IFSLLWVRVNPFVSK--GG---------IVLEVC-GLDCD--------------
PbCesA 1064 ILLAS-IFSLLWVRIDPFLTKV-TG---------PDLQQC-GINC---------------
VvCesA7 1044 VLLAS-IFSLLWVRIDPFILKT-KG---------PDVKQC-GINC---------------
StCesA7 1009 ILLAS-IFSLLWVRIDPFVLKT-KG---------PDVKRC-GVNC---------------
VvCesA1 1046 ILLAS-IFSLLWVRIDPFTSSS-TK---------AASGQC-GINC---------------
ZmCesA2 1041 ILLAS-IFSLLWVKIDPFISPT-QKA--------AALGQC-GVNC---------------
VvCesA3 1076 ILLAS-IFSLLWVRIDPFTTRV-TG---------PDVEQC-GINC---------------
OsCesA2 1041 ILLAS-IFSLLWVRIDPFTTRV-TG---------PDTQKC-GINC---------------
AtCslB5 744 AFLTV-LFVFFCVGK---------------------------------------------
StCslH1 725 GALAL-LLVHLFKFKQ--------------------------------------------
VvCslH1 732 AALAF-LFLACTITVRK-------------------------------------------
VvCslB3 ------------------------------------------------------------
VvCslG2 643 IVFAM-VFLTLGSVVLLY------------------------------------------
AtCslG3 736 GILTF-VLIVSGYVFLK-------------------------------------------
AtCslE1 716 VVLAL-SAC-TCLAFL--------------------------------------------
VvCslE6 722 IVFTL-LAC-SIALY---------------------------------------------
AtCslA3 ------------------------------------------------------------
AtCslA7 ------------------------------------------------------------
PbCCesA ------------------------------------------------------------
BaCesA 569 MYYHVDGL------IIDAVGKVPARVMWTTKDEADIEIGLHF-KEMDKELYVKLIGFMFN
AtmCesA 613 IEKGATAIVKVKPHSEGVPETMPVNVVRTVKGEGFVSIGCTF-SPQRAVDHRLIADLIFA
RlCesA 623 MLVGALGEIRFRPYSGAELETLPLIVRNIEPSGDISNVGCQY-VPKSALDHRLIADLMFA
KxCesA3 618 LSGPT--PVYIRTVLDGEELILPARIIRAGN----GRGIFIW-TIDNLQQEFSVIRLVFG
EcCesA 739 ILEGQ--KVNLLLKRGQQEYVFPTQVARVMG----NEVGLKL-MPLTTQQHIDFVQCTFA
PsCesA 1014 IDSGD--SITVSLYRGTQTSHFPATVMFCRD----GYLGTRF-DDLSLRQQSELVRLTFG
PfCesA 625 ITQGE--RVQLVLSRNGQDSLFDARVVFSKG----AQIGAQF-EALSLRQQSELVRLTFS
PvCesA3 1109 VVYIV-VFVQLWQV-----YYEGNLLVAQGT-----DGGGNA-ETGD---------A---
PiCesA3 1109 VVYIV-VFVQLWQV-----YYEGNLQVAQGT-----DAGGSA-AAT--------------
PvCesA4 997 LAFVL-ILLCIWVY-----VETPNHSIFG-------------------------------
PiCesA4 997 LAFVL-IILCIWVY-----VETPNHSIFG-------------------------------
PvCesA2 1005 IAFGL-IFVTLWVW-----IEQPTSNPF--------------------------------
PiCesA2 1005 IAFGL-IFITLWVW-----IEQPTSNPF--------------------------------
PvCesA1 998 IAVGL-MFISLWVH-----VEKPASS----------------------------------
PiCesA1 997 IAVGL-MFIALWVH-----VEEPASG----------------------------------
NsCesA ------------------------------------------------------------
TvCesA ------------------------------------------------------------
JfCesA 583 LFGVG-AALCI--------LAKLVGFSDV-------------------------------
TlCesA 572 YTFST-LYTCLIFV-----ASFLF------------------------------------
ThCesA 724 YTFST-LYTCLIFV-----ASFLF------------------------------------
GpCesA 565 IPAAL-ILVCMMVL-----VAEAAGVTDKLI-----GSSCAAAL----------------
AmCesA 563 VPVVW-GAFYLILF-----IGFASGYSF----------GCDLYFAGS---------LPEI
AtCslD6 ------------------------------------------------------------
VvCslD3 ------------------------------------------------------------
CrCslD1 ------------------------------------------------------------
PpCslD8 ------------------------------------------------------------
AtCesA8 ------------------------------------------------------------
VvCesA8 ------------------------------------------------------------
VvCesA4 ------------------------------------------------------------
VvCesA5 ------------------------------------------------------------
AtCesA2 ------------------------------------------------------------
AtCesA6 ------------------------------------------------------------
StCesA1 ------------------------------------------------------------
HaCesA2 ------------------------------------------------------------
VvCesA6 ------------------------------------------------------------
VvCesA9 ------------------------------------------------------------
PbCesA ------------------------------------------------------------
VvCesA7 ------------------------------------------------------------
StCesA7 ------------------------------------------------------------
VvCesA1 ------------------------------------------------------------
ZmCesA2 ------------------------------------------------------------
VvCesA3 ------------------------------------------------------------
OsCesA2 ------------------------------------------------------------
AtCslB5 ------------------------------------------------------------
StCslH1 ------------------------------------------------------------
VvCslH1 ------------------------------------------------------------
VvCslB3 ------------------------------------------------------------
VvCslG2 ------------------------------------------------------------
AtCslG3 ------------------------------------------------------------
AtCslE1 ------------------------------------------------------------
VvCslE6 ------------------------------------------------------------
AtCslA3 ------------------------------------------------------------
AtCslA7 ------------------------------------------------------------
PbCCesA ------------------------------------------------------------
BaCesA 622 EENAKKAD--REKRADTLST---VLRFFMKTEKSPDAFKRKHVREAFQGLGTLLF-----
AtmCesA 672 NSEQWSEFQRVRRKNPGLIRG-TAIFLAIS---------------LFQTQRGLYYLVRTL
RlCesA 682 NSDQWTEFQASRRRNPGLIRG-TIWFLGLS---------------LYQTSRGLIYFFRSM
KxCesA3 671 RADAWVD-WGNYKADRPLLSLMDMVL-SVKGLFRSS--------------GDIVH---RS
EcCesA 792 RADTWALWQDSYPEDKPLESLLDILKLGFRGYRHLAEFAPSSVKGIFRVLTSLVSWVVSF
PsCesA 1067 RADTWASTWGRGKPDTPLSALREVSHIGVRGVVELLKATRK---DF-----------SRL
PfCesA 678 RADTWAASWGAGQPDTPLAALREVGSIGIGGLFTLGRATLH---EL-----------RLA
PvCesA3 1145 -------AT-------TVT-----------------------------------------
PiCesA3 ------------------------------------------------------------
PvCesA4 ------------------------------------------------------------
PiCesA4 ------------------------------------------------------------
PvCesA2 ------------------------------------------------------------
PiCesA2 ------------------------------------------------------------
PvCesA1 ------------------------------------------------------------
PiCesA1 ------------------------------------------------------------
NsCesA ------------------------------------------------------------
TvCesA ------------------------------------------------------------
JfCesA ------------------------------------------------------------
TlCesA ------------------------------------------------------------
ThCesA ------------------------------------------------------------
GpCesA ------------------------------------------------------------
AmCesA 598 SSDDWSKAT-------KLA-----------------------------------------
AtCslD6 ------------------------------------------------------------
VvCslD3 ------------------------------------------------------------
CrCslD1 ------------------------------------------------------------
PpCslD8 ------------------------------------------------------------
AtCesA8 ------------------------------------------------------------
VvCesA8 ------------------------------------------------------------
VvCesA4 ------------------------------------------------------------
VvCesA5 ------------------------------------------------------------
AtCesA2 ------------------------------------------------------------
AtCesA6 ------------------------------------------------------------
StCesA1 ------------------------------------------------------------
HaCesA2 ------------------------------------------------------------
VvCesA6 ------------------------------------------------------------
VvCesA9 ------------------------------------------------------------
PbCesA ------------------------------------------------------------
VvCesA7 ------------------------------------------------------------
StCesA7 ------------------------------------------------------------
VvCesA1 ------------------------------------------------------------
ZmCesA2 ------------------------------------------------------------
VvCesA3 ------------------------------------------------------------
OsCesA2 ------------------------------------------------------------
AtCslB5 ------------------------------------------------------------
StCslH1 ------------------------------------------------------------
VvCslH1 ------------------------------------------------------------
VvCslB3 ------------------------------------------------------------
VvCslG2 ------------------------------------------------------------
AtCslG3 ------------------------------------------------------------
AtCslE1 ------------------------------------------------------------
VvCslE6 ------------------------------------------------------------
AtCslA3 ------------------------------------------------------------
AtCslA7 ------------------------------------------------------------
PbCCesA ------------------------------------------------------------
BaCesA 672 -PDDAEKGAHEIMIKDISL--SGCQIESGVPLEMNEHVLVSINEKDLDQRLALVCWIKKR
AtmCesA 716 RPAP-RDAKPVG--A----------VK---------------------------------
RlCesA 726 RPER-EAQQKAA--K----------VNAG-------------------------------
KxCesA3 712 SPTKPSAGNALS--DDTNNPSRKERVLKGT--------------VKMVSLLALLTFASSA
EcCesA 852 IPRRPER-------SETAQPSDQALAQQ--------------------------------
PsCesA 1113 LPTR----------KKISPPPAN-------------------------------------
PfCesA 724 LSRTPTK--PLD--TLMDKP----------------------------------------
PvCesA3 ------------------------------------------------------------
PiCesA3 ------------------------------------------------------------
PvCesA4 ------------------------------------------------------------
PiCesA4 ------------------------------------------------------------
PvCesA2 ------------------------------------------------------------
PiCesA2 ------------------------------------------------------------
PvCesA1 ------------------------------------------------------------
PiCesA1 ------------------------------------------------------------
NsCesA ------------------------------------------------------------
TvCesA ------------------------------------------------------------
JfCesA ------------------------------------------------------------
TlCesA ------------------------------------------------------------
ThCesA ------------------------------------------------------------
GpCesA ------------------------------------------------------------
AmCesA ------------------------------------------------------------
AtCslD6 ------------------------------------------------------------
VvCslD3 ------------------------------------------------------------
CrCslD1 ------------------------------------------------------------
PpCslD8 ------------------------------------------------------------
AtCesA8 ------------------------------------------------------------
VvCesA8 ------------------------------------------------------------
VvCesA4 ------------------------------------------------------------
VvCesA5 ------------------------------------------------------------
AtCesA2 ------------------------------------------------------------
AtCesA6 ------------------------------------------------------------
StCesA1 ------------------------------------------------------------
HaCesA2 ------------------------------------------------------------
VvCesA6 ------------------------------------------------------------
VvCesA9 ------------------------------------------------------------
PbCesA ------------------------------------------------------------
VvCesA7 ------------------------------------------------------------
StCesA7 ------------------------------------------------------------
VvCesA1 ------------------------------------------------------------
ZmCesA2 ------------------------------------------------------------
VvCesA3 ------------------------------------------------------------
OsCesA2 ------------------------------------------------------------
AtCslB5 ------------------------------------------------------------
StCslH1 ------------------------------------------------------------
VvCslH1 ------------------------------------------------------------
VvCslB3 ------------------------------------------------------------
VvCslG2 ------------------------------------------------------------
AtCslG3 ------------------------------------------------------------
AtCslE1 ------------------------------------------------------------
VvCslE6 ------------------------------------------------------------
AtCslA3 ------------------------------------------------------------
AtCslA7 ------------------------------------------------------------
PbCCesA ------------------------------------------------------------
BaCesA 729 RKR----------------------YTAGVKFIDGYAA------------QSIGDPVA--
AtmCesA ------------------------------------------------------------
RlCesA ------------------------------------------------------------
KxCesA3 756 QAASAPRAVAAKAPAHQPEASDLPPLPALLPATSGAAQAGSGDAGADGPGSPTGQPLAAD
EcCesA ------------------------------------------------------------
PsCesA ------------------------------------------------------------
PfCesA ------------------------------------------------------------
PvCesA3 ------------------------------------------------------------
PiCesA3 ------------------------------------------------------------
PvCesA4 ------------------------------------------------------------
PiCesA4 ------------------------------------------------------------
PvCesA2 ------------------------------------------------------------
PiCesA2 ------------------------------------------------------------
PvCesA1 ------------------------------------------------------------
PiCesA1 ------------------------------------------------------------
NsCesA ------------------------------------------------------------
TvCesA ------------------------------------------------------------
JfCesA ------------------------------------------------------------
TlCesA ------------------------------------------------------------
ThCesA ------------------------------------------------------------
GpCesA ------------------------------------------------------------
AmCesA ------------------------------------------------------------
AtCslD6 ------------------------------------------------------------
VvCslD3 ------------------------------------------------------------
CrCslD1 ------------------------------------------------------------
PpCslD8 ------------------------------------------------------------
AtCesA8 ------------------------------------------------------------
VvCesA8 ------------------------------------------------------------
VvCesA4 ------------------------------------------------------------
VvCesA5 ------------------------------------------------------------
AtCesA2 ------------------------------------------------------------
AtCesA6 ------------------------------------------------------------
StCesA1 ------------------------------------------------------------
HaCesA2 ------------------------------------------------------------
VvCesA6 ------------------------------------------------------------
VvCesA9 ------------------------------------------------------------
PbCesA ------------------------------------------------------------
VvCesA7 ------------------------------------------------------------
StCesA7 ------------------------------------------------------------
VvCesA1 ------------------------------------------------------------
ZmCesA2 ------------------------------------------------------------
VvCesA3 ------------------------------------------------------------
OsCesA2 ------------------------------------------------------------
AtCslB5 ------------------------------------------------------------
StCslH1 ------------------------------------------------------------
VvCslH1 ------------------------------------------------------------
VvCslB3 ------------------------------------------------------------
VvCslG2 ------------------------------------------------------------
AtCslG3 ------------------------------------------------------------
AtCslE1 ------------------------------------------------------------
VvCslE6 ------------------------------------------------------------
AtCslA3 ------------------------------------------------------------
AtCslA7 ------------------------------------------------------------
PbCCesA ------------------------------------------------------------
BaCesA ------------------------------------------------------------
AtmCesA ------------------------------------------------------------
RlCesA ------------------------------------------------------------
KxCesA3 816 SADALVENAENTSDTATVHNYTLKDLGAAGSITMRGLAPLQGIEFGIPSDQLVTSARLVL
EcCesA ------------------------------------------------------------
PsCesA ------------------------------------------------------------
PfCesA ------------------------------------------------------------
PvCesA3 ------------------------------------------------------------
PiCesA3 ------------------------------------------------------------
PvCesA4 ------------------------------------------------------------
PiCesA4 ------------------------------------------------------------
PvCesA2 ------------------------------------------------------------
PiCesA2 ------------------------------------------------------------
PvCesA1 ------------------------------------------------------------
PiCesA1 ------------------------------------------------------------
NsCesA ------------------------------------------------------------
TvCesA ------------------------------------------------------------
JfCesA ------------------------------------------------------------
TlCesA ------------------------------------------------------------
ThCesA ------------------------------------------------------------
GpCesA ------------------------------------------------------------
AmCesA ------------------------------------------------------------
AtCslD6 ------------------------------------------------------------
VvCslD3 ------------------------------------------------------------
CrCslD1 ------------------------------------------------------------
PpCslD8 ------------------------------------------------------------
AtCesA8 ------------------------------------------------------------
VvCesA8 ------------------------------------------------------------
VvCesA4 ------------------------------------------------------------
VvCesA5 ------------------------------------------------------------
AtCesA2 ------------------------------------------------------------
AtCesA6 ------------------------------------------------------------
StCesA1 ------------------------------------------------------------
HaCesA2 ------------------------------------------------------------
VvCesA6 ------------------------------------------------------------
VvCesA9 ------------------------------------------------------------
PbCesA ------------------------------------------------------------
VvCesA7 ------------------------------------------------------------
StCesA7 ------------------------------------------------------------
VvCesA1 ------------------------------------------------------------
ZmCesA2 ------------------------------------------------------------
VvCesA3 ------------------------------------------------------------
OsCesA2 ------------------------------------------------------------
AtCslB5 ------------------------------------------------------------
StCslH1 ------------------------------------------------------------
VvCslH1 ------------------------------------------------------------
VvCslB3 ------------------------------------------------------------
VvCslG2 ------------------------------------------------------------
AtCslG3 ------------------------------------------------------------
AtCslE1 ------------------------------------------------------------
VvCslE6 ------------------------------------------------------------
AtCslA3 ------------------------------------------------------------
AtCslA7 ------------------------------------------------------------
PbCCesA ------------------------------------------------------------
BaCesA ------------------------------------------------------------
AtmCesA ------------------------------------------------------------
RlCesA ------------------------------------------------------------
KxCesA3 876 SGSMSPNLRPETNSVTMTLNEQYIGTLRPDPAHPTFGPMSFEINPIFFVSGNRLNFNFAS
EcCesA ------------------------------------------------------------
PsCesA ------------------------------------------------------------
PfCesA ------------------------------------------------------------
PvCesA3 ------------------------------------------------------------
PiCesA3 ------------------------------------------------------------
PvCesA4 ------------------------------------------------------------
PiCesA4 ------------------------------------------------------------
PvCesA2 ------------------------------------------------------------
PiCesA2 ------------------------------------------------------------
PvCesA1 ------------------------------------------------------------
PiCesA1 ------------------------------------------------------------
NsCesA ------------------------------------------------------------
TvCesA ------------------------------------------------------------
JfCesA ------------------------------------------------------------
TlCesA ------------------------------------------------------------
ThCesA ------------------------------------------------------------
GpCesA ------------------------------------------------------------
AmCesA ------------------------------------------------------------
AtCslD6 ------------------------------------------------------------
VvCslD3 ------------------------------------------------------------
CrCslD1 ------------------------------------------------------------
PpCslD8 ------------------------------------------------------------
AtCesA8 ------------------------------------------------------------
VvCesA8 ------------------------------------------------------------
VvCesA4 ------------------------------------------------------------
VvCesA5 ------------------------------------------------------------
AtCesA2 ------------------------------------------------------------
AtCesA6 ------------------------------------------------------------
StCesA1 ------------------------------------------------------------
HaCesA2 ------------------------------------------------------------
VvCesA6 ------------------------------------------------------------
VvCesA9 ------------------------------------------------------------
PbCesA ------------------------------------------------------------
VvCesA7 ------------------------------------------------------------
StCesA7 ------------------------------------------------------------
VvCesA1 ------------------------------------------------------------
ZmCesA2 ------------------------------------------------------------
VvCesA3 ------------------------------------------------------------
OsCesA2 ------------------------------------------------------------
AtCslB5 ------------------------------------------------------------
StCslH1 ------------------------------------------------------------
VvCslH1 ------------------------------------------------------------
VvCslB3 ------------------------------------------------------------
VvCslG2 ------------------------------------------------------------
AtCslG3 ------------------------------------------------------------
AtCslE1 ------------------------------------------------------------
VvCslE6 ------------------------------------------------------------
AtCslA3 ------------------------------------------------------------
AtCslA7 ------------------------------------------------------------
PbCCesA ------------------------------------------------------------
BaCesA ------------------------------------------------------------
AtmCesA ------------------------------------------------------------
RlCesA ------------------------------------------------------------
KxCesA3 936 GSKGCSDITNDTLWATISQNSQLQITTIALPPRRLLSRLPQPFYDKNVRQHVTVPMVLAQ
EcCesA ------------------------------------------------------------
PsCesA ------------------------------------------------------------
PfCesA ------------------------------------------------------------
PvCesA3 ------------------------------------------------------------
PiCesA3 ------------------------------------------------------------
PvCesA4 ------------------------------------------------------------
PiCesA4 ------------------------------------------------------------
PvCesA2 ------------------------------------------------------------
PiCesA2 ------------------------------------------------------------
PvCesA1 ------------------------------------------------------------
PiCesA1 ------------------------------------------------------------
NsCesA ------------------------------------------------------------
TvCesA ------------------------------------------------------------
JfCesA ------------------------------------------------------------
TlCesA ------------------------------------------------------------
ThCesA ------------------------------------------------------------
GpCesA ------------------------------------------------------------
AmCesA ------------------------------------------------------------
AtCslD6 ------------------------------------------------------------
VvCslD3 ------------------------------------------------------------
CrCslD1 ------------------------------------------------------------
PpCslD8 ------------------------------------------------------------
AtCesA8 ------------------------------------------------------------
VvCesA8 ------------------------------------------------------------
VvCesA4 ------------------------------------------------------------
VvCesA5 ------------------------------------------------------------
AtCesA2 ------------------------------------------------------------
AtCesA6 ------------------------------------------------------------
StCesA1 ------------------------------------------------------------
HaCesA2 ------------------------------------------------------------
VvCesA6 ------------------------------------------------------------
VvCesA9 ------------------------------------------------------------
PbCesA ------------------------------------------------------------
VvCesA7 ------------------------------------------------------------
StCesA7 ------------------------------------------------------------
VvCesA1 ------------------------------------------------------------
ZmCesA2 ------------------------------------------------------------
VvCesA3 ------------------------------------------------------------
OsCesA2 ------------------------------------------------------------
AtCslB5 ------------------------------------------------------------
StCslH1 ------------------------------------------------------------
VvCslH1 ------------------------------------------------------------
VvCslB3 ------------------------------------------------------------
VvCslG2 ------------------------------------------------------------
AtCslG3 ------------------------------------------------------------
AtCslE1 ------------------------------------------------------------
VvCslE6 ------------------------------------------------------------
AtCslA3 ------------------------------------------------------------
AtCslA7 ------------------------------------------------------------
PbCCesA ------------------------------------------------------------
BaCesA ------------------------------------------------------------
AtmCesA ------------------------------------------------------------
RlCesA ------------------------------------------------------------
KxCesA3 996 TYDPQILKSAGILASWFGKQTDFLGVTFPVSSTIPQSGNAILIGVADELPTSFGRPQVNG
EcCesA ------------------------------------------------------------
PsCesA ------------------------------------------------------------
PfCesA ------------------------------------------------------------
PvCesA3 ------------------------------------------------------------
PiCesA3 ------------------------------------------------------------
PvCesA4 ------------------------------------------------------------
PiCesA4 ------------------------------------------------------------
PvCesA2 ------------------------------------------------------------
PiCesA2 ------------------------------------------------------------
PvCesA1 ------------------------------------------------------------
PiCesA1 ------------------------------------------------------------
NsCesA ------------------------------------------------------------
TvCesA ------------------------------------------------------------
JfCesA ------------------------------------------------------------
TlCesA ------------------------------------------------------------
ThCesA ------------------------------------------------------------
GpCesA ------------------------------------------------------------
AmCesA ------------------------------------------------------------
AtCslD6 ------------------------------------------------------------
VvCslD3 ------------------------------------------------------------
CrCslD1 ------------------------------------------------------------
PpCslD8 ------------------------------------------------------------
AtCesA8 ------------------------------------------------------------
VvCesA8 ------------------------------------------------------------
VvCesA4 ------------------------------------------------------------
VvCesA5 ------------------------------------------------------------
AtCesA2 ------------------------------------------------------------
AtCesA6 ------------------------------------------------------------
StCesA1 ------------------------------------------------------------
HaCesA2 ------------------------------------------------------------
VvCesA6 ------------------------------------------------------------
VvCesA9 ------------------------------------------------------------
PbCesA ------------------------------------------------------------
VvCesA7 ------------------------------------------------------------
StCesA7 ------------------------------------------------------------
VvCesA1 ------------------------------------------------------------
ZmCesA2 ------------------------------------------------------------
VvCesA3 ------------------------------------------------------------
OsCesA2 ------------------------------------------------------------
AtCslB5 ------------------------------------------------------------
StCslH1 ------------------------------------------------------------
VvCslH1 ------------------------------------------------------------
VvCslB3 ------------------------------------------------------------
VvCslG2 ------------------------------------------------------------
AtCslG3 ------------------------------------------------------------
AtCslE1 ------------------------------------------------------------
VvCslE6 ------------------------------------------------------------
AtCslA3 ------------------------------------------------------------
AtCslA7 ------------------------------------------------------------
PbCCesA ------------------------------------------------------------
BaCesA ------------------------------------------------------------
AtmCesA ------------------------------------------------------------
RlCesA ------------------------------------------------------------
KxCesA3 1056 PAVLELPNPSDANATILVVTGRDRDEVITASKGIAFASAPLPTDSHMDVAPVDIAPRKPN
EcCesA ------------------------------------------------------------
PsCesA ------------------------------------------------------------
PfCesA ------------------------------------------------------------
PvCesA3 ------------------------------------------------------------
PiCesA3 ------------------------------------------------------------
PvCesA4 ------------------------------------------------------------
PiCesA4 ------------------------------------------------------------
PvCesA2 ------------------------------------------------------------
PiCesA2 ------------------------------------------------------------
PvCesA1 ------------------------------------------------------------
PiCesA1 ------------------------------------------------------------
NsCesA ------------------------------------------------------------
TvCesA ------------------------------------------------------------
JfCesA ------------------------------------------------------------
TlCesA ------------------------------------------------------------
ThCesA ------------------------------------------------------------
GpCesA ------------------------------------------------------------
AmCesA ------------------------------------------------------------
AtCslD6 ------------------------------------------------------------
VvCslD3 ------------------------------------------------------------
CrCslD1 ------------------------------------------------------------
PpCslD8 ------------------------------------------------------------
AtCesA8 ------------------------------------------------------------
VvCesA8 ------------------------------------------------------------
VvCesA4 ------------------------------------------------------------
VvCesA5 ------------------------------------------------------------
AtCesA2 ------------------------------------------------------------
AtCesA6 ------------------------------------------------------------
StCesA1 ------------------------------------------------------------
HaCesA2 ------------------------------------------------------------
VvCesA6 ------------------------------------------------------------
VvCesA9 ------------------------------------------------------------
PbCesA ------------------------------------------------------------
VvCesA7 ------------------------------------------------------------
StCesA7 ------------------------------------------------------------
VvCesA1 ------------------------------------------------------------
ZmCesA2 ------------------------------------------------------------
VvCesA3 ------------------------------------------------------------
OsCesA2 ------------------------------------------------------------
AtCslB5 ------------------------------------------------------------
StCslH1 ------------------------------------------------------------
VvCslH1 ------------------------------------------------------------
VvCslB3 ------------------------------------------------------------
VvCslG2 ------------------------------------------------------------
AtCslG3 ------------------------------------------------------------
AtCslE1 ------------------------------------------------------------
VvCslE6 ------------------------------------------------------------
AtCslA3 ------------------------------------------------------------
AtCslA7 ------------------------------------------------------------
PbCCesA ------------------------------------------------------------
BaCesA ------------------------------------------------------------
AtmCesA ------------------------------------------------------------
RlCesA ------------------------------------------------------------
KxCesA3 1116 DAPSFIAMDHPVRFGDLVTASKLQGTGFTSGVLSVPFRIPPDLYTWRNRPYKMQVRFRSP
EcCesA ------------------------------------------------------------
PsCesA ------------------------------------------------------------
PfCesA ------------------------------------------------------------
PvCesA3 ------------------------------------------------------------
PiCesA3 ------------------------------------------------------------
PvCesA4 ------------------------------------------------------------
PiCesA4 ------------------------------------------------------------
PvCesA2 ------------------------------------------------------------
PiCesA2 ------------------------------------------------------------
PvCesA1 ------------------------------------------------------------
PiCesA1 ------------------------------------------------------------
NsCesA ------------------------------------------------------------
TvCesA ------------------------------------------------------------
JfCesA ------------------------------------------------------------
TlCesA ------------------------------------------------------------
ThCesA ------------------------------------------------------------
GpCesA ------------------------------------------------------------
AmCesA ------------------------------------------------------------
AtCslD6 ------------------------------------------------------------
VvCslD3 ------------------------------------------------------------
CrCslD1 ------------------------------------------------------------
PpCslD8 ------------------------------------------------------------
AtCesA8 ------------------------------------------------------------
VvCesA8 ------------------------------------------------------------
VvCesA4 ------------------------------------------------------------
VvCesA5 ------------------------------------------------------------
AtCesA2 ------------------------------------------------------------
AtCesA6 ------------------------------------------------------------
StCesA1 ------------------------------------------------------------
HaCesA2 ------------------------------------------------------------
VvCesA6 ------------------------------------------------------------
VvCesA9 ------------------------------------------------------------
PbCesA ------------------------------------------------------------
VvCesA7 ------------------------------------------------------------
StCesA7 ------------------------------------------------------------
VvCesA1 ------------------------------------------------------------
ZmCesA2 ------------------------------------------------------------
VvCesA3 ------------------------------------------------------------
OsCesA2 ------------------------------------------------------------
AtCslB5 ------------------------------------------------------------
StCslH1 ------------------------------------------------------------
VvCslH1 ------------------------------------------------------------
VvCslB3 ------------------------------------------------------------
VvCslG2 ------------------------------------------------------------
AtCslG3 ------------------------------------------------------------
AtCslE1 ------------------------------------------------------------
VvCslE6 ------------------------------------------------------------
AtCslA3 ------------------------------------------------------------
AtCslA7 ------------------------------------------------------------
PbCCesA ------------------------------------------------------------
BaCesA ------------------------------------------------------------
AtmCesA ------------------------------------------------------------
RlCesA ------------------------------------------------------------
KxCesA3 1176 AGEAKDVEKSRLDVGINEVYLHSYPLRETHGLIGAVLQGVGLARPASGMQVHDLDVPPWT
EcCesA ------------------------------------------------------------
PsCesA ------------------------------------------------------------
PfCesA ------------------------------------------------------------
PvCesA3 ------------------------------------------------------------
PiCesA3 ------------------------------------------------------------
PvCesA4 ------------------------------------------------------------
PiCesA4 ------------------------------------------------------------
PvCesA2 ------------------------------------------------------------
PiCesA2 ------------------------------------------------------------
PvCesA1 ------------------------------------------------------------
PiCesA1 ------------------------------------------------------------
NsCesA ------------------------------------------------------------
TvCesA ------------------------------------------------------------
JfCesA ------------------------------------------------------------
TlCesA ------------------------------------------------------------
ThCesA ------------------------------------------------------------
GpCesA ------------------------------------------------------------
AmCesA ------------------------------------------------------------
AtCslD6 ------------------------------------------------------------
VvCslD3 ------------------------------------------------------------
CrCslD1 ------------------------------------------------------------
PpCslD8 ------------------------------------------------------------
AtCesA8 ------------------------------------------------------------
VvCesA8 ------------------------------------------------------------
VvCesA4 ------------------------------------------------------------
VvCesA5 ------------------------------------------------------------
AtCesA2 ------------------------------------------------------------
AtCesA6 ------------------------------------------------------------
StCesA1 ------------------------------------------------------------
HaCesA2 ------------------------------------------------------------
VvCesA6 ------------------------------------------------------------
VvCesA9 ------------------------------------------------------------
PbCesA ------------------------------------------------------------
VvCesA7 ------------------------------------------------------------
StCesA7 ------------------------------------------------------------
VvCesA1 ------------------------------------------------------------
ZmCesA2 ------------------------------------------------------------
VvCesA3 ------------------------------------------------------------
OsCesA2 ------------------------------------------------------------
AtCslB5 ------------------------------------------------------------
StCslH1 ------------------------------------------------------------
VvCslH1 ------------------------------------------------------------
VvCslB3 ------------------------------------------------------------
VvCslG2 ------------------------------------------------------------
AtCslG3 ------------------------------------------------------------
AtCslE1 ------------------------------------------------------------
VvCslE6 ------------------------------------------------------------
AtCslA3 ------------------------------------------------------------
AtCslA7 ------------------------------------------------------------
PbCCesA ------------------------------------------------------------
BaCesA ------------------------------------------------------------
AtmCesA ------------------------------------------------------------
RlCesA ------------------------------------------------------------
KxCesA3 1236 VFGQDQLNFYFDAMPLARGICQSGAANNAFHLGLDPDSTIDFSRAHHIAQMPNLAYMATV
EcCesA ------------------------------------------------------------
PsCesA ------------------------------------------------------------
PfCesA ------------------------------------------------------------
PvCesA3 ------------------------------------------------------------
PiCesA3 ------------------------------------------------------------
PvCesA4 ------------------------------------------------------------
PiCesA4 ------------------------------------------------------------
PvCesA2 ------------------------------------------------------------
PiCesA2 ------------------------------------------------------------
PvCesA1 ------------------------------------------------------------
PiCesA1 ------------------------------------------------------------
NsCesA ------------------------------------------------------------
TvCesA ------------------------------------------------------------
JfCesA ------------------------------------------------------------
TlCesA ------------------------------------------------------------
ThCesA ------------------------------------------------------------
GpCesA ------------------------------------------------------------
AmCesA ------------------------------------------------------------
AtCslD6 ------------------------------------------------------------
VvCslD3 ------------------------------------------------------------
CrCslD1 ------------------------------------------------------------
PpCslD8 ------------------------------------------------------------
AtCesA8 ------------------------------------------------------------
VvCesA8 ------------------------------------------------------------
VvCesA4 ------------------------------------------------------------
VvCesA5 ------------------------------------------------------------
AtCesA2 ------------------------------------------------------------
AtCesA6 ------------------------------------------------------------
StCesA1 ------------------------------------------------------------
HaCesA2 ------------------------------------------------------------
VvCesA6 ------------------------------------------------------------
VvCesA9 ------------------------------------------------------------
PbCesA ------------------------------------------------------------
VvCesA7 ------------------------------------------------------------
StCesA7 ------------------------------------------------------------
VvCesA1 ------------------------------------------------------------
ZmCesA2 ------------------------------------------------------------
VvCesA3 ------------------------------------------------------------
OsCesA2 ------------------------------------------------------------
AtCslB5 ------------------------------------------------------------
StCslH1 ------------------------------------------------------------
VvCslH1 ------------------------------------------------------------
VvCslB3 ------------------------------------------------------------
VvCslG2 ------------------------------------------------------------
AtCslG3 ------------------------------------------------------------
AtCslE1 ------------------------------------------------------------
VvCslE6 ------------------------------------------------------------
AtCslA3 ------------------------------------------------------------
AtCslA7 ------------------------------------------------------------
PbCCesA ------------------------------------------------------------
BaCesA ------------------------------------------------------------
AtmCesA ------------------------------------------------------------
RlCesA ------------------------------------------------------------
KxCesA3 1296 GFPFTTYADLSQTAVVLPEHPNAATVGAYLDLMGFMGAATWYPVAGVDIVSADHVSDVAD
EcCesA ------------------------------------------------------------
PsCesA ------------------------------------------------------------
PfCesA ------------------------------------------------------------
PvCesA3 ------------------------------------------------------------
PiCesA3 ------------------------------------------------------------
PvCesA4 ------------------------------------------------------------
PiCesA4 ------------------------------------------------------------
PvCesA2 ------------------------------------------------------------
PiCesA2 ------------------------------------------------------------
PvCesA1 ------------------------------------------------------------
PiCesA1 ------------------------------------------------------------
NsCesA ------------------------------------------------------------
TvCesA ------------------------------------------------------------
JfCesA ------------------------------------------------------------
TlCesA ------------------------------------------------------------
ThCesA ------------------------------------------------------------
GpCesA ------------------------------------------------------------
AmCesA ------------------------------------------------------------
AtCslD6 ------------------------------------------------------------
VvCslD3 ------------------------------------------------------------
CrCslD1 ------------------------------------------------------------
PpCslD8 ------------------------------------------------------------
AtCesA8 ------------------------------------------------------------
VvCesA8 ------------------------------------------------------------
VvCesA4 ------------------------------------------------------------
VvCesA5 ------------------------------------------------------------
AtCesA2 ------------------------------------------------------------
AtCesA6 ------------------------------------------------------------
StCesA1 ------------------------------------------------------------
HaCesA2 ------------------------------------------------------------
VvCesA6 ------------------------------------------------------------
VvCesA9 ------------------------------------------------------------
PbCesA ------------------------------------------------------------
VvCesA7 ------------------------------------------------------------
StCesA7 ------------------------------------------------------------
VvCesA1 ------------------------------------------------------------
ZmCesA2 ------------------------------------------------------------
VvCesA3 ------------------------------------------------------------
OsCesA2 ------------------------------------------------------------
AtCslB5 ------------------------------------------------------------
StCslH1 ------------------------------------------------------------
VvCslH1 ------------------------------------------------------------
VvCslB3 ------------------------------------------------------------
VvCslG2 ------------------------------------------------------------
AtCslG3 ------------------------------------------------------------
AtCslE1 ------------------------------------------------------------
VvCslE6 ------------------------------------------------------------
AtCslA3 ------------------------------------------------------------
AtCslA7 ------------------------------------------------------------
PbCCesA ------------------------------------------------------------
BaCesA ------------------------------------------------------------
AtmCesA ------------------------------------------------------------
RlCesA ------------------------------------------------------------
KxCesA3 1356 RNLLVISTLATSGEIAPLLSRSSYEVADGHLRTVSHASALDNAIKAVDDPLTAFRDRDSK
EcCesA ------------------------------------------------------------
PsCesA ------------------------------------------------------------
PfCesA ------------------------------------------------------------
PvCesA3 ------------------------------------------------------------
PiCesA3 ------------------------------------------------------------
PvCesA4 ------------------------------------------------------------
PiCesA4 ------------------------------------------------------------
PvCesA2 ------------------------------------------------------------
PiCesA2 ------------------------------------------------------------
PvCesA1 ------------------------------------------------------------
PiCesA1 ------------------------------------------------------------
NsCesA ------------------------------------------------------------
TvCesA ------------------------------------------------------------
JfCesA ------------------------------------------------------------
TlCesA ------------------------------------------------------------
ThCesA ------------------------------------------------------------
GpCesA ------------------------------------------------------------
AmCesA ------------------------------------------------------------
AtCslD6 ------------------------------------------------------------
VvCslD3 ------------------------------------------------------------
CrCslD1 ------------------------------------------------------------
PpCslD8 ------------------------------------------------------------
AtCesA8 ------------------------------------------------------------
VvCesA8 ------------------------------------------------------------
VvCesA4 ------------------------------------------------------------
VvCesA5 ------------------------------------------------------------
AtCesA2 ------------------------------------------------------------
AtCesA6 ------------------------------------------------------------
StCesA1 ------------------------------------------------------------
HaCesA2 ------------------------------------------------------------
VvCesA6 ------------------------------------------------------------
VvCesA9 ------------------------------------------------------------
PbCesA ------------------------------------------------------------
VvCesA7 ------------------------------------------------------------
StCesA7 ------------------------------------------------------------
VvCesA1 ------------------------------------------------------------
ZmCesA2 ------------------------------------------------------------
VvCesA3 ------------------------------------------------------------
OsCesA2 ------------------------------------------------------------
AtCslB5 ------------------------------------------------------------
StCslH1 ------------------------------------------------------------
VvCslH1 ------------------------------------------------------------
VvCslB3 ------------------------------------------------------------
VvCslG2 ------------------------------------------------------------
AtCslG3 ------------------------------------------------------------
AtCslE1 ------------------------------------------------------------
VvCslE6 ------------------------------------------------------------
AtCslA3 ------------------------------------------------------------
AtCslA7 ------------------------------------------------------------
PbCCesA ------------------------------------------------------------
BaCesA ------------------------------------------------------------
AtmCesA ------------------------------------------------------------
RlCesA ------------------------------------------------------------
KxCesA3 1416 PQDVDTPLTGGVGAMIEAESPLTAGRTVLALLSSDGAGLNNLLQMLGERKKQANIQGDLV
EcCesA ------------------------------------------------------------
PsCesA ------------------------------------------------------------
PfCesA ------------------------------------------------------------
PvCesA3 ------------------------------------------------------------
PiCesA3 ------------------------------------------------------------
PvCesA4 ------------------------------------------------------------
PiCesA4 ------------------------------------------------------------
PvCesA2 ------------------------------------------------------------
PiCesA2 ------------------------------------------------------------
PvCesA1 ------------------------------------------------------------
PiCesA1 ------------------------------------------------------------
NsCesA ------------------------------------------------------------
TvCesA ------------------------------------------------------------
JfCesA ------------------------------------------------------------
TlCesA ------------------------------------------------------------
ThCesA ------------------------------------------------------------
GpCesA ------------------------------------------------------------
AmCesA ------------------------------------------------------------
AtCslD6 ------------------------------------------------------------
VvCslD3 ------------------------------------------------------------
CrCslD1 ------------------------------------------------------------
PpCslD8 ------------------------------------------------------------
AtCesA8 ------------------------------------------------------------
VvCesA8 ------------------------------------------------------------
VvCesA4 ------------------------------------------------------------
VvCesA5 ------------------------------------------------------------
AtCesA2 ------------------------------------------------------------
AtCesA6 ------------------------------------------------------------
StCesA1 ------------------------------------------------------------
HaCesA2 ------------------------------------------------------------
VvCesA6 ------------------------------------------------------------
VvCesA9 ------------------------------------------------------------
PbCesA ------------------------------------------------------------
VvCesA7 ------------------------------------------------------------
StCesA7 ------------------------------------------------------------
VvCesA1 ------------------------------------------------------------
ZmCesA2 ------------------------------------------------------------
VvCesA3 ------------------------------------------------------------
OsCesA2 ------------------------------------------------------------
AtCslB5 ------------------------------------------------------------
StCslH1 ------------------------------------------------------------
VvCslH1 ------------------------------------------------------------
VvCslB3 ------------------------------------------------------------
VvCslG2 ------------------------------------------------------------
AtCslG3 ------------------------------------------------------------
AtCslE1 ------------------------------------------------------------
VvCslE6 ------------------------------------------------------------
AtCslA3 ------------------------------------------------------------
AtCslA7 ------------------------------------------------------------
PbCCesA ------------------------------------------------------------
BaCesA ------------------------------------------------------------
AtmCesA ------------------------------------------------------------
RlCesA ------------------------------------------------------------
KxCesA3 1476 VAHGEDLSSYRTSPVYTIGTLPLWLWPDWYMHNRPVRVLLVGLLGCILIVSVLARALARH
EcCesA ------------------------------------------------------------
PsCesA ------------------------------------------------------------
PfCesA ------------------------------------------------------------
PvCesA3 ------------------------------------------------------------
PiCesA3 ------------------------------------------------------------
PvCesA4 ------------------------------------------------------------
PiCesA4 ------------------------------------------------------------
PvCesA2 ------------------------------------------------------------
PiCesA2 ------------------------------------------------------------
PvCesA1 ------------------------------------------------------------
PiCesA1 ------------------------------------------------------------
NsCesA ------------------------------------------------------------
TvCesA ------------------------------------------------------------
JfCesA ------------------------------------------------------------
TlCesA ------------------------------------------------------------
ThCesA ------------------------------------------------------------
GpCesA ------------------------------------------------------------
AmCesA ------------------------------------------------------------
AtCslD6 ---------------
VvCslD3 ---------------
CrCslD1 ---------------
PpCslD8 ---------------
AtCesA8 ---------------
VvCesA8 ---------------
VvCesA4 ---------------
VvCesA5 ---------------
AtCesA2 ---------------
AtCesA6 ---------------
StCesA1 ---------------
HaCesA2 ---------------
VvCesA6 ---------------
VvCesA9 ---------------
PbCesA ---------------
VvCesA7 ---------------
StCesA7 ---------------
VvCesA1 ---------------
ZmCesA2 ---------------
VvCesA3 ---------------
OsCesA2 ---------------
AtCslB5 ---------------
StCslH1 ---------------
VvCslH1 ---------------
VvCslB3 ---------------
VvCslG2 ---------------
AtCslG3 ---------------
AtCslE1 ---------------
VvCslE6 ---------------
AtCslA3 ---------------
AtCslA7 ---------------
PbCCesA ---------------
BaCesA ---------------
AtmCesA ---------------
RlCesA ---------------
KxCesA3 1536 AARRFKQLEDERRKS
EcCesA ---------------
PsCesA ---------------
PfCesA ---------------
PvCesA3 ---------------
PiCesA3 ---------------
PvCesA4 ---------------
PiCesA4 ---------------
PvCesA2 ---------------
PiCesA2 ---------------
PvCesA1 ---------------
PiCesA1 ---------------
NsCesA ---------------
TvCesA ---------------
JfCesA ---------------
TlCesA ---------------
ThCesA ---------------
GpCesA ---------------
AmCesA ---------------
