# Supplementary material for: NoPv1: a synthetic antimicrobial peptide aptamer targeting the causal agents of grapevine downy mildew and potato late blight
Source: Sci Rep. 2020 Oct 16;10:17574. doi: 10.1038/s41598-020-73027-x (PMC7567880; doi:10.1038/s41598-020-73027-x)
Supplement: Supplementary file 2 — Supplementary Data S2. [file 41598_2020_73027_MOESM2_ESM.docx]

**NoPv1: a synthetic antimicrobial peptide aptamer targeting the causal agents of grapevine downy mildew and potato late blight**

Monica Colombo^1+^, Simona Masiero^2+^, Stefano Rosa^2^, Elisabetta Caporali^2^, Silvia Laura Toffolatti^3^, Chiara Mizzotti^2^, Luca Tadini^2^, Fabio Rossi^4^, Sara Pellegrino^5^, Rita Musetti^6^, Riccardo Velasco^7^, Michele Perazzolli^1,8^, Silvia Vezzulli^1*^, Paolo Pesaresi^2*^

^1^ Research and Innovation Centre, Fondazione Edmund Mach, San Michele all'Adige, Italy.

^2^ Department of Biosciences, University of Milan, Milan, Italy.

^3^ Department of Agricultural and Environmental Sciences (DISAA), University of Milan, Milan, Italy.

^4^ Center for Study and Research on Obesity, Department of Medical Biotechnology and Translational Medicine, University of Milan, Milan, Italy.

^5^ DISFARM-Department of Pharmaceutical sciences, University of Milan, Milan, Italy.

^6^ Department of Agricultural, Food, Environmental and Animal Sciences, University of Udine, Udine, Italy

^7^ CREA Research Centre for Viticulture and Enology, Conegliano (TV), Italy

^8^ Centre Agriculture Food Environment (C3A), University of Trento, San Michele all’Adige, Italy

**^+^** These authors contributed equally to the article

** Co-corresponding authors: paolo.pesaresi@unimi.it; silvia.vezzulli@fmach.it*

**Running title:** NoPv1: a low-risk antimicrobial peptide

**Keywords**

*Antimicrobial peptides, Peptide aptamer*, *Pesticide, Phytophthora infestans*, *Plasmopara viticola*, *Vitis vinifera*, *Solanum tuberosum*

**Data S2. Structural characterization and purity analysis of NoPv1 peptide.**

**Structural characterization of NoPv1**

Amino acids sequence: RLTAQCRL

Molecular Formula: C**_39_**H_73_N_15_O_11_S

Molecular weight: 959.53

Chemical structure:

**High Resolution Mass (HRMS) analysis**

No-Pv1 was analyzed on ESI Q-Tof Mass Spectrometer SYNAPT G2-Si & MassLynx software (Waters). The deleted picks are due to interferences during the analysis and not impurities in the sample (see Keller et al., 2008: Interferences and contaminants encountered in modern mass spectrometry. Analytica Chimica Acta).

**
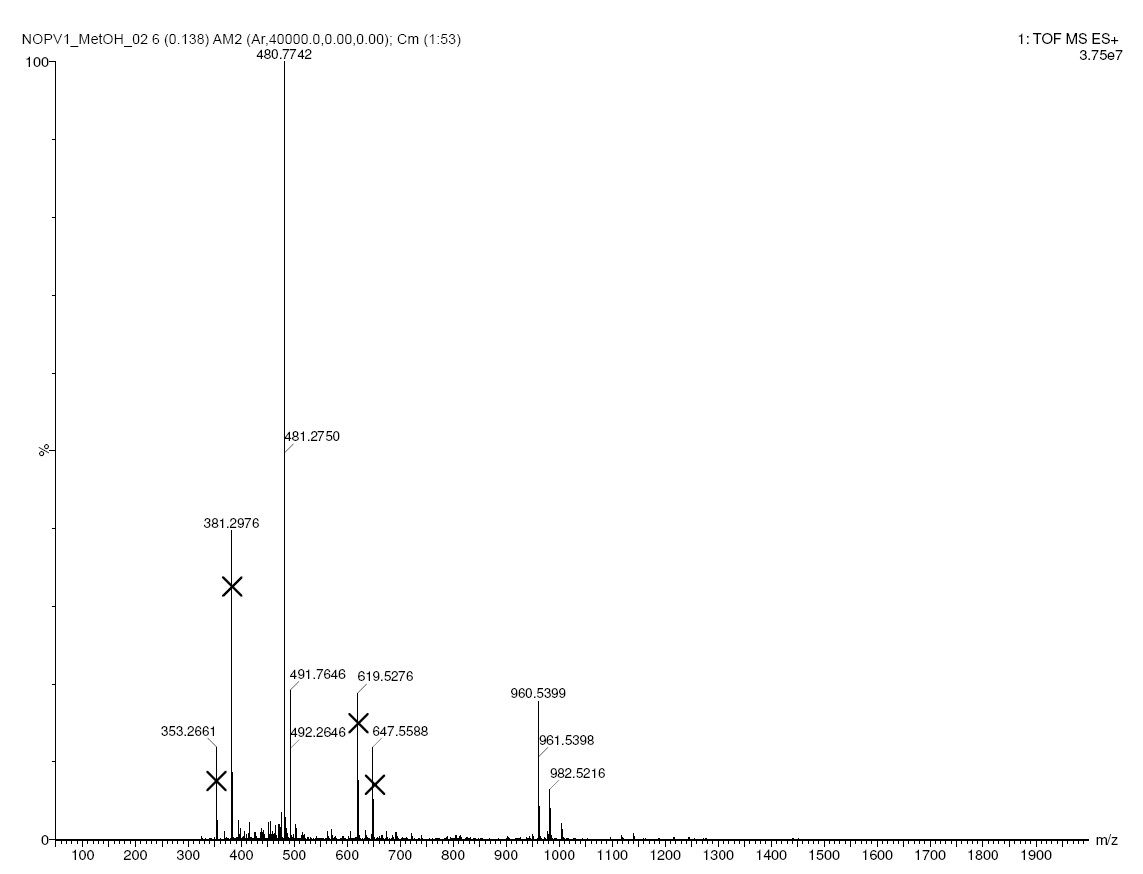
**

Observed picks:

[MW+ Na] ^+^= 982.5216

[MW+ H] ^+^= 960.5399

[MW+ Na]/2 ^+^= 491.7646

[MW+ H]/2 ^+^= 480.7742

Elemental composition reports from HRMS:

**NMR analysis**

^1^H NMR and ^13^C NMR spectra were recorded on a Bruker Avance 300 MHz and Gemini 300 MHz Varian spectrometer.

^1^H-NMR spectrum:

^13^C-NMR spectrum:

TOCSY experiment spectra:

Plot of Spin systems from TOCSY, with amino acids’assignments:

Leu

2 Arg

Ala

Thr

Leu, Gln, Cys

Guanidinium of Arg and amide of Gln

**HPLC analysis**

Leu

2 Arg

Ala

Thr

Leu, Gln, Cys

Guanidinium of Arg and amide of Gln
