# Supplementary material for: NoPv1: a synthetic antimicrobial peptide aptamer targeting the causal agents of grapevine downy mildew and potato late blight
Source: Sci Rep. 2020 Oct 16;10:17574. doi: 10.1038/s41598-020-73027-x (PMC7567880; doi:10.1038/s41598-020-73027-x)
Supplement: Supplementary file 3 — Supplementary Table S1. [file 41598_2020_73027_MOESM3_ESM.docx]

**NoPv1: a synthetic antimicrobial peptide aptamer targeting the causal agents of grapevine downy mildew and potato late blight**

Monica Colombo^1+^, Simona Masiero^2+^, Stefano Rosa^2^, Elisabetta Caporali^2^, Silvia Laura Toffolatti^3^, Chiara Mizzotti^2^, Luca Tadini^2^, Fabio Rossi^4^, Sara Pellegrino^5^, Rita Musetti^6^, Riccardo Velasco^7^, Michele Perazzolli^1,8^, Silvia Vezzulli^1*^, Paolo Pesaresi^2*^

^1^ Research and Innovation Centre, Fondazione Edmund Mach, San Michele all'Adige, Italy.

^2^ Department of Biosciences, University of Milan, Milan, Italy.

^3^ Department of Agricultural and Environmental Sciences (DISAA), University of Milan, Milan, Italy.

^4^ Center for Study and Research on Obesity, Department of Medical Biotechnology and Translational Medicine, University of Milan, Milan, Italy.

^5^ DISFARM-Department of Pharmaceutical sciences, University of Milan, Milan, Italy.

^6^ Department of Agricultural, Food, Environmental and Animal Sciences, University of Udine, Udine, Italy

^7^ CREA Research Centre for Viticulture and Enology, Conegliano (TV), Italy

^8^ Centre Agriculture Food Environment (C3A), University of Trento, San Michele all’Adige, Italy

**^+^** These authors contributed equally to the article

** Co-corresponding authors: paolo.pesaresi@unimi.it; silvia.vezzulli@fmach.it*

**Running title:** NoPv1: a low-risk antimicrobial peptide

**Keywords**

*Antimicrobial peptides, Peptide aptamer*, *Pesticide, Phytophthora infestans*, *Plasmopara viticola*, *Vitis vinifera*, *Solanum tuberosum*

**Table S1**. List of 54 cellulose synthase (CesA) and cellulose synthase-like (Csl) enzymes used for the phylogenetic analysis. Name, classification, organism and UniProtKB Entry code are indicated for each enzyme. The only exception is KJD55249.1 (BaCesA) which was obtained from the National Center for Biotechnology Information database (ncbi.nlm.nih.gov).

| Sequence input | Classification | Organism | UniProtKB Entry code |
| --- | --- | --- | --- |
| PvCesA1 | Cellulose synthase | *Plasmopara viticola* | D4N2S5 |
| PvCesA2 | Cellulose synthase | *Plasmopara viticola* | D4N2S6 |
| PvCesA3 | Cellulose synthase | *Plasmopara viticola* | D4N2S7 |
| PvCesA4 | Cellulose synthase | *Plasmopara viticola* | D4N2S8 |
| PiCesA1 | Cellulose synthase | *Phytophthora infestans* | A5A5Z3 |
| PiCesA2 | Cellulose synthase | *Phytophthora infestans* | A5A5Z4 |
| PiCesA3 | Cellulose synthase | *Phytophthora infestans* | A5A5Z5 |
| PiCesA4 | Cellulose synthase | *Phytophthora infestans* | A5A5Z6 |
| NsCesA | Cellulose synthase | *Nostoc sp.* | Q8YQR2 |
| TvCesA | Cellulose synthase | *Trichormus variabilis* | Q3MCU5 |
| AtCesA2 | Cellulose synthase | *Arabidopsis thaliana* | O48947 |
| AtCesA6 | Cellulose synthase | *Arabidopsis thaliana* | Q94JQ6 |
| AtCesA8 | Cellulose synthase | *Arabidopsis thaliana* | Q8LPK5 |
| VvCesA1 | Cellulose synthase | *Vitis vinifera* | A0A438KEU9 |
| VvCesA3 | Cellulose synthase | *Vitis vinifera* | A0A438J1N1 |
| VvCesA4 | Cellulose synthase | *Vitis vinifera* | A0A438F0U9 |
| VvCesA5 | Cellulose synthase | *Vitis vinifera* | A0A438K476 |
| VvCesA6 | Cellulose synthase | *Vitis vinifera* | A0A438JWC7 |
| VvCesA7 | Cellulose synthase | *Vitis vinifera* | A0A438IHR5 |
| VvCesA8 | Cellulose synthase | *Vitis vinifera* | A0A438D8W3 |
| VvCesA9 | Cellulose synthase | *Vitis vinifera* | A0A438EM69 |
| PbCesA | Cellulose synthase | *Physcomitrella patens* | A0A2K1K2V6 |
| HaCesA2 | Cellulose synthase | *Populus tremula x Populus tremuloides* (Hybrid aspen) | Q6J8X1 |
| StCesA1 | Cellulose synthase | *Solanum tuberosum* | Q6XP47 |
| StCesA7 | Cellulose synthase | *Solanum tuberosum* | M1ARZ5 |
| ZmCesA2 | Cellulose synthase | *Zea mays* | Q9LLI8 |
| OsCesA2 | Cellulose synthase | *Oryza sativa* | Q84M43 |
| AtmCesA | Cellulose synthase | *Agrobacterium tumefaciens* | A0A2L2LG53 |
| EcCesA | Cellulose synthase | *Escherichia coli* | P37653 |
| KxCesA3 | Cellulose synthase | *Komagataeibacter xylinus* | Q76KJ8 |
| RlCesA | Cellulose synthase | *Rhizobium leguminosarum* | Q1MIR9 |
| PsCesA | Cellulose synthase | *Pseudomonas syringae* | A0A0P9P576 |
| PfCesA | Cellulose synthase | *Pseudomonas fluorescens* | P58931 |
| BaCesA | Cellulose synthase | *Bacillus amyloliquefaciens* | KJD55249.1^¶^ |
| PbCCesA | Cellulose synthase | *Paramecium bursaria Chlorella* virus 1 | M1HL66 |
| TlCesA | Cellulose synthase | *Trichoderma longibrachiatum* | A0A2T4CBB4 |
| ThCesA | Cellulose synthase | *Trichoderma harzianum* | A0A2K0UGU2 |
| JfCesA | Cellulose synthase | *Jimgerdemannia flammicorona* | A0A433QUT4 |
| GpCesA | Cellulose synthase | *Gonapodya prolifera* | A0A139B145 |
| AmCesA | Cellulose synthase | *Allomyces macrogynus* | A0A0L0S907 |
| AtCslA3 | Cellulose synthase-like A | *Arabidopsis thaliana* | A0A1P8ANC3 |
| AtCslA7 | Cellulose synthase-like A | *Arabidopsis thaliana* | A0A1P8B2I3 |
| AtCslB5 | Cellulose synthase-like B | *Arabidopsis thaliana* | Q0WT40 |
| AtCslG3 | Cellulose synthase-like G | *Arabidopsis thaliana* | Q0WVN5 |
| AtCslD6 | Cellulose synthase-like D | *Arabidopsis thaliana* | Q9FVR3 |
| AtCslE1 | Cellulose synthase-like E | *Arabidopsis thaliana* | Q8VZK9 |
| VvCslD3 | Cellulose synthase-like D | *Vitis vinifera* | A0A438GKG4 |
| VvCslG2 | Cellulose synthase-like G | *Vitis vinifera* | A0A438F7V3 |
| VvCslH1 | Cellulose synthase-like H | *Vitis vinifera* | A0A438IKZ5 |
| VvCslE6 | Cellulose synthase-like E | *Vitis vinifera* | A0A438HRT8 |
| VvCslB3 | Cellulose synthase-like B | *Vitis vinifera* | A0A438DTY2 |
| StCslH1 | Cellulose synthase-like H | *Solanum tuberosum* | M1BFL3 |
| CrCslD1 | Cellulose synthase-like D | *Ceratopteris richardii* | Q6GVL6 |
| PpCslD8 | Cellulose synthase-like D | *Physcomitrella patens* | A9SDL9 |
