# Supplementary material for: NoPv1: a synthetic antimicrobial peptide aptamer targeting the causal agents of grapevine downy mildew and potato late blight
Source: Sci Rep. 2020 Oct 16;10:17574. doi: 10.1038/s41598-020-73027-x (PMC7567880; doi:10.1038/s41598-020-73027-x)
Supplement: Supplementary file 4 — Supplementary Table S2. [file 41598_2020_73027_MOESM4_ESM.docx]

**NoPv1: a synthetic antimicrobial peptide aptamer targeting the causal agents of grapevine downy mildew and potato late blight**

Monica Colombo^1+^, Simona Masiero^2+^, Stefano Rosa^2^, Elisabetta Caporali^2^, Silvia Laura Toffolatti^3^, Chiara Mizzotti^2^, Luca Tadini^2^, Fabio Rossi^4^, Sara Pellegrino^5^, Rita Musetti^6^, Riccardo Velasco^7^, Michele Perazzolli^1,8^, Silvia Vezzulli^1*^, Paolo Pesaresi^2*^

^1^ Research and Innovation Centre, Fondazione Edmund Mach, San Michele all'Adige, Italy.

^2^ Department of Biosciences, University of Milan, Milan, Italy.

^3^ Department of Agricultural and Environmental Sciences (DISAA), University of Milan, Milan, Italy.

^4^ Center for Study and Research on Obesity, Department of Medical Biotechnology and Translational Medicine, University of Milan, Milan, Italy.

^5^ DISFARM-Department of Pharmaceutical sciences, University of Milan, Milan, Italy.

^6^ Department of Agricultural, Food, Environmental and Animal Sciences, University of Udine, Udine, Italy

^7^ CREA Research Centre for Viticulture and Enology, Conegliano (TV), Italy

^8^ Centre Agriculture Food Environment (C3A), University of Trento, San Michele all’Adige, Italy

**^+^** These authors contributed equally to the article

** Co-corresponding authors: paolo.pesaresi@unimi.it; silvia.vezzulli@fmach.it*

**Running title:** NoPv1: a low-risk antimicrobial peptide

**Keywords**

*Antimicrobial peptides, Peptide aptamer*, *Pesticide, Phytophthora infestans*, *Plasmopara viticola*, *Vitis vinifera*, *Solanum tuberosum*

**Table S2.** Maximum quantum yield (F_V_/F_M_) and effective quantum yield [Y_(II)_] of photosystem II values referring to the images shown in Figure 3. Measurements were performed on grapevine leaves 7 days after treatment with 400 µM (P1) and 1 mM (P2) of NoPv1, 0.1% v/v (BH1) and 0.2% v/v (BH2) of BASTA herbicide and water (M1 and M2). Average and standard deviation values of 3 independent measurements performed inside each of the 6 circles are shown.

|  | **M1** | **BH1** | **BH2** | **M1** | **P1** | **P2** |
| --- | --- | --- | --- | --- | --- | --- |
| **F_V_/F_M_** | 0.74 ± 0.01 | 0.63 ± 0.03 | 0.47 ± 0.11 | 0.74 ± 0.01 | 0.73 ± 0.01 | 0.74 ± 0.01 |
| **Y_(II)_** | 0.25 ± 0.01 | 0.2 ± 0.03 | 0.13 ± 0.11 | 0.27 ± 0.01 | 0.33 ± 0.02 | 0.33 ± 0.01 |
