# Supplementary material for: NoPv1: a synthetic antimicrobial peptide aptamer targeting the causal agents of grapevine downy mildew and potato late blight
Source: Sci Rep. 2020 Oct 16;10:17574. doi: 10.1038/s41598-020-73027-x (PMC7567880; doi:10.1038/s41598-020-73027-x)
Supplement: Supplementary file 5 — Supplementary Table S3. [file 41598_2020_73027_MOESM5_ESM.docx]

**NoPv1: a synthetic antimicrobial peptide aptamer targeting the causal agents of grapevine downy mildew and potato late blight**

Monica Colombo^1+^, Simona Masiero^2+^, Stefano Rosa^2^, Elisabetta Caporali^2^, Silvia Laura Toffolatti^3^, Chiara Mizzotti^2^, Luca Tadini^2^, Fabio Rossi^4^, Sara Pellegrino^5^, Rita Musetti^6^, Riccardo Velasco^7^, Michele Perazzolli^1,8^, Silvia Vezzulli^1*^, Paolo Pesaresi^2*^

^1^ Research and Innovation Centre, Fondazione Edmund Mach, San Michele all'Adige, Italy.

^2^ Department of Biosciences, University of Milan, Milan, Italy.

^3^ Department of Agricultural and Environmental Sciences (DISAA), University of Milan, Milan, Italy.

^4^ Center for Study and Research on Obesity, Department of Medical Biotechnology and Translational Medicine, University of Milan, Milan, Italy.

^5^ DISFARM-Department of Pharmaceutical sciences, University of Milan, Milan, Italy.

^6^ Department of Agricultural, Food, Environmental and Animal Sciences, University of Udine, Udine, Italy

^7^ CREA Research Centre for Viticulture and Enology, Conegliano (TV), Italy

^8^ Centre Agriculture Food Environment (C3A), University of Trento, San Michele all’Adige, Italy

**^+^** These authors contributed equally to the article

** Co-corresponding authors: paolo.pesaresi@unimi.it; silvia.vezzulli@fmach.it*

**Running title:** NoPv1: a low-risk antimicrobial peptide

**Keywords**

*Antimicrobial peptides, Peptide aptamer*, *Pesticide, Phytophthora infestans*, *Plasmopara viticola*, *Vitis vinifera*, *Solanum tuberosum*

**Table S3.** Disease severity data of preventive and curative properties of NoPv1 against *Plasmopara viticola* on leaf disks.

| **Treatment** | **Time point** | **Disease severity** | **Standard error** |
| --- | --- | --- | --- |
| Control | -7 d | 43.20 | 4.41 |
| NoPv1 | -7 d | 9.72 | 3.49 |
| Control | -5 d | 34.56 | 8.93 |
| NoPv1 | -5 d | 2.23 | 0.63 |
| Control | -2 d | 54.00 | 5.69 |
| NoPv1 | -2 d | 3.96 | 1.75 |
| Control | -1 d | 43.20 | 9.00 |
| NoPv1 | -1 d | 6.17 | 2.72 |
| Control | -6 h | 36.00 | 0.00 |
| NoPv1 | -6 h | 5.62 | 1.50 |
| Control | -2 h | 38.16 | 4.20 |
| NoPv1 | -2 h | 1.58 | 0.70 |
| Control | 0 h | 52.56 | 6.89 |
| NoPv1 | 0 h | 2.88 | 1.27 |
| Control | +1 h | 50.40 | 3.60 |
| NoPv1 | +1 h | 18.00 | 4.96 |
| Control | +1 d | 43.20 | 4.41 |
| NoPv1 | +1 d | 23.76 | 5.17 |
| Control | +2 d | 43.92 | 8.40 |
| NoPv1 | +2 d | 36.00 | 2.28 |

The anti-oomycete activity of NoPv1 (400 μM) was tested, using the Potter Precision Spray Tower, on leaf disks at different time points [days (d)/hours (h)] before (from -7 d to -2 h) and after (+1 h to +2 d) *Plasmopara viticola* inoculation. The co-inoculation assay (0 h) is also reported. Disease severity was evaluated at seven days post inoculation. For each treatment, average and standard error values of ten replicates from two experiments are presented. The average ± standard error values of the disease severity of control plants at all time points was 43.2 ± 4.4%.
