# Supplementary material for: NoPv1: a synthetic antimicrobial peptide aptamer targeting the causal agents of grapevine downy mildew and potato late blight
Source: Sci Rep. 2020 Oct 16;10:17574. doi: 10.1038/s41598-020-73027-x (PMC7567880; doi:10.1038/s41598-020-73027-x)
Supplement: Supplementary file 6 — Supplementary Table S4. [file 41598_2020_73027_MOESM6_ESM.docx]

**NoPv1: a synthetic antimicrobial peptide aptamer targeting the causal agents of grapevine downy mildew and potato late blight**

Monica Colombo^1+^, Simona Masiero^2+^, Stefano Rosa^2^, Elisabetta Caporali^2^, Silvia Laura Toffolatti^3^, Chiara Mizzotti^2^, Luca Tadini^2^, Fabio Rossi^4^, Sara Pellegrino^5^, Rita Musetti^6^, Riccardo Velasco^7^, Michele Perazzolli^1,8^, Silvia Vezzulli^1*^, Paolo Pesaresi^2*^

^1^ Research and Innovation Centre, Fondazione Edmund Mach, San Michele all'Adige, Italy.

^2^ Department of Biosciences, University of Milan, Milan, Italy.

^3^ Department of Agricultural and Environmental Sciences (DISAA), University of Milan, Milan, Italy.

^4^ Center for Study and Research on Obesity, Department of Medical Biotechnology and Translational Medicine, University of Milan, Milan, Italy.

^5^ DISFARM-Department of Pharmaceutical sciences, University of Milan, Milan, Italy.

^6^ Department of Agricultural, Food, Environmental and Animal Sciences, University of Udine, Udine, Italy

^7^ CREA Research Centre for Viticulture and Enology, Conegliano (TV), Italy

^8^ Centre Agriculture Food Environment (C3A), University of Trento, San Michele all’Adige, Italy

**^+^** These authors contributed equally to the article

** Co-corresponding authors: paolo.pesaresi@unimi.it; silvia.vezzulli@fmach.it*

**Running title:** NoPv1: a low-risk antimicrobial peptide

**Keywords**

*Antimicrobial peptides, Peptide aptamer*, *Pesticide, Phytophthora infestans*, *Plasmopara viticola*, *Vitis vinifera*, *Solanum tuberosum*

**Table S4.** Disease severity data of preventive and curative properties of NoPv1 against *Plasmopara viticola* on greenhouse grown plants.

| **Treatment** | **Time point** | **Disease severity** | **Standard error** |
| --- | --- | --- | --- |
| NoPv1 | -7 d | 15.89 | 2.6 |
| NoPv1 | -2 d | 21.50 | 2.2 |
| NoPv1 | 0 h | 20.56 | 2.4 |
| Control |  | 58.15 | 6.6 |

The NoPv1 peptide at 1 mM concentration was sprayed at seven and two days before *Plasmopara viticola* inoculation (-7 d and -2 d) or co-inoculated (0 h) with *P. viticola*, and the disease severity was assessed at seven days post inoculation. For each treatment, average and standard error values of ten replicates from two experiments are presented.
